# *CircTspan3* Promotes Cartilage Development Through ANNEXIN A2‐Mediated Ferroptosis and Apoptosis Inhibition and Exosome‐Mediated Paracrine Signaling

**DOI:** 10.1002/advs.202513418

**Published:** 2026-01-04

**Authors:** Yiming Pan, Fengmei Zhang, Jiayan Zhong, Qian Gong, Nana Geng, Biao Kuang, Qiumei Lan, Miao Yi, Qiqi Zeng, Cheng Chen, Mengtian Fan, Chunliang Zhao, Deping Zeng, Yu Du, Mao Nie, Zhibiao Wang, Fengjin Guo

**Affiliations:** ^1^ Department of Orthopedics State Key Laboratory of Ultrasound in Medicine and Engineering School of Basic Medical Sciences The Second Affiliated Hospital of Chongqing Medical University Chongqing Medical University Chongqing China; ^2^ Department of Orthopedics The Second Affiliated Hospital of Chongqing Medical University, Chongqing Medical University Chongqing China; ^3^ Department of Orthopedics The First Affiliated Hospital of Chongqing Medical University, Chongqing Medical University Chongqing China; ^4^ State Key Laboratory of Ultrasound in Medicine and Engineering Chongqing Medical University Chongqing China

**Keywords:** ANXA2, apoptosis, cartilage development, circTspan3, exosomes, ferroptosis, P‐ANXA2 (Ser26), Xbp1s

## Abstract

Circular RNAs (circRNAs) are covalently closed, stable non‐coding RNAs that regulate diverse cellular processes. Here, we identify *circTspan3* — derived from exons 2–6 of the *Tspan3* gene — as a key regulator of cartilage development. The expression of *circTspan3* is significantly downregulated in *X‐box binding protein 1* (*Xbp1*) conditional knockout (cKO) mice displaying chondrodysplasia and positively correlates with anabolic markers of cartilage. The XBP1 spliced (XBP1s) transcriptionally upregulates *circTspan3*, which in turn promotes anabolic activity in chondrocytes while suppressing both apoptosis and ferroptosis. Mechanistically, phosphorylation of ANNEXIN A2 (ANXA2) at Ser26 facilitates the cytoplasmic translocation of *circTspan3*, where ANXA2 mediates its packaging into exosomes for paracrine signalling. Exosomal *circTspan3* enhances growth‐plate expansion and effectively repairs cartilage defects in vivo. These findings highlight *circTspan3* as a key modulator of growth‐plate homeostasis and suggest its translational potential in treating cartilage injury and growth‐associated skeletal disorders.

## Graphical abstract

This study reveals that XBP1s drives production of *circTspan3*, a circular RNA that strengthens cartilage by boosting anabolic activity and limiting cell death. Phosphorylated ANXA2 directs circTspan3 into exosomes, enabling paracrine repair. Exosomal *circTspan3* expands growth‐plate cartilage and promotes in vivo regeneration, highlighting its promise for treating skeletal disorders.

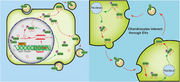

## 1 Introduction

Endochondral ossification—the process driving longitudinal growth of long bones—relies on exquisitely coordinated chondrocyte proliferation, hypertrophy, extracellular matrix (ECM) synthesis, and timely cell‐death programs [1, 2, 3, 4]. Disruption of any of these steps underpins a spectrum of skeletal dysplasias and post‐traumatic cartilage defects that remain clinically intractable. Recent work has implicated the unfolded protein response (UPR) transcription factor X‐box binding protein‐1 (spliced isoform, XBP1s) as a gatekeeper of chondrocyte physiology [5, 6]; cartilage‐specific ablation of *Xbp1* arrests growth‐plate expansion and delays ECM mineralization [6, 7, 8, 9]. Yet the downstream effectors that translate ER‐stress cues into anabolic or catabolic outcomes in cartilage are incompletely defined, particularly in the realm of non‐coding RNAs.

Circular RNAs (circRNAs)—covalently closed, exonuclease‐resistant transcripts—have emerged as potent post‐transcriptional regulators and promising therapeutic cargos [10, 11, 12]. Their abundance, tissue specificity, and propensity to be packaged into exosomes make circRNAs attractive candidates for paracrine communication [13, 14, 15].

Recent advances (2019–2024) have identified several cartilage‐protective circRNAs, yet most target singular pathological nodes with limited mechanistic overlap. Shen et al. demonstrated that *circSERPINE2* reduces chondrocyte apoptosis and matrix degradation via the miR‐1271‐ERG axis, effectively protecting cartilage in preclinical models [16]. Wu et al. showed that *circPDE4D* preserves ECM homeostasis through the miR‐103a‐3p‐FGF18 pathway, though with minimal impact on apoptosis [17]. Similarly, Shang et al. reported that *circHIPK3* curbs mitochondrial reactive oxygen species (ROS) and apoptosis by sponging miR‐30a‐3p to elevate PON2 [18], while Lai et al. found that *circCDK14* counteracts IL‐1β‐induced apoptosis through the miR‐1183/KLF5 axis [19]. Notably, direct connections between circRNAs and ferroptosis—an iron‐dependent form of regulated cell death increasingly implicated in cartilage degeneration [20, 21]—have remained sparse until recently. He et al. provided initial evidence that silencing *circTRIM25* attenuates chondrocyte ferroptosis via the miR‐138‐5p/CREB1 pathway [22], yet comprehensive studies addressing both apoptosis and ferroptosis simultaneously are lacking.

Ferroptosis has emerged as a critical pathogenic mechanism in osteoarthritis (OA), driven by iron overload, lipid peroxidation, and glutathione peroxidase 4 (GPX4) inactivation [23, 24, 25]. Recent studies demonstrate that mechanical overloading induces GPX4‐regulated chondrocyte ferroptosis through Piezo1‐mediated calcium influx [26], while P21 resists ferroptosis by stabilizing GPX4 protein [27]. Moreover, systemic factors such as gut microbiota‐derived metabolites can modulate cartilage ferroptosis through HIF‐1α/SLC2A1 signaling [28]. These findings underscore that ferroptosis and apoptosis frequently coexist in degenerating cartilage, necessitating therapeutic strategies that address both pathways concurrently.

Against this backdrop, our study identifies *circTspan3* as a unique circRNA that simultaneously suppresses both apoptosis and ferroptosis in stressed chondrocytes, thereby addressing two death modalities increasingly recognized as synergistic drivers of OA pathogenesis [20, 21, 24]. Moreover, we establish that *circTspan3* is directly transcriptionally regulated by XBP1s — a master ER stress responder — revealing a previously unrecognized integration point between proteostasis networks and non‐coding RNA‐mediated cartilage homeostasis. Nonetheless, the identity, mechanisms of biogenesis, and functional significance of ER stress‐responsive circRNAs in chondrocyte biology have remained largely unexplored.

ANNEXIN A2 (ANXA2) is a Ca^2^⁺‐dependent phospholipid‐binding protein that links membrane curvature/lipid microdomains to actin dynamics and vesicle traffic. In articular cartilage, ANXA2 and other annexins are abundant within matrix vesicles released by chondrocytes; these vesicles nucleate mineral deposition, and their aberrant activity is associated with osteoarthritic calcification and extracellular matrix remodeling, underscoring a direct connection to cartilage homeostasis [29, 30, 31]. Beyond matrix vesicles, ANXA2 is repeatedly identified within exosomes and participates in endosomal–exosomal routing: it associates with lipid‐raft endocytosis and multivesicular bodies, and autophagy—exosome crosstalk (amphisomes) can sort ANXA2 into secreted vesicles via RAB11/8A/27A‐dependent steps [32, 33, 34]. Functionally, exosomal ANXA2 can modulate the tissue microenvironment (e.g., pro‐angiogenic signaling), further highlighting its role as an active exosomal cargo [34, 35]. Importantly, N‐terminal serine phosphorylation of ANXA2 (Ser25/26, species‐dependent numbering) regulates its subcellular localization and association with messenger ribonucleoprotein (mRNP) complexes, providing a plausible mechanistic link between ANXA2, RNA handling, and vesicular export pathways [36]. These reports collectively position ANXA2 as a cartilage‐relevant organizer of vesicular trafficking, thereby supporting our focus on ANXA2‐dependent circRNA routing in chondrocytes.

Here, we identify *circTspan3*, generated from exons 2–6 of *Tspan3*, as an XBP1s‐responsive circRNA that orchestrates cartilage development and repair. We demonstrate that XBP1s binds the *Tspan3* promoter, driving the transcription of both its linear and circular isoforms. Notably, *circTspan3* expression strongly correlates with anabolic markers such as SOX9, COL2A1, and AGGRECAN in murine growth plates. Mechanistically, *circTspan3* exerts cell‐intrinsic effects by suppressing apoptosis and ferroptosis—two cell‐death pathways increasingly recognized as culprits in cartilage degeneration—thereby preserving chondrocyte viability and supporting ECM synthesis [21, 24, 37, 38, 39].

Beyond its intracellular functions, we show that *circTspan3* is selectively exported via exosomes through a phospho‐ANXA2 (Ser26)—dependent mechanism. Phosphorylation at Ser26 licenses ANXA2 to escort *circTspan3* from the nucleus to the cytoplasm, where ANXA2 further facilitates its incorporation into exosomes. These *circTspan3‐*enriched exosomes are efficiently internalized by recipient chondrocytes, where they enhance anabolic gene expression and mitigate stress‐induced cell death. Harnessing this paracrine axis, we formulate an injectable chitosan‐based hydrogel loaded with *circTspan3*‐enriched exosomes that achieves near‐complete regeneration of murine cartilage defects and restores growth‐plate architecture in vivo.

Collectively, our work establishes a previously unrecognized XBP1s—*circTspan3* –ANXA2 signaling axis that couples ER‐stress adaptation to circRNA trafficking and cartilage homeostasis. By integrating molecular genetics, RNA biology, and biomaterial engineering, we provide both mechanistic insights and a rapidly translatable therapeutic strategy for cartilage regeneration.

## 2 Materials and Methods

### 2.1 Mice

Professors Qingbo Xu (BHF Centre, King's College London, UK) and Lin Chen (Army Medical University, Chongqing, China) provided the *Xbp1* ^flox/flox^ and *Col2*‐Cre mice [40], respectively. Genotyping of transgenic mice was performed by PCR using genomic DNA isolated from tail biopsies. All mice were housed in a specific pathogen‐free facility under a 12 h light/dark cycle, 50%–60% relative humidity, and an ambient temperature of 22–24°C, with no more than five animals per cage. Mice had free access to tap water and standard rodent chow unless otherwise stated. Group sizes ranged from three to seventeen animals, with the exact number indicated by the data points in each figure legend. No adverse events were observed. Littermate controls from embryonic day 18 to 8 weeks of age were used for comparative analyses, and both sexes were included. Animals were euthanised using CO_2_, and body weight and length (from the skull base to the tail base) were recorded.

### 2.2 Chondrocyte Isolation and Culture

Primary chondrocytes were isolated from the knee cartilage of 7‐day‐old mice as previously described [41]. Cartilage samples were carefully dissected under a stereomicroscope and washed three times in PBS. The tissue was incubated overnight (12–16 h) at 37°C in digestion buffer containing 0.25 % collagenase II in DMEM. After digestion, the cell suspension was transferred to 60 mm culture dishes and maintained for three days in DMEM/F12 medium supplemented with 10 % FBS and 100 U mL^−1^ penicillin–streptomycin. The medium was refreshed every 48 h. Primary chondrocytes cultured for fewer than 24 h in vitro were designated P0, and cells were sub‐cultured once population doubling was achieved for further experiments.

### 2.3 Whole‐Transcriptome RNA Sequencing and circRNA Identification

Total RNA was extracted from cartilage of *Xbp1* conditional knockout (cKO) and *Xbp1^flox/flox^* mice as previously described [40]. Three littermate pairs (*Xbp1^flox/flox^ Col2*CRE^+^ vs. *Xbp1^flox/flox^ Col2*CRE^−^) were euthanized at four weeks of age, and their hyaline cartilage was processed for whole‐transcriptome RNA sequencing. circRNA expression profiles were analysed by RiboBio Co., Ltd. (Guangzhou, China). Ribosomal and linear RNAs were removed using the RiboBio rRNA‐depletion kit and RNase R treatment. The remaining circRNAs were reverse‐transcribed into cDNA and sequenced on an Illumina HiSeq 3000 system. circRNAs with *p* < 0.05 and fold change > 2 were considered differentially expressed.

For prioritization of downstream validation targets, candidate circRNAs were systematically evaluated based on four criteria: (i) Magnitude of differential expression: circRNAs were ranked by log_2_ fold‐change and statistical significance (adjusted *p*‐value), with priority given to circRNAs in the top 10% of downregulated candidates showing adjusted *p* < 0.01. (ii) Functional annotation of host genes: Host gene identity was determined using circBase (http://www.circbase.org/), and their relevance to skeletal or cartilage biology was assessed through Gene Ontology enrichment analysis (http://geneontology.org/) and literature review in PubMed, with preference for genes involved in chondrocyte differentiation, extracellular matrix assembly, membrane trafficking, or signal transduction pathways known to regulate cartilage homeostasis. (iii) Expression abundance and developmental dynamics: Candidate circRNAs were validated by RT‐qPCR in independent cartilage samples across developmental stages (embryonic day 18 to postnatal week 3), with selection criteria requiring detectable expression increase during active endochondral ossification. (iv) Correlation with cartilage phenotype: Pearson correlation coefficients were calculated between candidate circRNA expression levels and established cartilage anabolic markers (*Sox9*, *Col2a1*, *Aggrecan*) and catabolic markers (*Mmp13*, *Adamts4*, *Adamts5*) using RNA‐seq data from all biological replicates. circRNAs showing positive correlation with anabolic markers but no significant correlation with catabolic markers were prioritized for functional validation. *CircTspan3* (mmu_circ_0001775) was selected as the primary validation target as it uniquely satisfied all four criteria.

### 2.4 RNA Pull‐Down Assay

Synthesized *circTspan3* (full‐length 606 nt, exons 2–6 junction sequence) and its biotin‐labeled antisense probes were ordered from Sangon Biotech (Shanghai, China)(Table S1 A scrambled RNA sequence of equal length with similar GC content served as a negative control. Primary chondrocytes (P0‐P2; 1 × 10⁷ cells per sample) were lysed in IP lysis buffer (Pierce) supplemented with protease inhibitor cocktail (Roche) and RNase inhibitor (40 U/mL, Promega). Lysates were pre‐cleared by centrifugation (12 000 × g, 10 min, 4°C) and incubated with 3 µg of biotinylated probes overnight at 4°C with gentle rotation. The biotin‐coupled RNA‐protein complexes were captured using streptavidin magnetic beads (Life Technologies, USA) for 4 h at 4°C, followed by five stringent washes with IP wash buffer (20 mm Tris‐HCl, pH 7.5, 150 mm NaCl, 0.5% NP‐40, 1 mm EDTA). Bound proteins were eluted by boiling the beads in 1× SDS loading buffer (95°C, 5 min). For mass spectrometry identification, eluates from three independent pull‐down experiments (*circTspan3* probe and scrambled probe in parallel) were pooled, separated by SDS‐PAGE, and subjected to in‐gel trypsin digestion followed by liquid chromatography‐tandem mass spectrometry (LC‐MS/MS) analysis on a Q Exactive HF mass spectrometer (Thermo Fisher Scientific). Proteins identified with ≥2 unique peptides, false discovery rate (FDR) < 0.01, and ≥2‐fold enrichment *in circTspan3* pull‐down versus scrambled probe control were considered high‐confidence interactors. For validation experiments, eluates were analyzed by Western blotting using anti‐ANXA2 antibody (1:1000, Proteintech, 11256‐1‐AP).

### 2.5 RNA Immunoprecipitation (RIP)

RIP assay was performed using the RNA Immunoprecipitation Kit (GENESEED, P0101, China) according to the manufacturer's instructions to extract RNA entities bound to ANXA2 with an ANXA2‐specific antibody. The expression levels of *circTspan3* were quantified using reverse transcription followed by real‐time quantitative polymerase chain reaction (RT‐qPCR) analysis.

### 2.6 Reactive Oxygen Species (ROS) Evaluation

Intracellular ROS levels in chondrocytes were assessed using MitoSOX Red (HY‐D1055, MCE, USA). Cells (4 × 10^8^ per well) were seeded into six‐well plates and transfected with the indicated plasmids for 48 h. The staining solution (10 µmol L^−1^ final concentration) was prepared by diluting the reagent 1:1000 in serum‐free medium. After a 30 min incubation at 37°C in the dark, cells were washed three times with serum‐free medium, and ROS levels were examined by immunofluorescence analysis.

### 2.7 Dual‐Timepoint Pulse‐Chase Experiment

Primary mouse chondrocytes (P0, 60%–80% confluence) were cultured in DMEM/F12 with 10% FBS at 37°C. To dissect linear versus circular *Tspan3* function, we performed tunicamycin pulse‐chase with actinomycin D blockade. Four groups (*n* = 5 biological replicates each) were established: (G1) vehicle control (0.1% DMSO); (G2) actinomycin D alone (5 µg/mL); (G3) tunicamycin pulse (0.75 µg/mL, 90 min) followed by washout; (G4) tunicamycin pulse followed by actinomycin D addition. Samples were collected at 2.5 and 9 h post‐treatment. Total RNA was extracted using TRIzol, treated with DNase I, and quantified by RT‐qPCR.

### 2.8 Statistical Analysis

All analyses were prespecified and performed in GraphPad Prism v10 (and SPSS was noted). Data are reported as mean ± SEM, and n denotes biological replicates (independent cell preparations, litters, or animals); technical replicates were averaged within each biological replicate and do not contribute to n. All tests were two‐sided with α = 0.05. Before hypothesis testing, raw data were screened for prespecified technical failures; normality of model residuals was assessed (Shapiro–Wilk with Q–Q inspection), and homoscedasticity was evaluated (Brown–Forsythe/Levene). Skewed variables (e.g., ratios such as GSH/GSSG) were log‐transformed for analysis, with back‐transformed summaries in plots. Paired designs—i.e., cells from the same mouse aliquoted into two conditions or repeated measures on the same biological unit—were analyzed as matched data: for two conditions, we used two‐sided paired *t*‐tests (or Wilcoxon signed‐rank if residuals were non‐normal); for > 2 conditions/time points, we used repeated‐measures ANOVA or a mixed‐effects model with a random intercept for mouse (Geisser–Greenhouse correction applied when sphericity was violated). Unpaired designs used unpaired Student's *t*‐tests (Welch's correction when variances were unequal) for two groups; for ≥ 3 groups (single factor) with assumptions met, one‐way ANOVA with Tukey (all pairwise) or Dunnett (vs. a prespecified control) post hoc tests; when variance homogeneity was violated, Welch's ANOVA with Games–Howell post hoc was used. Non‐parametric procedures were applied when normality and/or homoscedasticity were not satisfied, or outcomes were ordinal (e.g., histological scores), specifically Kruskal–Wallis with pairwise Mann–Whitney U tests and Benjamini–Hochberg FDR control within the figure panel (e.g., Figure 6G). Multiplicity was controlled within each panel using the corresponding post hoc/adjustment (Tukey/Šidák/Dunnett/Games–Howell or BH‐FDR). Correlations used Pearson's r when both variables were approximately normal (after transform if needed) and Spearman's ρ otherwise, with FDR control for correlation families. RNA‐seq differential expression was analyzed with DESeq2 and reported as adjusted P (FDR) < 0.05. Normalization followed assay standards (e.g., Western blots to the stated loading control or Input; EV cargo to particle number; RT‐qPCR by ΔΔCt with specified reference genes; circRNAs quantified with divergent primers and −RT controls). Each figure legend specifies n (biological replicates), data presentation (mean ± SEM), the exact statistical test (including two‐sided), any post hoc/adjustment, and *p*‐values or symbols.

## 3 Results

### 3.1 Cartilage‐Specific Knockout of Xbp1 Signaling in Mouse Results in a Chondrodysplasia

**FIGURE 1:**
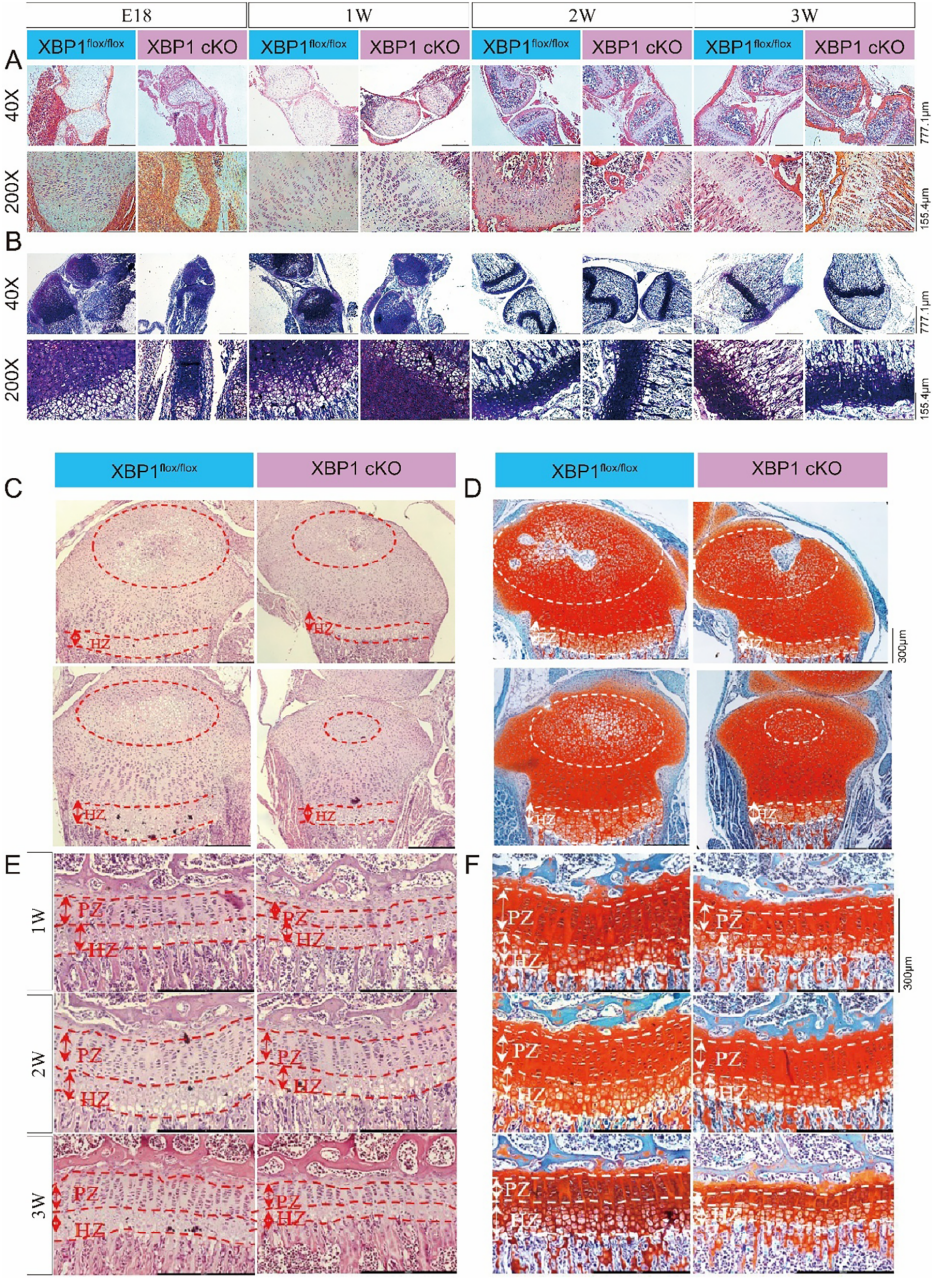
Cartilage‐specific deletion of *Xbp1* impairs growth plate development and delays secondary ossification. (A) Representative hematoxylin and eosin (H&E) staining of proximal tibia sections from *Xbp1* ^flox/flox^ and *Xbp1*cKO mice from embryonic day 18 to 3 weeks after birth. Scale bar: 777.1 or 155.4 µm. *n* = 3–5 mice per genotype per time point. (B) Representative toluidine blue staining of proximal tibia sections from *Xbp1* ^flox/flox^ and *Xbp1*cKO mice from embryonic day 18 to 3 weeks after birth. Scale bar: 777.1 or 155.4 µm. *n* = 3–5 mice per genotype per time point. (C) H&E staining of proximal tibia sections from *Xbp1* ^flox/flox^ and *Xbp1*cKO mice at 1 week after birth at 100× magnification. (D) Safranin fast green staining of proximal tibia sections from *Xbp1* ^flox/flox^ and *Xbp1*cKO mice at 1 week after birth at 100× magnification. (E) H&E staining of the growth plate region of *Xbp1* ^flox/flox^ and *Xbp1*cKO mice at 1 week after birth, magnified 200 times. (F) Safranin fast green staining of the growth plate region of *Xbp1* ^flox/flox^ and *Xbp1*cKO mice at 1 week after birth, magnified 200 times.

Littermate *Xbp1* ^flox/flox^ and *Xbp1*cKO mice were identified by tail genotyping for subsequent experiments (Figure S1A). Immunofluorescence (IF, Figure S1B) and Western blotting (WB, Figure S1C) confirmed efficient deletion of *Xbp1* in primary chondrocytes (Passage 0, P0) from *Xbp1*cKO mice. Compared with *Xbp1* ^flox/flox^ mice, *Xbp1*cKO mice exhibited shorter stature (Figure S1D,E), reduced body length (Figure S1F), and lower body weight (Figure S1G). Then we analysed the growth plates from embryonic day 18 (E18) to postnatal week 3 (3 W). *Xbp1*cKO mice showed decreased *Xbp1*s levels in growth plates from E18 to postnatal 3 W (Figure S1H). Saffron O/Fast Green staining revealed delayed formation of the secondary ossification center and a shortened hypertrophic zone in *Xbp1*cKO mice compared with *Xbp1* ^flox/flox^ mice (Figure S1I). Consistent with these findings, H&E staining (Figure 1A) demonstrated reduced growth plate thickness and impaired development. To further assess growth plate architecture, we examined histological sections at higher magnifications. At 100× (Figure 1B,C), *Xbp1* cKO mice exhibited smaller ossified regions in the femur and tibia (Figure S2A,B). At 200× magnification (Figure 1D,E), the proliferative (PZ), hypertrophic (HZ), and transitional zones (TZ) were all significantly shortened in Xbp1cKO mice at postnatal day 7 (Figure S2C–E).

To further assess the changes in cartilage matrix composition, we performed immunohistochemical (IHC) staining for collagen type II (COL2) and COL10 at three postnatal time points: day 7 (1 W), day 14 (2 W), and day 21 (3 W) (Figure S3). In the PZ, COL2‐positive chondrocytes in *Xbp1* ^flox/flox^ mice showed a progressive increase from 1 to 2 W, reaching a peak at 2 W before slightly declining at 3 W (Figure S3A). In contrast, *Xbp1*cKO mice exhibited significantly lower COL2‐positive cell percentages across all time points, indicating sustained impairment of anabolic matrix synthesis in the absence of functional XBP1s. Conversely, in the HZ, COL10‐positive chondrocytes displayed the opposite pattern (Figure S3B). *Xbp1*cKO mice showed a marked increase in COL10‐positive cells from 1 to 3 W, peaking at 2 W before moderately declining at 3 W. Throughout the observation period, COL10‐positive cell proportions in *Xbp1*cKO mice were consistently and significantly higher than those in *Xbp1* ^flox/flox^ littermates. This premature and excessive hypertrophic marker expression suggests accelerated and potentially dysregulated chondrocyte maturation. Collectively, these temporal and spatial analyses reveal that cartilage‐specific *Xbp1* deletion results in a dual pathological phenotype: impaired anabolic matrix production in the proliferative compartment coupled with precocious hypertrophic differentiation, most pronounced at the critical 2 W developmental window. These matrix compositional defects provide a cellular basis for the observed growth plate shortening and skeletal dysplasia in *Xbp1*cKO mice.

### 3.2 CircTspan3 is Identified in Cartilage, and XBP1s Promotes Its Biogenesis

**FIGURE 2:**
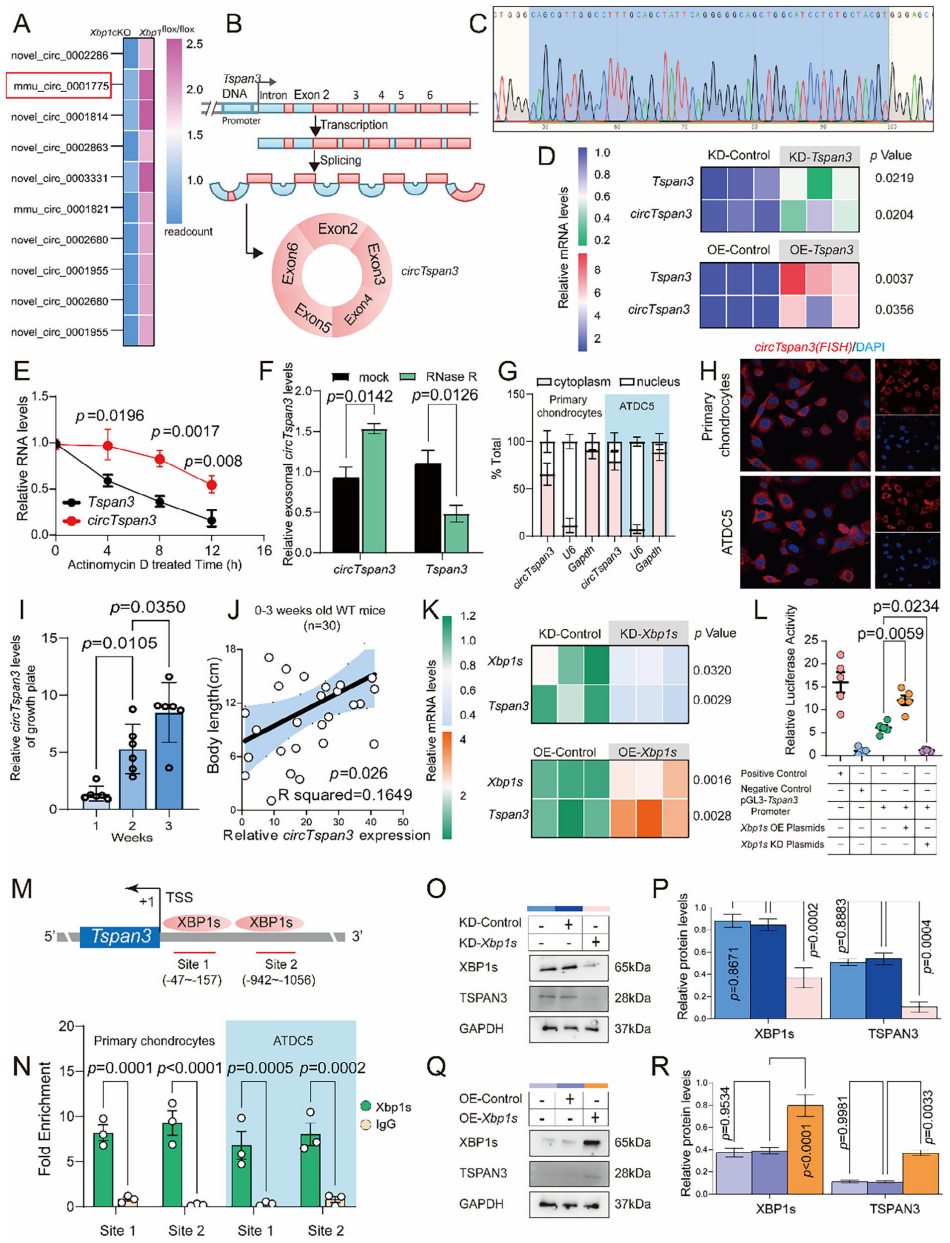
*CircTspan3* is identified in cartilage and is transcriptionally regulated by XBP1s. (A) Heatmap showing differentially circRNAs in knee cartilage from *Xbp1*cKO mice compared to *Xbp1* ^flox/flox^ littermate controls at 4 weeks of age. Color scale indicates log_2_‐transformed normalized read counts (blue: low expression, red: high expression). (B) Schematic diagram showing the genomic structure of the mouse *Tspan3* gene and the back‐splicing event generating *circTspan3*. Diagram created based on circBase annotation (http://www.circbase.org/, ID: mmu_circ_0001775) and UCSC Genome Browser (mm10 assembly). (C) Sanger sequencing validation of the *circTspan3* back‐splice junction (BSJ). Representative result from *n *= 3 independent PCR amplifications. (D) RT‐qPCR quantification of *circTspan3* levels following knockdown (KD) or overexpression (OE) of linear *Tspan3* in primary mouse chondrocytes (Passage 2, P2). Both *circTspan3* and linear *Tspan3* were normalized to *Gapdh*. Data expressed as fold change relative to control groups. Unpaired two‐tailed Student's *t*‐test. *n *= 3 biological replicates per group. Data presented as mean ± SEM. (E) Actinomycin D transcriptional shut‐off assay comparing stability of *circTspan3* versus linear *Tspan3* mRNA. Values at each time point were normalized to *Gapdh* (internal reference gene whose stability is relatively unaffected by actinomycin D over this time course) and expressed as percentage of 0‐h time point (immediately before actinomycin D addition). Paired two‐tailed Student's *t*‐test comparing *circTspan3* vs. *linear Tspan3* at each time point. *n *= 3 biological replicates per time point. Data presented as mean ± SEM. (F) RNase R resistance assay confirming circular structure of *circTspan3*. Paired two‐tailed Student's *t*‐test. *n *= 3 biological replicates. Data presented as mean ± SEM. (G) RT‐qPCR analysis of *circTspan3* subcellular localization in primary mouse chondrocytes (P2) and ATDC5 cells following nuclear–cytoplasmic fractionation. *n *= 3 biological replicates per cell type. Data presented as mean ± SEM. (H) Representative fluorescence in situ hybridization (FISH) images showing *circTspan3* (red) subcellular localization in primary mouse chondrocytes (P0, Top) and ATDC5 cells (P5, Bottom). Scale bar: 20 µm. Representative images from *n *= 3 independent experiments, with >50 cells examined per experiment, showing a consistent localization pattern. (I) Developmental expression profile of *circTspan3* in mouse tibial growth plate cartilage from postnatal week 1 (1 W) to week 3 (3 W). *CircTspan3* was quantified by RT‐qPCR using divergent primers, normalized to *Gapdh*, and expressed as fold change relative to 1 W. One‐way ANOVA followed by Tukey's post hoc test. *n *= 6 mice per time point. Data presented as mean ± SEM. (J) Pearson correlation analysis between *circTspan3* expression levels and body length in wild‐type C57BL/6 mice during postnatal development. *CircTspan3* expression in growth plate cartilage was quantified by RT‐qPCR as in panel I. Body length was measured from the nose tip to the tail base using a ruler with 1 mm precision. Each data point represents one individual mouse (*n *= 24 total, pooled from 1 W, 2 W, and 3 W time points with *n *= 8 per time point). (K) RT‐qPCR quantification of linear *Tspan3* mRNA and *circTspan3* levels following *XBP1s* modulation in ATDC5 cells. *Xbp1s*, linear *Tspan3*, and *circTspan3* were quantified by RT‐qPCR and normalized to *Gapdh*. Unpaired two‐tailed Student's *t*‐test. *n *= 3 biological replicates per condition. Data presented as mean ± SEM. (L) Dual‐luciferase reporter assay demonstrating XBP1s‐dependent transcriptional activation of *Tspan3* promoter. Firefly luciferase activity was normalized to Renilla luciferase activity (internal control), and fold induction was calculated relative to Vector control. One‐way ANOVA followed by Tukey's post hoc test, *n *= 3 biological replicates performed in technical triplicates. Data presented as mean ± SEM. (M) Bioinformatic prediction of XBP1s‐binding motifs in the mouse *Tspan3* promoter region using AnimalTFDB3.0 database (http://bioinfo.life.hust.edu.cn/AnimalTFDB/). (N) Chromatin immunoprecipitation followed by quantitative PCR (ChIP‐qPCR) validation of XBP1s binding to *Tspan3* promoter in ATDC5 cells (P7). Enrichment was calculated as a percentage of input. Unpaired two‐tailed Student's *t*‐test. *n *= 3 biological replicates. Data presented as mean ± SEM. (O)Western blot analysis of XBP1s and TSPAN3 protein levels following *Xbp1* knockdown in primary mouse chondrocytes (P2). Representative blots from *n *= 3 independent experiments. (P) Densitometry quantification of Western blots from panel O. XBP1s and TSPAN3 band intensities were quantified using ImageJ, normalized to GAPDH. One‐way ANOVA followed by Tukey's post hoc test. Data presented as mean ± SEM. (Q) Western blot analysis of XBP1s and TSPAN3 protein levels following XBP1s overexpression in primary mouse chondrocytes (P2). Total protein lysates (30 µg per lane) were analyzed as described in panel O. Representative blots from *n *= 3 independent experiments. (R) Densitometry quantification of Western blots from panel Q. One‐way ANOVA followed by Tukey's post hoc test. *n *= 3 biological replicates. Data presented as mean ± SEM. Statistical analyses were conducted using GraphPad Prism 10.0. Statistical significance: *p* < 0.05, not significant: *p *≥ 0.05.

High‐throughput sequencing was used to profile circRNA expression in mouse cartilage. Based on four systematic criteria (statistical significance, host gene function, expression abundance, and correlation with anabolic markers, as detailed in Section 2.3 of the Methods), *circTspan3* (mmu_circ_0001775) was prioritised as the primary validation target (Figure 2A). Both *Xbp1s* and *circTspan3* were reduced in primary chondrocytes (P0) from Xbp1cKO mice compared with littermate controls (Figure S4A). Moreover, their expression levels were positively correlated in cartilage samples (Figure S4B). According to circBase, *circTspan3* is generated by back‐splicing of exons 2–6 of Tspan3 (606 bp) on chromosome 9 (Figure 2B). The head‐to‐tail junction was confirmed by Sanger sequencing (Figure 2C). Knockdown (KD) or overexpression (OE) of *Tspan3* in primary chondrocytes (P2) altered *circTspan3* abundance proportionally, indicating that the linear precursor lies upstream of the circular isoform (Figure 2D). Actinomycin‐D chase assays demonstrated that *circTspan3* is resistant to transcriptional shutdown, whereas linear *Tspan3* mRNA decays rapidly (Figure 2E). Consistently, RNase R digestion preferentially degraded linear transcripts, confirming the higher stability of *circTspan3* (Figure 2F). FISH and nuclear—cytoplasmic fractionation revealed that *circTspan3* is predominantly cytoplasmic in *Xbp1* ^flox/flox^ cartilage, whereas its overall abundance is reduced in *Xbp1*cKO mice (Figure S5A–C). In wild‐type primary chondrocytes (P2) and ATDC5 (P5) cells, *circTspan3* likewise localized mainly to the cytoplasm (Figure 2G,H). Within the growth plate of wild‐type mice, *circTspan3* levels rose between postnatal weeks 1 and 3 (Figure 2I) and correlated positively with body length (Figure 2J; *p* = 0.026), suggesting a role in longitudinal growth.

Modulating *Xbp1s* in ATDC5 cells affected both *Tspan3* mRNA and *circTspan3* levels in parallel (Figure 2K). Luciferase assays showed that XBP1s activate the *Tspan3* promoter, whereas *Xbp1s* knockdown abolishes this effect (Figure 2L). AnimalTFDB3.0 predicted two XBP1s‐binding motifs within the promoter (Figure 2M). ChIP‐qPCR confirmed XBP1s occupancy at these sites, enhancing transcription (Figure 2N). Correspondingly, TSPAN3 protein levels tracked with XBP1s expression (Figure 2O,R). Collectively, these data establish *Tspan3* as a direct XBP1s target, with XBP1s—dependent transcription driving the production of both linear and circular *Tspan3* transcripts.

### 3.3 CircTspan3 is the Primary Driver of Sustained Anabolic Gene Expression Independent of Linear Transcripts

To distinguish linear versus circular *Tspan3* contributions, we performed dual‐timepoint pulse‐chase experiments (Figure S6). Tunicamycin pulse induced co‐elevation of linear *Tspan3* and *circTspan3* in G3 at 2.5 h. Actinomycin D treatment (G4) achieved comparable *circTspan3* despite linear transcript reduction.

By 9 h, linear *Tspan3* declined dramatically, while *circTspan3* remained stable. Anabolic genes diverged: G3 showed a non‐significant decline, whereas G4 demonstrated a significant enhancement. At 9 h, despite equivalent *circTspan3*, G4 exhibited higher anabolic expression than G3, establishing *circTspan3*'s independent role through time‐dependent cumulative effects.

### 3.4 CircTspan3 Enhances Cartilage Anabolism by Inhibiting Apoptosis and Ferroptosis

**FIGURE 3:**
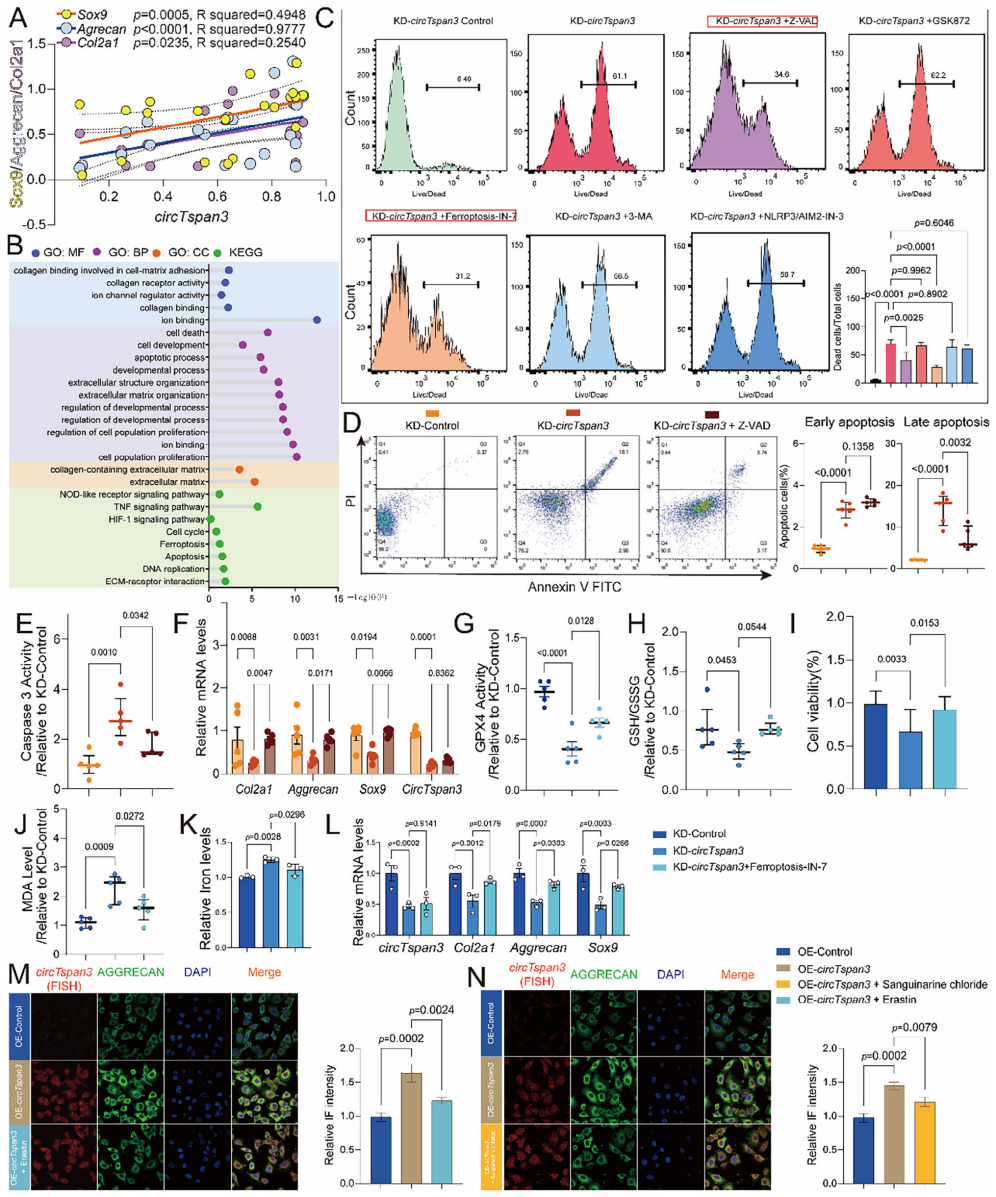
*CircTspan3* promotes cartilage anabolism and chondrocyte survival by inhibiting apoptosis and ferroptosis. (A) Pearson correlation analysis between *circTspan3* expression and cartilage anabolic markers (*Sox9, Col2a1*, *Aggrecan*) in mouse knee cartilage samples. Total *n *= 40 samples. Pearson correlation coefficients (r) and two‐tailed *p* values are indicated. (B) Gene Ontology (GO) and Kyoto Encyclopedia of Genes and Genomes (KEGG) pathway enrichment analysis of differentially expressed genes following *circTspan3* knockdown in ATDC5 cells (Passage 7, P7). (C) Flow cytometry analysis of cell death following *circTspan3* knockdown with or without cell death pathway inhibitors. One‐way ANOVA followed by Dunnett's post hoc test. Data presented as mean ± SEM. (D–F) ADTC5 cells (P7) were divided into three groups: KD‐Control, KD‐*circTspan3*, and KD‐*circTspan3* + Z‐VAD. (D) Representative flow cytometry plots and quantification of apoptotic cells using Annexin V‐FITC/PI staining. One‐way ANOVA followed by Tukey's post hoc test. *n *= 5 biological replicates. Data presented as mean ± SEM. (E) Caspase‐3 enzymatic activity assay in ATDC5 cells (P7) following *circTspan3* knockdown with or without Z‐VAD treatment. Caspase‐3 activity was calculated as relative fluorescence units (RFU) per mg protein and expressed as fold change relative to control. One‐way ANOVA followed by Tukey's post hoc test. *n* = 5 biological replicates. Data presented as mean ± SEM. (F) RT‐qPCR analysis of cartilage anabolic markers (*Col2a1*, *Aggrecan*, *Sox9*) following *circTspan3* knockdown with or without Z‐VAD treatment. Two‐way ANOVA (treatment × gene) followed by Tukey's post hoc test. *n* = 5 biological replicates. Data presented as mean ± SEM. (G–L) ADTC5 cells (P7) were assigned to KD‐Control, KD‐*circTspan3*, and KD‐*circTspan3* + Ferroptosis‐IN‐7 groups. (G) Glutathione peroxidase 4 (GPX4) enzymatic activity assay following *circTspan3* knockdown with or without Ferroptosis‐IN‐7 treatment. GPX4 activity calculated as nmol NADPH oxidized per minute per mg protein and expressed as a fold change relative to control. One‐way ANOVA followed by Tukey's post hoc test. *n* = 5 biological replicates. Data presented as mean ± SEM. (H) Reduced glutathione to oxidized glutathione ratio (GSH/GSSG) measurement using GSH and GSSG Assay Kit. GSH/GSSG ratio expressed as an absolute ratio and expressed as a fold change relative to control. One‐way ANOVA followed by Tukey's post hoc test. *n* = 5 biological replicates. Data presented as mean ± SEM. (I) Cell viability assessed by CCK‐8 assay and calculated as a percentage relative to control. One‐way ANOVA followed by Tukey's post hoc test. *n* = 5 biological replicates. Data presented as mean ± SEM. (J) Lipid peroxidation measured by malondialdehyde (MDA) assay. MDA concentration calculated from the standard curve and percentage relative to the control. One‐way ANOVA followed by Tukey's post hoc test. *n* = 5 biological replicates. Data presented as mean ± SEM. (K) Intracellular ferrous iron (Fe^2^⁺) measurement using FerroOrange probe. Cells were analyzed by flow cytometry. Mean fluorescence intensity (MFI) quantified using FlowJo software and expressed as a fold change relative to control. One‐way ANOVA followed by Tukey's post hoc test. *n* = 5 biological replicates. Data presented as mean ± SEM. (L) RT‐qPCR analysis of cartilage anabolic markers following *circTspan3* knockdown with or without Ferroptosis‐IN‐7 treatment. RNA extraction and qPCR performed as in panel F. Two‐way ANOVA followed by Tukey's post hoc test. *n* = 3 biological replicates. Data presented as mean ± SEM. (M) Immunofluorescence staining and quantification of AGGRECAN protein expression following *circTspan3* overexpression with or without Erastin. Scale bar: 50 µm. Representative images from *n* = 3 independent experiments. The right panel shows quantification of AGGRECAN fluorescence intensity measured using ImageJ. Integrated fluorescence intensity was normalized to cell area and expressed as a fold change relative to Vector + Vehicle. One‐way ANOVA followed by Tukey's post hoc test. *n* = 3 biological replicates (50+ cells analyzed per replicate). Data presented as mean ± SEM. (N) Immunofluorescence staining and quantification of AGGRECAN protein expression following *circTspan3* overexpression with or without Sanguinarine chloride. Scale bar: 50 µm. Representative images from *n* = 3 independent experiments. The right panel shows quantification of AGGRECAN fluorescence intensity measured using ImageJ. Integrated fluorescence intensity was normalized to cell area and expressed as a fold change relative to Vector + Vehicle. One‐way ANOVA followed by Tukey's post hoc test. *n* = 3 biological replicates (50+ cells analyzed per replicate). Data presented as mean ± SEM. Statistical analyses were performed using GraphPad Prism 10.0.

Pearson correlation analysis revealed that *Xbp1s* expression correlated positively with anabolic markers (*Sox9*, *Col2a1*, *Aggrecan*) but not with catabolic markers (*Mmp13*, *Adamts4*, *Adamts5*) (Figure S6). Similarly, *circTspan3* expression correlated positively with anabolic markers (Figure 3A), whereas no significant correlation was observed with catabolic genes (Figure S8). Longitudinal expression analysis from embryonic day 18 (E18) to postnatal week 3 (3 W) showed that *Xbp1*cKO mice exhibited consistently lower levels of *circTspan*3, *Col2a1*, *Aggrecan*, and *Sox9* in knee cartilage relative to controls, despite the expected age‐related increase in these anabolic factors (Figure S9).

To elucidate the molecular mechanisms by which *circTspan3* regulates chondrocyte function, we performed transcriptomic profiling (RNA‐seq) after adenoviral‐mediated knockdown of *circTspan3* in ATDC5 cells (P7, KD‐*circTspan3*). Gene Ontology (GO) enrichment analysis indicated that differentially expressed genes were significantly associated with collagen biosynthesis and tissue growth. In parallel, Kyoto Encyclopedia of Genes and Genomes (KEGG) pathway analysis highlighted enrichment in apoptosis—and ferroptosis—related pathways (Figure 3B).

To functionally validate these transcriptomic findings, *circTspan3*‐deficient ATDC5 cells (P7) were treated with inhibitors targeting distinct cell death pathways (Figure 3C). Flow cytometry demonstrated that *circTspan3* silencing markedly increased cell death, which was significantly rescued by the apoptosis inhibitor Z‐VAD or the ferroptosis inhibitor Ferroptosis‐IN‐7, suggesting that *circTspan3* promotes chondrocyte survival by suppressing apoptosis and ferroptosis.

We next examined whether these inhibitors could restore cartilage anabolic activity following *circTspan3* depletion. Treatment with Z‐VAD reduced late chondrocyte apoptosis (Figure 3D), inhibited caspase‐3 activation (Figure 3E), and upregulated anabolic gene expression (Figure 3F). Similarly, ferroptosis induced by *circTspan3* knockdown—evidenced by decreased GPX4 activity, reduced GSH/GSSG ratio, diminished cell viability, and elevated MDA and ferrous iron levels—was attenuated by Ferroptosis‐IN‐7 (Figure 3G–K), concomitant with recovery of cartilage anabolic markers (Figure 3L).

Consistent with these findings, overexpression of *circTspan3* enhanced AGGRECAN expression, whereas treatment with the ferroptosis inducer Erastin or the apoptosis inducer Sanguinarine chloride suppressed AGGRECAN levels (Figure 3M,N). Together, these findings indicate that *circTspan3* enhances cartilage anabolism by inhibiting apoptosis and ferroptosis, thereby contributing to cartilage growth and homeostasis.

### 3.5 Exosomal circTspan3 Promotes Cartilage Anabolism and Suppresses Chondrocyte Apoptosis and Ferroptosis

**FIGURE 4:**
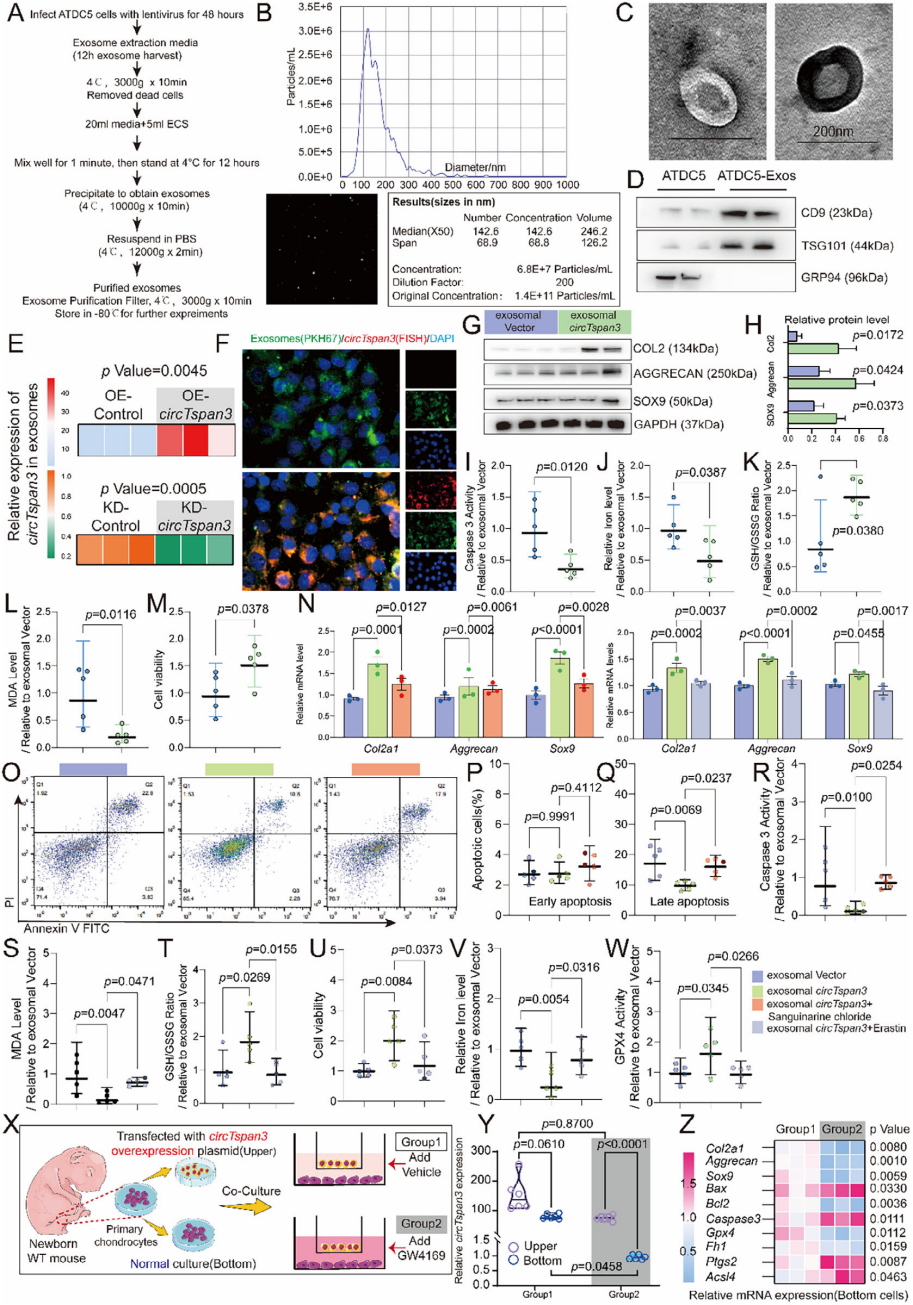
Exosomal *circTspan3* mediates paracrine signaling to promote cartilage anabolism and suppress chondrocyte apoptosis and ferroptosis. (A) Schematic illustration of the experimental workflow for exosome isolation, characterization, and functional testing. Donor ATDC5 cells (Passage 8‐11, P8‐11) were transfected with *circTspan3*‐overexpressing plasmid (OE‐*circTspan3*) or *circTspan3* siRNA (KD‐*circTspan3*) for 48 h. Culture supernatants were collected, and exosomes were isolated by ultracentrifugation. Purified exosomes were then applied to recipient naïve ATDC5 chondrocytes (P8‐11) cultured in separate dishes. (B) Nanoparticle tracking analysis (NTA) showing the size distribution and concentration of exosomes isolated from donor ATDC5 cells (P8‐11). Representative histogram from *n* = 3 independent exosome preparations. (C) Transmission electron microscopy (TEM) images displaying the morphology of purified exosomes. Scale bar: 200 nm. Representative images from *n* = 3 independent exosome preparations. (D) Western blot analysis of exosomal markers CD9 and TSG101, and the absence of endoplasmic reticulum marker GRP94 in isolated exosomes. (E) RT‐qPCR quantification of *circTspan3* levels in exosomes derived from donor ATDC5 cells (P8‐P11) with *circTspan3* overexpression (*circTspan3* OE) or knockdown (KD‐*circTspan3*). Data are normalized to the OE‐Control group and presented as mean ± SEM (*n* = 3 biological replicates). Statistical analysis was performed using one‐way ANOVA followed by Tukey's post hoc test. (F) Representative confocal microscopy images showing uptake of PKH67‐labeled exosomes (green) by recipient ATDC5 cells (P8‐11) at 1 h post‐treatment. *CircTspan3* was detected by FISH using Cy3‐labeled probe (red). Nuclei were counterstained with DAPI (blue). Scale bar: 20 µm. Representative images from *n* = 3 independent experiments. (G) Western blot analysis was used to detect the effects of exosomal *circTspan3* on cartilage synthesis‐related indicators in recipient cells (P8‐11) treated with exosomes (3 × 10^8^ particles/mL) for 48 h. (H) Densitometric quantification of Western blot bands shown in (G). Data are normalized to the Vector exosome‐treated group and presented as mean ± SEM (*n* = 3 biological replicates). Statistical comparisons were performed using one‐way ANOVA followed by Tukey's post hoc test. (I) Caspase‐3 activity assay in recipient ATDC5 cells (P8‐11) treated with exosomes (3 × 10^8^ particles/mL) for 48 h. Caspase‐3 activity was measured as relative fluorescence units (RFU) per mg protein and expressed as fold change relative to the Vector exosome group. Data are presented as mean ± SEM. Statistical analysis: one‐way ANOVA followed by Tukey's post hoc test. *n* = 5 biological replicates. *p* values are indicated in the figure. (J) Intracellular ferrous iron (Fe^2^⁺) measurement in recipient ATDC5 cells (P8‐11) treated with exosomes (3 × 10^8^ particles/mL) for 48 h using FerroOrange probe. Mean fluorescence intensity (MFI) was quantified by flow cytometry and expressed as a fold change relative to the Vector exosome group. Data are presented as mean ± SEM. Statistical analysis: one‐way ANOVA followed by Tukey's post hoc test. *n* = 5 biological replicates. *p* values indicated in the figure. (K) Reduced glutathione to oxidized glutathione ratio (GSH/GSSG) measurement in recipient ATDC5 cells (P8‐11) treated with exosomes (3 × 10^8^ particles/mL) for 48 h. GSH/GSSG ratio was expressed as a fold change relative to the Vector exosome group. Data are presented as mean ± SEM. Statistical analysis: one‐way ANOVA followed by Tukey's post hoc test. *n* = 5 biological replicates. *p* values indicated in the figure. (L) Malondialdehyde (MDA) assay measuring lipid peroxidation levels in recipient ATDC5 cells (P8‐11) treated with exosomes (3 × 10^8^ particles/mL) for 48 h. MDA concentration was expressed as a fold change relative to the Vector exosome group. Data are presented as mean ± SEM. Statistical analysis: one‐way ANOVA followed by Tukey's post hoc test. *n* = 5 biological replicates. *p* values indicated in the figure. (M) Cell viability assessment by CCK‐8 assay in recipient ATDC5 cells (P8‐11) treated with exosomes (3 × 10^8^ particles/mL) for 48 h. Absorbance at 450 nm was measured and expressed as a fold change relative to the Vector exosome group. Data are presented as mean ± SEM. Statistical analysis: one‐way ANOVA followed by Tukey's post hoc test. *n* = 5 biological replicates. *p* values indicated in the figure. (N) RT‐qPCR analysis of cartilage anabolic markers (*Col2a1*, *Aggrecan*, *Sox9*) in recipient ATDC5 cells (P8‐11) treated with exosomal *circTspan3* (3 × 10^8^ particles/mL) combined with apoptosis agonist Sanguinarine chloride (0.5 *p*) or ferroptosis agonist Erastin (10 µm) for 48 h. Gene expression was normalized to *Gapdh* and expressed as fold change relative to the exosomal Vector group. Data are presented as mean ± SEM. Statistical analysis: two‐way ANOVA (exosome treatment × agonist) followed by Tukey's post hoc test. *n* = 5 biological replicates. *p* values indicated in the figure. (O) Flow cytometry analysis of apoptotic cells using Annexin V‐FITC/PI staining in recipient ATDC5 cells (P8‐11) treated with exosomal *circTspan3* (3 × 10⁸ particles/mL) combined with Sanguinarine chloride (0.5 µm) for 48 h. Representative flow cytometry plots from *n* = 5 independent experiments. (P) Quantification of early apoptotic cell percentage from panel O. Data are presented as mean ± SEM (apoptotic percentage is not normalized; raw percentages reported). Statistical analysis: one‐way ANOVA followed by Tukey's post hoc test. *n* = 5 biological replicates. *p* values indicated in the figure. (Q) Quantification of late apoptotic cell percentage from panel O. Data are presented as mean ± SEM (apoptotic percentage is not normalized; raw percentages reported). Statistical analysis: one‐way ANOVA followed by Tukey's post hoc test. *n* = 5 biological replicates. *p* values indicated in the figure. (R) Caspase‐3 enzymatic activity assay in recipient ATDC5 cells (P8‐11) treated with exosomal *circTspan3* (3 × 10^8^ particles/mL) combined with Sanguinarine chloride (0.5 µm) for 48 h. Caspase‐3 activity was expressed as fold change relative to the exosomal Vector group. Data are presented as mean ± SEM. Statistical analysis: one‐way ANOVA followed by Tukey's post hoc test. *n* =5 biological replicates. *p* values indicated in the figure. (S) MDA assay measuring lipid peroxidation in recipient ATDC5 cells (P8‐11) treated with exosomal *circTspan3* (3 × 10^8^ particles/mL) combined with Erastin (10 µm) for 48 h. MDA level was expressed as a fold change relative to the exosomal Vector group. Data are presented as mean ± SEM. Statistical analysis: one‐way ANOVA followed by Tukey's post hoc test. *n* = 5 biological replicates. *p* values indicated in the figure. (T) GSH/GSSG ratio measurement in recipient ATDC5 cells (P8‐11) treated with exosomal circTspan3 (3 × 10^8^ particles/mL) combined with Erastin (10 µm) for 48 h. Ratio was expressed as fold change relative to exosomal Vector group. Data are presented as mean ± SEM. Statistical analysis: one‐way ANOVA followed by Tukey's post hoc test. *n* = 3 biological replicates. *p* values indicated in the figure. (U) Cell viability assessment by CCK‐8 assay in recipient ATDC5 cells (P8‐11) treated with exosomal circTspan3 (3 × 10^8^ particles/mL) combined with Erastin (10 µm) for 48 h. Viability was expressed as a fold change relative to the exosomal Vector group. Data are presented as mean ± SEM. Statistical analysis: one‐way ANOVA followed by Tukey's post hoc test. *n* = 5 biological replicates. *p* values indicated in the figure. (V) Intracellular Fe^2^⁺ measurement using FerroOrange probe in recipient ATDC5 cells (P8‐11) treated with exosomal circTspan3 (3 × 10^8^ particles/mL) combined with Erastin (10 µm) for 48 h. MFI was quantified by flow cytometry and expressed as fold change relative to exosomal Vector group. Data are presented as mean ± SEM. Statistical analysis: one‐way ANOVA followed by Tukey's post hoc test. *n* = 5 biological replicates. *p* values indicated in the figure. (W) Glutathione peroxidase 4 (GPX4) enzymatic activity assay in recipient ATDC5 cells (P8‐11) treated with exosomal *circTspan3* (3 × 10^8^ particles/mL) combined with Erastin (10 µm) for 48 h. GPX4 activity was expressed as fold change relative to the exosomal Vector group. Data are presented as mean ± SEM. Statistical analysis: one‐way ANOVA followed by Tukey's post hoc test. *n* = 5 biological replicates. *p* values indicated in the figure. (X) Schematic diagram of the Transwell co‐culture system. Donor ATDC5 cells (P8‐11) overexpressing *circTspan3* were seeded in the upper chamber, while naïve wild‐type recipient ATDC5 cells (P8‐11) were seeded in the lower chamber. Chambers were separated by a 0.4‐µm pore membrane that permits exosome passage but prevents cell migration. The exosome secretion inhibitor GW4869 (10 µm) was added to selected wells to block exosome release from donor cells (P8‐11). (Y) RT‐qPCR quantification of *circTspan3* levels in donor cells (upper chamber) and recipient cells (lower chamber) after 48‐h co‐culture with or without GW4869 treatment. *CircTspan3* expression was normalized to *Gapdh* and expressed as fold change relative to the corresponding chamber of the control group without GW4869. Data are presented as mean ± SEM. Statistical analysis: two‐way ANOVA (chamber location × GW4869 treatment) followed by Tukey's post hoc test. *n* = 3 biological replicates. *p* values indicated in the figure. (Z) RT‐qPCR analysis of cartilage anabolic markers (*Col2a1*, *Aggrecan*, *Sox9*), apoptosis marker (*Bax*, *Caspase3*, and *Bcl2*), and ferroptosis markers (*Gpx4*, *Fh1*, *Ptgs2*, *Acsl4)* in recipient cells (lower chamber) after 48‐h co‐culture with or without GW4869 treatment. Gene expression was normalized to *Gapdh* and expressed as fold change relative to the control group without GW4869 (Group 1). Data are presented as mean ± SEM. Statistical analysis: unpaired two‐tailed Student's *t*‐test for each gene. *n* = 3 biological replicates. *p* values indicated in the figure. Copyright attribution: Schematic illustrations in panels X were created using graphical elements adapted from Servier Medical Art (https://smart.servier.com/) by Servier, licensed under a Creative Commons Attribution 3.0 Unported License (CC BY 3.0).

*CircTspan3* was detectable in EVs isolated from the peripheral blood of *Xbp1* ^flox/flox^ mice (Figure S10); however, the in vivo tissue source of this circulating pool remains unresolved. To test whether *circTspan3* is exosome‐encapsulated and mediates intercellular (paracrine) signaling, we isolated exosomes from donor ATDC5 cultures (Passage 8‐11, P8‐11) overexpressing or silencing *circTspan3* (Figure 4A–E), and applied the purified vesicles to independent, naïve recipient chondrocytes maintained in separate dishes. NTA and TEM confirmed particle size and morphology, and WB verified CD9/TSG101 with the absence of GRP94 (Figure 4B–D). In recipient ADTC5 cells (P8‐11), *circTspan3* increased in a time‐dependent manner, peaking at 6 h (Figure S11). Consistently, PKH67 tracking combined with FISH demonstrated rapid uptake of donor‐derived exosomes by recipients within 1 h (Figure 4F).

To determine the optimal chondroprotective dose of *circTspan3*‐enriched exosomes.

We performed a 48‐h titration experiment using *circTspan3*‐enriched exosomes at three defined particle concentrations (1 × 10^8^, 3 × 10^8^, and 1 × 10^9^ particles/mL). The exosomes were produced as a single batch, with comparable particle size distribution and uniform *circTspan3* payload content confirmed by NTA and RT‐qPCR (Figure S12A). Functional assays demonstrated a clear particle‐defined dose–response relationship (Figure S12B–E). *Col2a1* expression increased with dose and reached a maximum at 3 × 10^8^ particles/mL, then plateaued at 1 × 10⁹ particles/mL. Apoptosis, assessed by Annexin‐V/PI staining, was most strongly reduced at the intermediate dose, while ferroptosis inhibition (GPX4 activity) also peaked at the same level. Together, these results indicate that the intermediate dose (3 × 10^8^ particles/mL) provides the optimal balance between anabolic activation and suppression of apoptosis and ferroptosis, thereby serving as the effective dosing reference for subsequent experiments.

Functionally, treatment with exosomal *circTspan3* (3 × 10^8^ particles/mL) increased anabolic protein markers in recipient chondrocytes (P8‐11, Figure 4G,H), as also indicated by Caspase‐3 activity (Figure 4I) and TUNEL staining (Figure S13). Similarly, exosomal *circTspan3* treatment reduced MDA level, GSH/GSSG ratio, suppressed cell viability, reduced intracellular iron levels, and GPX4 activity (Figure 4J–M).

Next, we administered Sanguinarine chloride and Erastin, apoptosis and ferroptosis agonists, alongside exosomal *circTspan3* treatment. Both agonists partially reversed the ability of exosomal *circTspan3* to promote cartilage anabolism (Figure 4N), inhibit apoptosis (Figure 4O–R), and suppress ferroptosis (Figure 4S–W). To directly test whether intercellular transfer of *circTspan3* via exosomes is required, we used a transwell system in which donor chondrocytes (P8‐11) overexpressing *circTspan3* were physically separated from recipient naïve chondrocytes (P8‐11) by a permeable membrane that precludes cell–cell contact. The exosome‐release inhibitor GW4869 was added to selected groups (Figure 4X). While *circTspan3* remained comparably high in donors across groups, its level was significantly reduced in recipients when GW4869 was present, indicating impaired vesicular transfer (Figure 4Y). Functionally, GW4869 blunted anabolic marker induction (*Col2a1*, *Aggrecan*, *Sox9*) and increased apoptosis/ferroptosis readouts in recipients (Figure 4Z; Figure S14 and S15). Because donors and recipients were separated and shared only conditioned medium across the membrane, these data constitute rigorous evidence of exosome‐mediated, paracrine communication rather than autocrine self‐stimulation.

### 3.6 Both circTspan3 and Exosomal circTspan3 Promote Cartilage Growth

**FIGURE 5:**
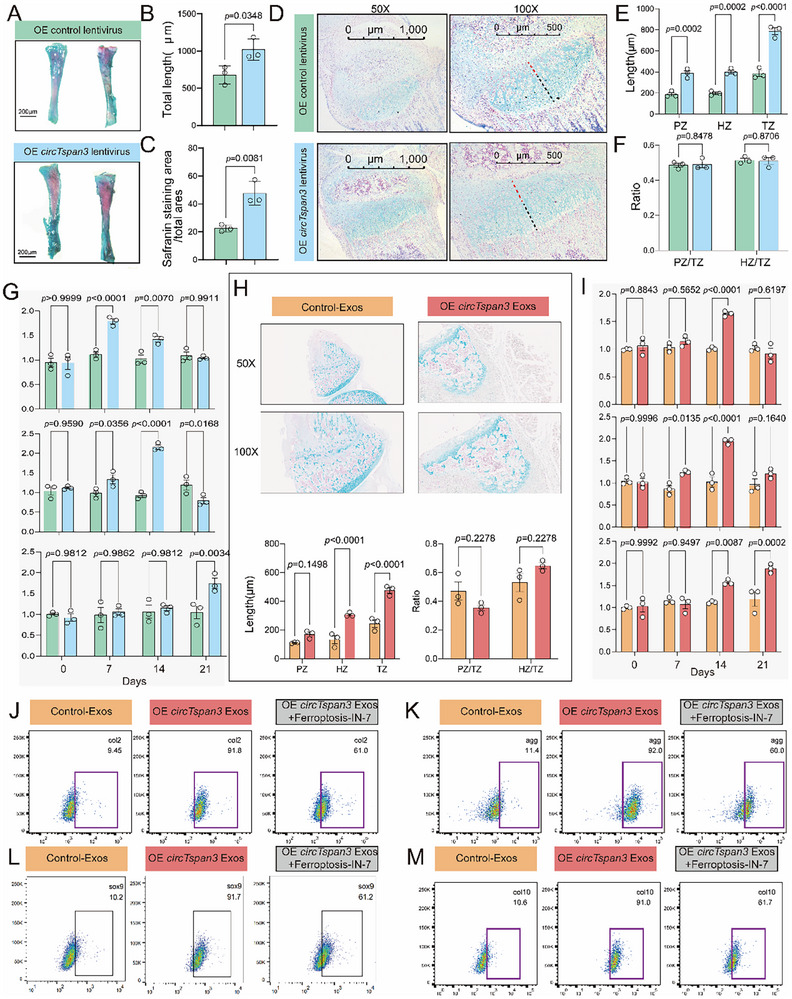
Both *circTspan3* and exosomal *circTspan3* promote growth plate development and chondrogenic differentiation. (A) Representative images of Safranin O/Fast Green staining of tibial growth plate from *Xbp1*cKO mice treated with *circTspan3*‐overexpressing lentivirus (1×10^12^ TU/mL) or control vector for 14 days. Quantification of safranin‐stained area. Data are presented as mean ± SEM (*n* = 3 biologically independent samples per group). (B) Quantification of total tibial length in *Xbp1*cKO cartilage explants treated with *circTspan3*‐overexpressing lentivirus (1×10^12^ TU/mL) or control lentivirus for 14 days *ex vivo*. Tibial length was measured from the proximal to the distal end. Data are presented as mean ± SEM. Statistical analysis: paired two‐tailed Student's *t*‐test. *n* = 3 biologically independent explants per group. *p* values indicated in the figure. (C) Quantification of growth plate area in *Xbp1*cKO cartilage explants treated with *circTspan3*‐overexpressing lentivirus (1 × 10^12^ TU/mL) or control lentivirus for 14 days *ex vivo*. Growth plate area was measured from histological sections. Data are presented as mean ± SEM. Statistical analysis: paired two‐tailed Student's *t*‐test. *n* = 3 biologically independent explants per group. *p* values indicated in the figure. (D) Representative images of Alcian blue/nuclear fast red staining of knee cartilage explants from *Xbp1* cKO mice treated with OE *circTspan3* lentivirus after 14 days ex vivo. Representative images from *n* = 3 biologically independent explants per group. (E) Quantification of proliferative zone (PZ), hypertrophic zone (HZ), and transition zone (TZ) lengths from panel D. Zone lengths were measured from histological sections. Data are presented as mean ± SEM. Statistical analysis: paired two‐tailed Student's *t*‐test for each zone. *n* = 3 biologically independent explants per group. *p* values indicated in the figure. (F) Quantification of PZ/TZ and HZ/TZ ratios from panel D. Ratios were calculated from the zone length measurements in panel E. Data are presented as mean ± SEM. Statistical analysis: paired two‐tailed Student's *t*‐test for each ratio. *n* = 3 biologically independent explants per group. *p* values indicated in the figure. (G) RT‐qPCR time‐course analysis of chondrogenic differentiation markers (*Col2a1(Top)*, *Aggrecan(Middle)*, *Col10a1(Bottom)*) in ATDC5 cells (P5‐7) infected with *circTspan3*‐overexpressing lentivirus (1×10^8^ TU/mL) or control lentivirus and cultured in chondrogenic differentiation medium for 0, 7, 14, and 21 days. Gene expression was normalized to *Gapdh* and expressed as fold change relative to control lentivirus at day 0. Data are presented as mean ± SEM. Statistical analysis: two‐way ANOVA (lentivirus treatment × time point) followed by Tukey's post hoc test for each gene. *n* = 3 biological replicates per time point. *p* values indicated in the figure. (H) Representative images and quantification of Alcian blue/nuclear fast red staining of knee cartilage explants from *Xbp1* cKO mice treated with exosomal *circTspan3*. Data are presented as mean ± SEM. Statistical analysis: paired two‐tailed Student's *t*‐test for each parameter. *n* = 3 biologically independent explants per group. *p* values indicated in the figure. (I) RT‐qPCR time‐course analysis of chondrogenic differentiation markers (*Col2a1(Top)*, *Aggrecan(Middle), Col10a1(Bottom)*) in ATDC5 cells (P5‐7) treated with exosomal *circTspan3* (3 × 10^8^ particles/mL, added at day 0) or Vector exosomes and cultured in chondrogenic differentiation medium for 0, 7, 14, and 21 days. Gene expression was normalized to *Gapdh* and expressed as fold change relative to Vector exosome treatment at day 0. Data are presented as mean ± SEM. Statistical analysis: two‐way ANOVA (exosome treatment × time point) followed by Tukey's post hoc test for each gene. *n* = 3 biological replicates per time point. *p* values indicated in the figure. Flow cytometry analysis of COL2‐positive(J), AGGRECAN‐positive(K), SOX9‐positive(L), COL10‐positive (M) chondrocytes following 21‐day chondrogenic differentiation of ATDC5 cells (P5‐7) treated with exosomal *circTspan3* (3 × 10^8^ particles/mL) combined with Ferroptosis‐IN‐7. Cells were fixed, permeabilized, and stained with anti‐COL2, AGGRECAN, SOX9, or COL10 antibody, followed by fluorescent secondary antibody. *n* = 3 biological replicates.

To evaluate the role of *circTspan3* in growth plate development and cartilage formation, we conducted histological analyses of knee cartilage explants treated with *circTspan3*‐overexpressing lentivirus (1 × 10^12^ TU/mL) for 14 days. Safranin O/Fast Green staining revealed enhanced tibial growth plate development in *Xbp1*cKO mice, as evidenced by increased safranin‐stained area (Figure 5A). *CircTspan3* overexpression significantly extended tibial length and expanded the growth plate (Figure 5B,C). Alcian Blue/Nuclear Fast Red staining further showed increased lengths of the PZ, HZ, and TZ, without altering the PZ/TZ or HZ/TZ ratios (Figure 5D–F). In vitro chondrocyte differentiation assays demonstrated that both *circTspan3*‐overexpressing lentivirus and exosomal *circTspan3* significantly upregulated expression of differentiation markers *Col2a1*, *Aggrecan*, and *Col10a1* in ATDC5 cells (P5‐7). Following *circTspan3* overexpression, *Col2a1* mRNA peaked at day 7 and declined by day 14, while *Aggrecan* mRNA levels were elevated at days 7 and 14 compared to days 0 and 21. *Col10a1* expression increased substantially by day 21 (Figure 5G). Explant cultures treated with exosomal *circTspan3* for one month, exosomal *circTspan3* significantly increased both tibial and growth plate length, with a pronounced elongation of the HZ and elevated PZ/TZ and HZ/TZ ratios. Vacuolated structures also appeared within the growth plate (Figure 5H), indicating that exosomal *circTspan3* primarily promotes chondrocyte hypertrophy within the growth plate. Similarly, exosomal *circTspan3* enhanced chondrogenic differentiation, though with distinct temporal expression dynamics: *Aggrecan* and *Col2a1* mRNA peaked at day 14 and declined by day 21, whereas *Col10a1* expression increased at both days 14 and 21 (Figure 5I). To further assess the role of ferroptosis in exosomal *circTspan3*‐mediated cartilage growth, we treated ATDC5 cells (P5‐7) with ferroptosis agonists for 21 days following exosomal *circTspan3* exposure. Flow cytometry showed a marked reduction in the proportions of COL2⁺, AGGRECAN⁺, SOX9^+^, and COL10⁺ chondrocytes (Figure 5J,K,L,M; Figure S16), confirming that ferroptosis limits the chondrogenic effects of exosomal *circTspan3*. These findings underscore the importance of ferroptosis inhibition in *circTspan3*‐mediated cartilage development.

### 3.7 Exosomal circTspan3‐CS Hydrogel Promotes Cartilage Repair In Vivo

**FIGURE 6:**
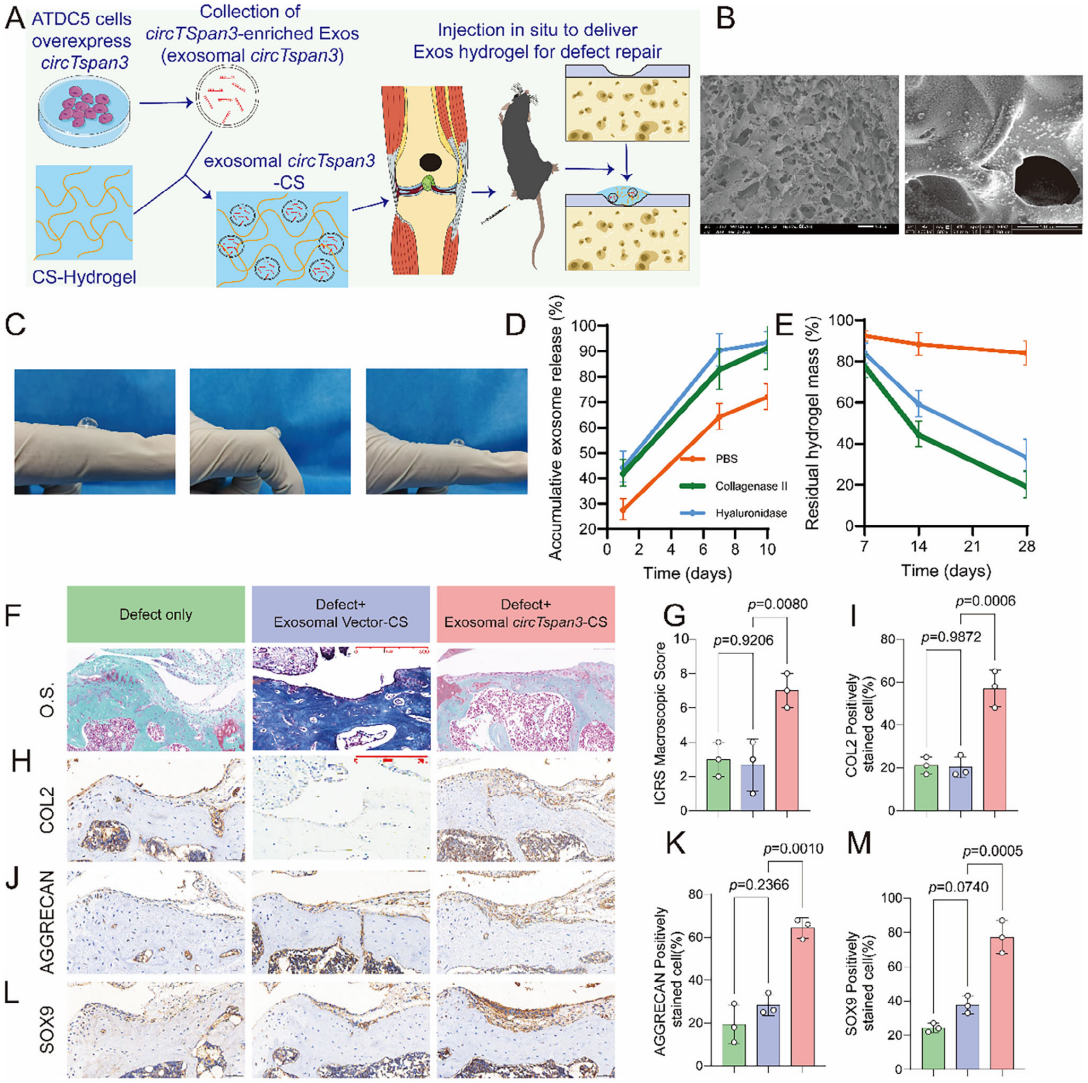
Exosome *circTspan3*‐chitosan hydrogel promotes cartilage regeneration in a mouse cartilage defect model. (A) Schematic diagram illustrating the preparation and application of exosomal *circTspan3*‐chitosan (Exo‐*circTspan3*‐CS) hydrogel for cartilage repair. (B) Scanning electron microscopy (SEM) showed a porous network structure in the hydrogel with exosomes distributed along the chitosan matrix. Representative images from *n* = 3 independent hydrogel preparations. (C) Adhesion testing of Exo‐*circTspan3*‐CS hydrogel demonstrating robust mechanical adherence to biological surfaces. Representative photographs showing hydrogel applied to a latex glove surface, maintaining strong adhesion even after repeated finger flexion. Representative images from *n* = 3 independent tests. (D) In vitro cumulative exosome release kinetics from Exo‐*circTspan3*‐CS hydrogel in phosphate‐buffered saline (PBS, pH 7.4) or enzyme‐supplemented buffer (collagenase II 0.25% + hyaluronidase 0.1%) at 37°C. Exosome release was quantified by nanoparticle tracking analysis (NTA) of supernatants collected at indicated time points and expressed as a cumulative percentage of total loaded exosomes. Data are presented as mean ± SEM. *n* = 3 independent hydrogel samples per condition. (E) In vitro degradation kinetics of chitosan hydrogel measured as residual dry mass percentage over 28 days in PBS (pH 7.4) or enzyme‐supplemented buffer (collagenase II 0.25% + hyaluronidase 0.1%) at 37°C. At each time point, hydrogels were retrieved, lyophilized, and weighed. Residual mass was expressed as percentage of the initial dry mass. Data are presented as mean ± SEM. *n* = 3 independent hydrogel samples per condition per time point. (F) Representative images of Safranin O staining of the defect area in each group 8 weeks after treatment. Scale bar: 250 µm. Representative images from *n* = 3‐5 mice per group. (G) Histological scoring of cartilage repair quality from panel F using a modified O'Driscoll scoring system. Scoring parameters included: surface regularity, structural integrity, thickness, integration with adjacent cartilage, cellular morphology, and matrix staining intensity. Scoring was performed by two independent observers blinded to treatment groups, with the average score reported. Data are presented as mean ± SEM. Statistical analysis: Kruskal‐Wallis test followed by Mann‐Whitney U test with Bonferroni correction for pairwise comparisons. *n* = 3‐5 mice per group. (H) Representative immunohistochemistry (IHC) images showing collagen type II (COL2) expression in repaired cartilage tissue at 8 weeks post‐injection. Scale bar: 250 µm. Representative images from *n* = 3 mice per group. (I) Quantification of COL2‐positive area from panel H. Data are presented as mean ± SEM. Statistical analysis: one‐way ANOVA followed by Tukey's post hoc test. *n* = 3‐5 mice per group. *p* values indicated in the figure. (J) Representative IHC images showing AGGRECAN expression in repaired cartilage tissue at 8 weeks post‐injection. Scale bar: 250 µm. Representative images from *n* = 3 mice per group. (K) Quantification of AGGRECAN‐positive area from panel J, performed using the same method as panel I. Data are presented as mean ± SEM. Statistical analysis: one‐way ANOVA followed by Tukey's post hoc test. *n* = 3 mice per group. *p* values indicated in the figure. (L) Representative IHC images showing SOX9 expression in repaired cartilage tissue at 8 weeks post‐injection. Scale bar: 250 µm. Representative images from *n* = 3 mice per group. (M) Quantification of SOX9‐positive cells from panel L. Data are presented as mean ± SEM. Statistical analysis: one‐way ANOVA followed by Tukey's post hoc test. *n* = 3 mice per group. *p* values indicated in the figure. Copyright attribution: The schematic illustration in panel A was created using graphical elements adapted from Servier Medical Art (https://smart.servier.com/) by Servier, licensed under a Creative Commons Attribution 3.0 Unported License (CC BY 3.0). All other panels contain original experimental data generated for this study.

Chitosan (CS) has been widely used as an injectable carrier due to its ability to enhance exosome stability, making it suitable for delivering exosome‐based therapeutics in vivo [42, 43]. To evaluate the reparative potential of exosomal *circTspan3* in cartilage injury, we formulated a composite hydrogel by combining exosomal *circTspan3* with chitosan (exosomal *circTspan3*‐CS) and injected it directly into cartilage defect sites in mice (Figure 6A). At 4 °C, the mixture remained a translucent liquid, but it rapidly transitioned into a hydrogel at 37 °C. SEM revealed a porous network structure within the hydrogel, with exosomes distributed along the internal surface of the CS matrix (Figure 6B). Compressive mechanical testing results (Figure S17) show no statistically significant difference in stress–strain slope between the exosomal *circTspan3* and exosomal *circTspan3*‐CS groups within the 0%–30% strain range, and their compression moduli are approximately 100 kPa. The difference between the two groups is not significant. In contrast, natural cartilage has a higher compression modulus of approximately 741 kPa, demonstrating the strongest mechanical bearing capacity. This design allows the hydrogel to maintain exosome stability and controlled release while providing sufficient mechanical support to resist external deformation. Furthermore, the lower modulus compared to natural cartilage ensures excellent injectability, biodegradability, and cell infiltration. Consequently, this temperature‐responsive CS hydrogel rapidly gels at 37°C, forming a scaffold that combines flexibility and mechanical stability, providing an ideal mechanical microenvironment for exosome retention and release in local tissues and tissue remodeling. Adhesion testing demonstrated robust mechanical adherence of the exosomal *circTspan3*‐CS hydrogel, maintaining strong adhesion even after finger flexion when applied to a glove surface (Figure 6C). To better mimic the intra‐articular enzymatic milieu, we quantified release and matrix resorption in enzyme‐supplemented buffers. Specifically, collagenase II and hyaluronidase accelerated both exosome liberation and chitosan mass loss relative to PBS, indicating that the PBS curve represents a conservative lower bound for in vivo release (Figure 6D). Consistent with this, in vitro enzymatic degradation showed a markedly faster decline in residual dry mass than PBS controls (Figure 6E).

Importantly, although exosomes are primarily liberated during the early phase, intra‐articular histology at 8 weeks reflects the durable downstream remodeling (matrix deposition, anabolic gene programs, and survival signaling) initiated by this early pulse, rather than the continued presence of the carrier itself. The absence of visible chitosan remnants in vivo at 8 weeks is therefore expected under physiological enzymatic turnover and tissue remodeling. Collectively, these findings confirmed the successful fabrication and favorable physicochemical properties of the exosomal *circTspan3*‐CS hydrogel. To evaluate its therapeutic efficacy, we employed a murine cartilage defect model. At 8 weeks post‐injection, Safranin O staining demonstrated substantial regeneration of hyaline‐like cartilage in the exosomal *circTspan3*‐CS–treated group, with near‐complete defect filling and cartilage transparency (Figure 6F,G). Immunohistochemical staining revealed significantly increased expression of COL2 (Figure 6H,I), AGGRECAN (Figure 6J,K), and SOX9 (Figure 6L,M) in the exosomal *circTspan3*‐CS group compared with both the untreated defect group and the exosomal vector‐CS control group. Given that enzymatic conditions in the joint expedite both exosome release and chitosan resorption relative to PBS, the 8‐week histological readouts predominantly reflect the downstream biological response initiated by the early exosome pulse (anabolic gene induction, reduced apoptosis/ferroptosis, and matrix deposition), rather than prolonged retention of the carrier material.

To evaluate systemic biocompatibility, we collected major organs at 8 weeks and performed H&E histology (heart, liver, spleen, lung, kidney). Across groups, tissue architecture remained intact with no consistent signs of hepatocellular necrosis, inflammatory infiltrates, fibrosis, pulmonary edema, or renal tubular injury (Figure S18). These findings support the in vivo safety of the Exo‐*circTspan3*–chitosan hydrogel at the tested regimen.

Together, these results indicate that exosomal *circTspan3*‐CS hydrogel effectively promotes cartilage regeneration and represents a promising therapeutic strategy for cartilage repair.

### 3.8 CircTspan3 Directly Binds to ANXA2 and Controls Anabolism and Ferroptosis Through ANXA2

**FIGURE 7:**
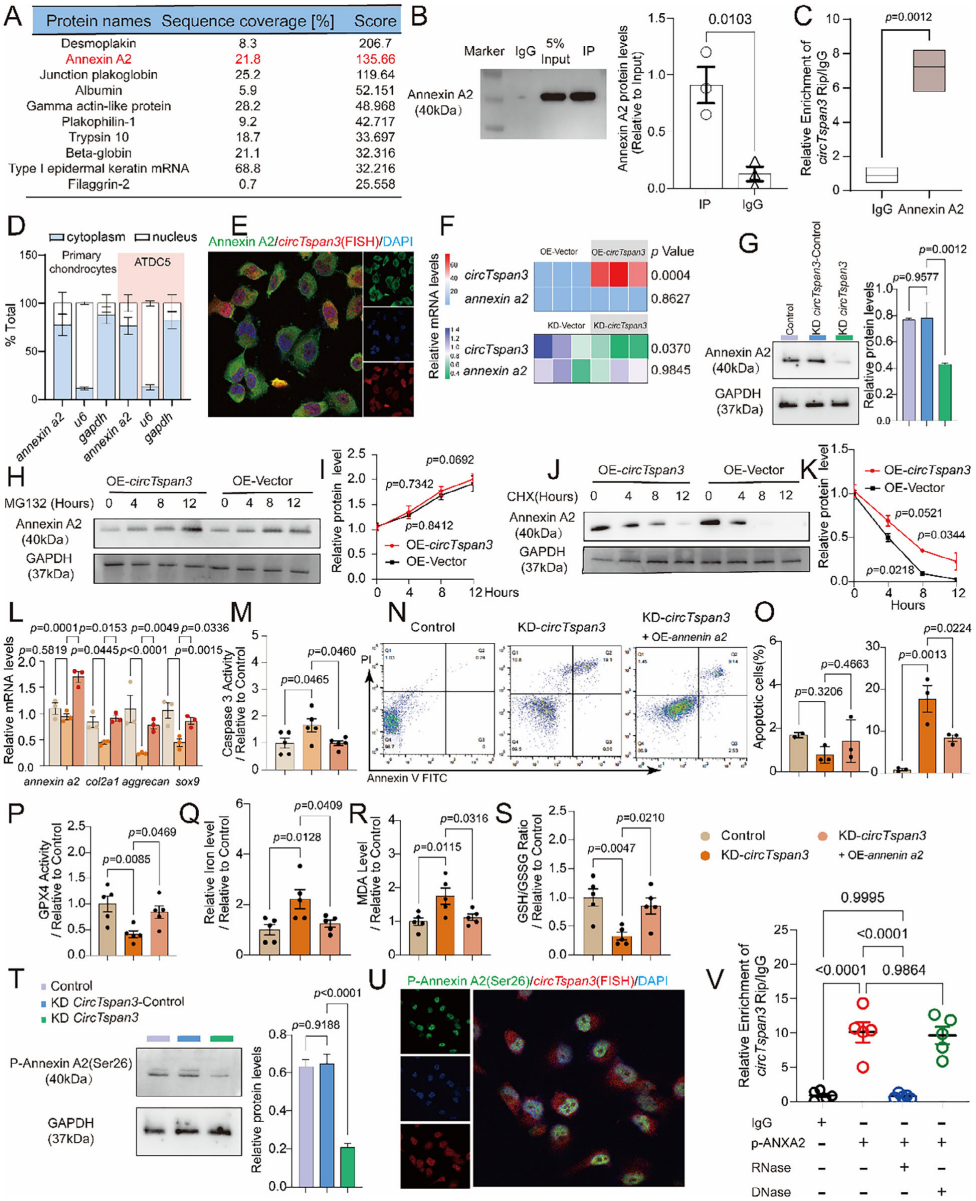
*CircTspan3* directly binds and stabilizes ANXA2, and the *circTspan3*–ANXA2 axis drives cartilage anabolism while inhibiting apoptosis and ferroptosis. (A) Biotin‐labeled *circTspan3* probe was added to ATDC5 cell (Passage 8‐12, P8‐12) lysate, and a list of potential binding proteins identified by mass spectrometry was obtained. (B) Western blot analysis of the pull‐down eluate confirmed the specific binding of *circTspan3* to ANXA2. The input lysate served as a positive control. Representative blots from *n* = 3 independent pull‐down experiments. Right panel: densitometric quantification of ANXA2 band intensity normalized to Input and expressed as fold enrichment relative to scrambled probe. Representative blots from *n* = 3 independent pull‐down experiments. Data are presented as mean ± SEM. Statistical analysis: paired two‐tailed Student's *t*‐test. *n* = 3 biological replicates. *p* values indicated in the figure. (C) RNA immunoprecipitation (RIP) assay confirming *circTspan3* enrichment in ANXA2‐containing complexes. ATDC5 cells (P8‐12) were lysed and immunoprecipitated with anti‐ANXA2 antibody or IgG control. Co‐precipitated *circTspan3* was quantified by RT‐qPCR, normalized to input, and expressed as fold enrichment relative to IgG control. Data are presented as mean ± SEM. Statistical analysis: paired two‐tailed Student's *t*‐test. *n* = 3 biological replicates. *p* values indicated in the figure. (D) RT‐qPCR analysis of *Anxa2* mRNA subcellular distribution following nuclear‐cytoplasmic fractionation in primary mouse chondrocytes (P0‐2) and ATDC5 cells (P8‐12). *n* = 3 biological replicates. Data presented as mean ± SEM. (E) Representative immunofluorescence (IF) and fluorescence in situ hybridization (FISH) images showing colocalization of *circTspan3* (red, detected by Cy3‐labeled probe) and ANXA2 protein (green, detected by anti‐ANXA2 antibody, followed by Alexa Fluor 488‐conjugated secondary antibody) in ATDC5 cells (P11). Nuclei were counterstained with DAPI (blue). Merged images demonstrate predominant cytoplasmic colocalization (yellow in merged channel). Scale bar: 20 µm. Representative images from *n* = 3 independent experiments. (F) RT‐qPCR analysis of *Anxa2* mRNA levels in ATDC5 cells (P8‐12) following *circTspan3* overexpression (OE) or knockdown (KD). Cells were transfected with *circTspan3*‐expressing plasmid, siRNA targeting *circTspan3*, or corresponding controls for 48 h. Total RNA was extracted, and *Anxa2* mRNA was quantified by RT‐qPCR and normalized to *Gapdh*. Data are presented as mean ± SEM. Statistical analysis: unpaired two‐tailed Student's *t*‐test for each comparison. *n* = 3 biological replicates. *p* values indicated in the figure. (G) Western blot analysis and densitometry quantification of ANXA2 protein levels in ATDC5 cells (P8‐12) following *circTspan3* knockdown. Cells were transfected with si‐*circTspan3* or scrambled control for 48 h. Total protein lysates (30 µg per lane) were analyzed by Western blot using anti‐ANXA2 antibody and anti‐GAPDH antibody. Representative blots from *n* = 3 independent experiments. Data are presented as mean ± SEM. Statistical analysis: one‐way ANOVA followed by Tukey's post hoc test. *n* = 3 biological replicates. *p* values indicated in the figure. (H) Western blot analysis examining the effect of *circTspan3* on ANXA2 protein synthesis rate. ATDC5 cells (P8‐12) were transfected with control or *circTspan3*‐overexpressing plasmid for 48 h, then treated with proteasome inhibitor MG132 (10 µm) for 0, 4, 8 or 12 h to block protein degradation. Total protein lysates (30 µg per lane) were analyzed by Western blot using anti‐ANXA2 and anti‐GAPDH antibodies. Representative blots from *n* = 3 independent experiments. (I) Densitometry quantification of ANXA2 protein synthesis rate from panel H. ANXA2 band intensities were normalized to GAPDH and expressed as fold change relative to 0‐h time point within each group. Data are presented as mean ± SEM. Statistical analysis: unpaired two‐tailed Student's *t*‐test. *n* = 3 biological replicates. *p* values indicated in the figure. (J) Western blot analysis of ANXA2 protein half‐life by cycloheximide (CHX) chase assay. ATDC5 cells were transfected with control or *circTspan3*‐overexpressing plasmid for 48 h, then treated with CHX (50 µg/mL, Sigma C4859) to block new protein synthesis for 0, 4, 8, or 12 h. Total protein lysates (30 µg per lane) were analyzed by Western blot using anti‐ANXA2 and anti‐GAPDH antibodies. Representative blots from *n* = 3 independent experiments. (K) Densitometry quantification and exponential decay curve fitting of ANXA2 protein degradation from panel J. ANXA2 band intensities were normalized to GAPDH and expressed as a percentage of 0‐h time point within each group. Data are presented as mean ± SEM. Statistical analysis: two‐way ANOVA (*circTspan3* status × time point) followed by Bonferroni's post hoc test. *n* = 3 biological replicates per time point. *p* values indicated in the figure. (L) RT‐qPCR analysis of *Anxa2*,  and cartilage anabolic markers (*Col2a1*, *Aggrecan*, *Sox9*) levels in ATDC5 cells (P8‐11) following rescue experiments. Cells were transfected with: (i) control siRNA(left), (ii) KD‐*circTspan3(middle)*, or (iii) KD‐*circTspan3* + *Anxa2* OE plasmid(right). RNA was extracted 48 h post‐transfection and analyzed by RT‐qPCR. Data normalized to *Gapdh* and expressed as fold change relative to control. Two‐way ANOVA followed by Tukey's post hoc test. *n* = 3 biological replicates. Data presented as mean ± SEM. (M) Caspase‐3 enzymatic activity assay in ATDC5 cells (P5‐7) with *circTspan3* knockdown alone or combined with *Anxa2* overexpression rescue for 48 h. Cells were transfected with: (i) control siRNA(left), (ii) KD‐*circTspan3(middle)*, or (iii) KD‐*circTspan3* + *Anxa2* OE plasmid(right). Caspase‐3 activity was expressed as fold change relative to KD‐Control group (mean normalized to 1). Data are presented as mean ± SEM. Statistical analysis: one‐way ANOVA followed by Tukey's post hoc test. *n* = 5 biological replicates. *p* values indicated in the figure. (N) Flow cytometry analysis of apoptosis (Annexin V‐FITC/PI staining) in rescue experiments. ATDC5 cells (1×10⁶ per sample) were treated as described in panel L and analyzed after 48 h. Early apoptotic cells (Annexin V⁺/PI^−^) and late apoptotic cells (Annexin V⁺/PI⁺) were quantified. One‐way ANOVA followed by Tukey's post hoc test. *n* = 3 biological replicates. (O) Flow cytometry analysis of apoptotic cells using Annexin V‐FITC/PI staining in ATDC5 cells (P5‐7) with *circTspan3* knockdown alone or combined with *Anxa2* overexpression rescue for 48 h. Data are presented as mean ± SEM (apoptotic percentage is not normalized; raw percentages reported). Statistical analysis: one‐way ANOVA followed by Tukey's post hoc test. *n* = 3 biological replicates. *p* values indicated in the figure. (P) Glutathione peroxidase 4 (GPX4) enzymatic activity assay in ATDC5 cells (P5‐7) with *circTspan3* knockdown alone or combined with *Anxa2* overexpression rescue for 48 h. GPX4 activity was expressed as a fold change relative to KD‐Control group. Data are presented as mean ± SEM. Statistical analysis: one‐way ANOVA followed by Tukey's post hoc test. *n* = 5 biological replicates. *p* values indicated in the figure. (Q) Intracellular ferrous iron (Fe^2^⁺) measurement using FerroOrange probe in ATDC5 cells (P5‐7) with *circTspan3* knockdown alone or combined with *Anxa2* overexpression rescue for 48 h. Mean fluorescence intensity (MFI) was quantified by flow cytometry and expressed as fold change relative to KD‐Control group. Data are presented as mean ± SEM. Statistical analysis: one‐way ANOVA followed by Tukey's post hoc test. *n* = 5 biological replicates. *p* values indicated in the figure. (R) Malondialdehyde (MDA) assay measuring lipid peroxidation in ATDC5 cells (P5‐7) with *circTspan3* knockdown alone or combined with *Anxa2* overexpression rescue for 48 h. MDA level was expressed as fold change relative to KD‐Control group. Data are presented as mean ± SEM. Statistical analysis: one‐way ANOVA followed by Tukey's post hoc test. *n* = 5 biological replicates. *p* values indicated in the figure. (S) GSH/GSSG ratio measurement using GSH and GSSG Assay Kit in rescue experiments. One‐way ANOVA followed by Tukey's post hoc test. *n* = 5 biological replicates. Data presented as mean ± SEM. *p* values indicated in the figure. (T) Western blot analysis and densitometry quantification of phosphorylated ANXA2 at Ser26 (P‐ANXA2 Ser26) and total ANXA2 in ATDC5 cells following *circTspan3* knockdown. Cells were transfected with si‐*circTspan3* or scrambled control for 48 h. Total protein lysates (30 µg per lane) were analyzed by Western blot using anti‐P‐ANXA2(Ser26) antibody, and anti‐GAPDH antibody. Densitometry quantification (right panel) shows P‐ANXA2(Ser26) levels normalized to GAPDH. Data are presented as mean ± SEM. Statistical analysis: one‐way ANOVA followed by Tukey's post hoc test. *n* = 3 biological replicates. *p* values indicated in the figure. (U) Representative confocal immunofluorescence (IF) and fluorescence in situ hybridization (FISH) images showing subcellular localization of *circTspan3* (red, Cy3‐labeled probe) and phosphorylated ANXA2 at Ser26 (green, anti‐P‐ANXA2 Ser26 antibody 1:100, Alexa Fluor 488‐conjugated secondary antibody). Nuclei were counterstained with DAPI (blue). Images demonstrate nuclear colocalization (yellow in merged channel) of *circTspan3* and P‐ANXA2(Ser26), suggesting distinct nuclear functions of phosphorylated ANXA2.Scale bar: 10 µm. Representative images from *n* = 3 independent experiments. (V) RNA immunoprecipitation (RIP) assay confirming *circTspan3* enrichment in P‐ANXA2(Ser26)‐containing complexes. Primary mouse chondrocytes (P0) were lysed and immunoprecipitated with anti‐P‐ANXA2(Ser26) antibody or IgG control. Co‐precipitated *circTspan3* was quantified by RT‐qPCR, normalized to input (2%), and expressed as fold enrichment relative to IgG control. Data are presented as mean ± SEM. Data are presented as mean ± SEM. Statistical analysis: One‐way ANOVA followed by Tukey's post hoc test. *n* =5 biological replicates. *p* values indicated in the figure. Statistical analyses were performed using GraphPad Prism 10.0, with specific tests indicated for each panel.

To identify proteins that directly interact with *circTspan3* and mediate its functional effects, we employed an unbiased, proteome‐wide discovery approach. Rather than testing candidate proteins based on prior assumptions, we performed RNA pull‐down using biotinylated *circTspan3* probes (full‐length 606 nt) incubated with whole‐cell lysates from ATDC5 cells(P8‐12), followed by comprehensive liquid chromatography‐tandem mass spectrometry (LC‐MS/MS) to identify all co‐precipitated proteins (Figure 7A; Figure S19). This hypothesis‐free screen revealed ANXA2 as the most abundant *circTspan3*‐binding protein. RNA pull‐down results confirmed the binding of *circTspan3* to ANXA2 (Figure 7B). RIP results also showed that *circTspan3* was significantly enriched in the anti‐ANXA2 group compared with the IgG group (Figure 7C).

To explore whether *circTspan3* may also engage in miRNA‐mediated regulation, we performed bioinformatics prediction using miRanda software (sequence match threshold 140, minimum free energy ≤ −10 kcal/mol, strict Seed matching criteria). Analysis identified 12 miRNAs predicted to bind both circTspan3 and the 3′UTR of *Anxa2* mRNA: mmu‐miR‐6991‐3p, mmu‐miR‐133c, mmu‐miR‐3470b, mmu‐miR‐133b‐3p, mmu‐miR‐7656‐3p, mmu‐miR‐6898‐3p, mmu‐miR‐133a‐3p, mmu‐miR‐693‐3p, mmu‐miR‐208b‐5p, mmu‐miR‐208a‐5p, mmu‐miR‐488‐5p, and mmu‐miR‐6415 (Figure S20). While these predictions suggest potential miRNA‐mediated crosstalk, our focus remained on direct protein interactions, as *circTspan3* localization and functional effects were predominantly governed by ANXA2.

RNA nuclear‐cytoplasmic fractionation showed that *Anxa2* was mainly distributed in the cytoplasm, both in primary chondrocytes (P0‐2) and ATDC5 cells (P8‐12) (Figure 7D). In ATDC5 cells, IF further observed that *circTspan3* and ANXA2 colocalized with the cytoplasm (Figure 7E). We used HDOCK SERVER (http://hdock.phys.hust.edu.cn/) to analyze the interaction between *circTspan3* and ANXA2. In the process, we predicted the secondary structure of *circTspan3* on the RNAfold Web server (http://rna.tbi.univie.ac.at/cgi‐bin/RNAWebSuite/RNAfold.cgi). Then, the obtained secondary structure was analyzed, and the tertiary structure of 3dRNA was further predicted (http://biophy.hust.edu.cn/3dRNA). In addition, we obtained the tertiary structure of ANXA2 from the Protein Database (https://www.wwpdb.org/). Subsequently, we imported this information into HDOCK SERVER. Docking analysis (Figure S21) reveals multiple hydrogen bonds and electrostatic contacts between *circTspan3* phosphates and ANXA2, with >70 interfacial residue–nucleotide contacts within 3.5 Å (Table S2), supporting an extensive RNA–protein interface.

The above data indicate that *circTspan3* can interact with ANXA2. Interestingly, changes in *circTspan3* did not affect the mRNA level of *Anxa2* (Figure 7F), while after knocking down *circTspan3*, the ANXA2 protein content decreased (Figure 7G). In addition, we found that in the presence of the proteasome inhibitor MG132, there was no statistical difference in the protein synthesis rate of ANXA2 in ATDC5 cells with or without *circTspan3* overexpression compared with control cells (Figure 7H,I). To determine whether *circTspan3* maintains the stability of ANXA2 protein, we measured the half‐life of ANXA2. In the presence of CHX, the degradation rate of ANXA2 in ATDC5 cells OE *circTspan3* was significantly lower than that in the group without OE *circTspan3* (Figure 7J,K).

Then, we overexpressed *Anxa2* on the basis of knocking down *circTspan3(*Figure S22). Our results showed that OE‐*Anxa*2 could reverse the negative effects of KD‐*circTspan3* on ATDC5 cells (P11). Specifically, it increased cartilage anabolism and inhibited apoptosis and ferroptosis levels (Figure 7L–S). Similarly, *Anxa2* also plays a decisive role in the positive role of exosomal *circTspan3* in ATDC5 cells (P5‐7). Specifically, knocking down *Anxa2* on the basis of exosomal *circTspan3* weakened the ability of exosomal *circTspan3* to promote cartilage anabolism and inhibit apoptosis and ferroptosis levels, while overexpression of *Anxa2* amplified the above effects of exosomal *circTspan3* (Figure S23).

Previous studies have implicated ANXA2 in secretory vesicles, with Ser phosphorylation of ANXA2 being crucial for cellular secretion processes. Therefore, we focused on Ser26 for further investigation. Following KD *circTspan3*, phosphorylated ANXA2 (Ser26) levels notably decreased (Figure 7T).

IF combined with FISH staining showed that *circTspan3* (red) and P‐ANXA2 (Ser26) (Green) colocalized in the cell nucleus (Figure 7U), suggesting that ANXA2 and P‐ANXA2 (Ser26) may play different functions for *circTspan3*. To confirm the direct interaction between p‐ANXA2(Ser26) and c*ircTspan3*, we performed whole‐cell RIP with anti‐p‐ANXA2(Ser26) antibody, along with IgG and Input controls. Compared with IgG‐IP, p‐ANXA2(Ser26)‐IP showed significant enrichment of *circTspan3*; this enrichment decreased to background after RNase A treatment, while DNase I did not change this trend, supporting RNA‐dependent specific binding (Figure 7V).

### 3.9 Ser26 Phosphorylation of ANXA2 Differentially Regulates circTspan3 Trafficking and Chondroprotective Functions

Previous studies have shown that N‐terminal serine phosphorylation of ANXA2 regulates its subcellular localization and RNA‐binding capacity [36], prompting us to examine whether phosphorylation status differentially controls *circTspan3* trafficking.

**FIGURE 8:**
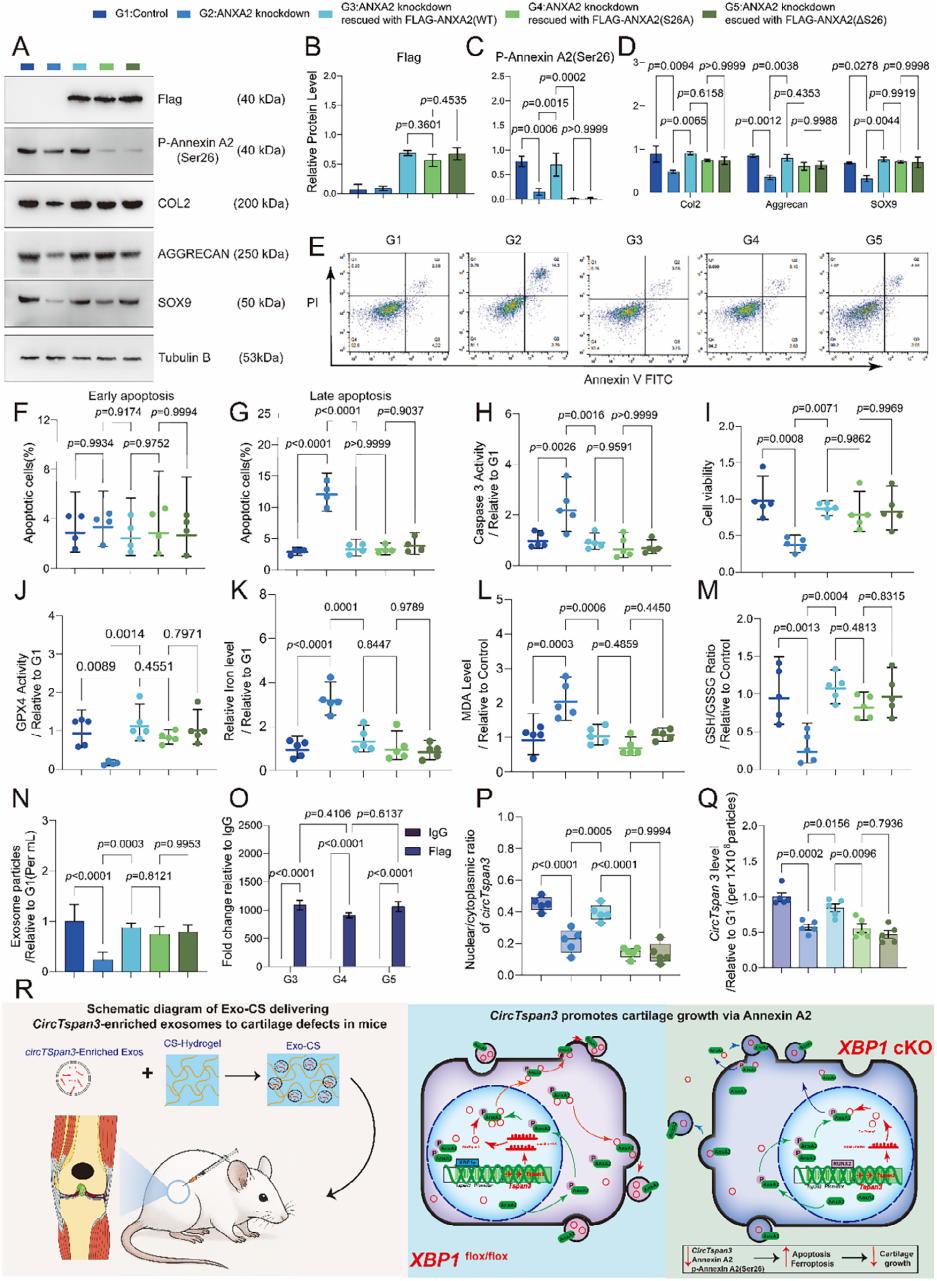
Ser26 phosphorylation of ANXA2 differentially regulates *circTspan3* subcellular trafficking but not chondroprotective functions. (A) Western blot analysis of ANXA2 phosphorylation status, protein expression, and cartilage markers across five experimental groups in ATDC5 cells (Passage 5‐7, P5‐7): G1, control (scrambled siRNA); G2, ANXA2 knockdown (si‐ANXA2); G3, knockdown rescued with FLAG‐tagged wild‐type ANXA2 (WT); G4, rescued with FLAG‐ANXA2(S26A) point mutant; G5, rescued with FLAG‐ANXA2(ΔS26) deletion mutant. Cells were transfected with siRNA (50 nM) for 24 h, followed by plasmid transfection (2 µg per well in 6‐well plates) for an additional 48 h. Total protein lysates (30 µg per lane) were separated by 10% SDS‐PAGE and transferred to PVDF membranes.P‐ANXA2(Ser26), and FLAG‐tagged exogenous ANXA2, cartilage anabolic markers (COL2A1, AGGRECAN, SOX9) and Tubulin B were detected. l Representative blots from *n* = 3 independent experiments. (B) Densitometric quantification of total ANXA2 protein (exogenous FLAG‐ANXA2) from panel A. Band intensity was normalized to Tubulin B. Data are presented as mean ± SEM. Statistical analysis: one‐way ANOVA followed by Tukey's post hoc test. *n* = 3 biological replicates. *p* values indicated in the figure. (C) Densitometric quantification of P‐ANXA2(Ser26) from panel A. Band intensity was normalized to Tubulin B. Data are presented as mean ± SEM. Statistical analysis: one‐way ANOVA followed by Tukey's post hoc test. *n* = 3 biological replicates. *p* values indicated in the figure. (D) Densitometric quantification of cartilage anabolic markers (COL2A1, AGGRECAN, SOX9) from panel A. Band intensities were normalized to Tubulin B. Data are presented as mean ± SEM. Statistical analysis: two‐way ANOVA (ANXA2 construct × protein marker) followed by Tukey's post hoc test. *n* = 3 biological replicates. *p* values indicated in the figure. (E)Representative flow cytometry dot plots showing Annexin V‐FITC/PI staining for apoptosis detection in ATDC5 cells (P5‐7) across the five experimental groups. Quadrants indicate: Q1 (necrotic cells, PI⁺/Annexin V^−^), Q2 (late apoptotic cells, PI⁺/Annexin V⁺), Q3 (live cells, PI^−^/Annexin V^−^), Q4 (early apoptotic cells, PI^−^/Annexin V⁺). Representative plots from *n* = 5 independent experiments. (F) Quantification of early apoptotic cells (Q4 quadrant) from panel E. Data are presented as mean ± SEM (apoptotic percentage is not normalized; raw percentages reported). Statistical analysis: one‐way ANOVA followed by Tukey's post hoc test. *n* = 5 biological replicates. *p* values indicated in the figure. (G) Quantification of late apoptotic cells (Q2 quadrant) from panel E. Data are presented as mean ± SEM (apoptotic percentage is not normalized; raw percentages reported). Statistical analysis: one‐way ANOVA followed by Tukey's post hoc test. *n* = 5 biological replicates. *p* values indicated in the figure. (H) Caspase‐3 enzymatic activity assay in ATDC5 cells (P5‐7) across the five experimental groups. Caspase‐3 activity was measured as relative fluorescence units (RFU) per mg protein and expressed as fold change relative to G1 Control. Data are presented as mean ± SEM. Statistical analysis: one‐way ANOVA followed by Tukey's post hoc test. *n* = 5 biological replicates. *p* values indicated in the figure. (I) Cell viability assessment by CCK‐8 assay in ATDC5 cells (P5‐7) across the five experimental groups. Absorbance at 450 nm was measured and expressed as fold change relative to G1 Control. Data are presented as mean ± SEM. Statistical analysis: one‐way ANOVA followed by Tukey's post hoc test. *n* = 5 biological replicates. *p* values indicated in the figure. (J) Glutathione peroxidase 4 (GPX4) enzymatic activity assay in ATDC5 cells (P5‐7) across the five experimental groups. GPX4 activity was measured as nmol NADPH oxidized per minute per mg protein and expressed as fold change relative to G1 Control. Data are presented as mean ± SEM. Statistical analysis: one‐way ANOVA followed by Tukey's post hoc test. *n* = 3 biological replicates. *p* values indicated in the figure. (K) Intracellular ferrous iron (Fe^2^⁺) measurement using FerroOrange probe in ATDC5 cells (P5‐7) across the five experimental groups. Mean fluorescence intensity (MFI) was quantified by flow cytometry and expressed as fold change relative to G1 Control. Data are presented as mean ± SEM. Statistical analysis: one‐way ANOVA followed by Tukey's post hoc test. *n* = 5 biological replicates. *p* values indicated in the figure. (L) Malondialdehyde (MDA) assay measuring lipid peroxidation in ATDC5 cells (P5‐7) across the five experimental groups. MDA concentration was expressed as fold change relative to G1 Control. Data are presented as mean ± SEM. Statistical analysis: one‐way ANOVA followed by Tukey's post hoc test. *n* = 5 biological replicates. *p* values indicated in the figure. (M) Reduced glutathione to oxidized glutathione ratio (GSH/GSSG) measurement in ATDC5 cells (P5‐7) across the five experimental groups. GSH/GSSG ratio was expressed as fold change relative to G1 Control. Data are presented as mean ± SEM. Statistical analysis: one‐way ANOVA followed by Tukey's post hoc test. *n* = 5 biological replicates. *p* values indicated in the figure. (N) Nanoparticle tracking analysis (NTA) quantification of exosome production in ATDC5 cells (P5‐7) across the five experimental groups. Total exosome particle concentration in conditioned medium (48‐h collection) was measured and expressed as fold change relative to G1 Control. Data are presented as mean ± SEM. Statistical analysis: one‐way ANOVA followed by Tukey's post hoc test for each assay. *n* = 5 biological replicates. *p* values indicated in the figure. (O) RNA immunoprecipitation (RIP) using anti‐FLAG antibody to assess *circTspan3* binding to FLAG‐tagged ANXA2 variants in the cytoplasm. *CircTspan3* enrichment in FLAG‐IP was quantified by RT‐qPCR, normalized to input (2%), and expressed as fold change relative to G3 (FLAG‐ANXA2‐WT) group. Data are presented as mean ± SEM. Statistical analysis: one‐way ANOVA followed by Tukey's post hoc test for each assay. *n* = 5 biological replicates. *p* values indicated in the figure. (P) RT‐qPCR analysis of *circTspan3* subcellular distribution following nuclear‐cytoplasmic fractionation in ATDC5 cells (P5‐7) across the five experimental groups. Nuclear and cytoplasmic RNA were extracted separately, and *circTspan3* levels in each fraction were normalized to *U6* snRNA (nuclear marker) or *Gapdh* mRNA (cytoplasmic marker), respectively. Nuclear/cytoplasmic ratio was calculated and expressed as fold change relative to G1 Control. Data are presented as mean ± SEM. Statistical analysis: one‐way ANOVA followed by Tukey's post hoc test. *n* = 5 biological replicates. *p* values indicated in the figure. (Q) RT‐qPCR quantification of *circTspan3* content in isolated exosomes (normalized to exosome particle number by NTA) from ATDC5 cells (P5‐7) across the five experimental groups. Exosomal RNA was extracted from equal numbers of exosomes (1×10^9^ particles), and *circTspan3* was quantified by RT‐qPCR normalized to exosomal *U6* snRNA. Exosomal *circTspan3* per particle was expressed as fold change relative to G1 Control. Data are presented as mean ± SEM. Statistical analysis: one‐way ANOVA followed by Tukey's post hoc test. *n* = 5 biological replicates. *p* values indicated in the figure. (R) Schematic model illustrating the bifunctional role of ANXA2 in *circTspan3* biology. The main research contents of this study. The left figure shows that exosome *circTspan3*‐CS hydrogel has the ability to repair cartilage defects in mice. In terms of specific mechanisms (right figure), it shows that XBP1s in chondrocytes promotes the transcription and translation of *Tspan3*, and *Tspan3* undergoes selective splicing to produce *circTspan3* consisting of exons 2‐6. ANNEXIN A2 in the cytoplasm is phosphorylated at the Ser26 site to form P‐ANNEXIN A2, which promotes the transport of *circTspan3* from the nucleus to the cytoplasm. ANNEXIN A2 in the cytoplasm encapsulates *circTspan3* in exosomes and secretes it out of the cell. After secretion, *circTspan3* can prevent chondrocyte apoptosis and ferroptosis, and enhance the expression of anabolic factors such as COL2, AGGRECAN, and SOX9. Copyright attribution: The schematic illustration in panel R was created using graphical elements adapted from Servier Medical Art (https://smart.servier.com/) by Servier, licensed under a Creative Commons Attribution 3.0 Unported License (CC BY 3.0).

To definitively determine whether ANXA2's functions depend on Ser26 phosphorylation or total protein levels, we performed comprehensive rescue experiments using both Ser26→Ala (S26A) point mutant and ΔSer26 (ΔS26) deletion mutant alongside wild‐type ANXA2 (WT). Five experimental groups were established: control (G1), ANXA2 knockdown (G2), and knockdown rescued with FLAG‐ANXA2(WT) (G3), FLAG‐ANXA2(S26A) (G4), or FLAG‐ANXA2(ΔS26) (G5). Western blot confirmed equivalent expression of all three FLAG‐tagged constructs, while phospho‐Ser26‐specific antibody detected signal only in G1 and G3, confirming successful ablation of phosphorylation in both S26A and ΔS26 mutants (Figure 8A–C). We first examined whether ANXA2's protective effects on chondrocytes require Ser26 phosphorylation. ANXA2 knockdown (G2) substantially reduced cartilage anabolic markers—COL2A1, AGGRECAN, and SOX9 protein levels decreased to approximately 50%–60% of control. Strikingly, all three ANXA2 variants fully restored these markers to control levels, with no significant differences among WT (G3), S26A (G4), and ΔS26 (G5) groups (Figure 8A,D). This demonstrates that cartilage anabolism does not require Ser26 phosphorylation.

Apoptosis assays revealed a similar pattern. Flow cytometry showed that apoptotic cell percentage increased from ∼2% in controls to ∼15% following ANXA2 knockdown. All three ANXA2 forms—WT, S26A, and ΔS26—equally reduced apoptosis back to ∼2%–5% (Figure 8E–G). Caspase‐3 activity and cell viability measurements corroborated these findings (Figure 8H,I), indicating that anti‐apoptotic effects are Ser26‐independent.

Ferroptosis‐related markets further confirmed this conclusion. ANXA2 knockdown reduced GPX4 activity (Figure 8J), increased intracellular Fe^2^⁺ accumulation (Figure 8K), elevated lipid peroxidation (Figure 8L), and decreased GSH/GSSG ratios (Figure 8M). Remarkably, WT, S26A, and ΔS26 equivalently reversed all these ferroptosis changes, demonstrating that ferroptosis inhibition is Ser26‐independent. To investigate exosome‐related functions, we quantified exosome production using nanoparticle tracking analysis. ANXA2 knockdown reduced exosome particle counts to ∼70% of control. All three ANXA2 variants—WT, S26A, and ΔS26—equally restored exosome production to control levels (Figure 8N), indicating that exosome biogenesis is Ser26‐independent. Finally, RNA immunoprecipitation using anti‐FLAG antibody revealed that *circTspan3* was enriched to similar extents in all three ANXA2 forms, with no significant differences among WT, S26A, and ΔS26 (Figure 8O). This demonstrates that cytoplasmic ANXA2‐circTspan3 binding is Ser26‐independent.

In striking contrast to the phenotypic rescue results, nuclear‐cytoplasmic fractionation revealed that *circTspan3* subcellular distribution critically depends on Ser26 phosphorylation. In control cells (G1), *circTspan3* exhibited predominantly cytoplasmic localization (nuclear/cytoplasmic ratio ∼0.40). ANXA2 knockdown (G2) reduced this ratio to ∼0.18, suggesting impaired nuclear export or cytoplasmic retention. Wild‐type ANXA2 (G3) fully restored the ratio to ∼0.38. However, both S26A (G4) and ΔS26 (G5) failed to rescue nuclear export, maintaining low ratios of ∼0.17 and ∼0.14, respectively (Figure 8P). These data demonstrate that *circTspan3* nuclear export specifically requires Ser26 phosphorylation. P‐Ser26 Western blot confirmed that Ser26 phosphorylation was present in G1 and G3 but absent in G4 and G5, directly correlating with the nuclear export phenotype (Figure 8A,B).

Most strikingly, when we quantified *circTspan3* content within isolated exosomes (normalized to particle number), we observed a clear Ser26 dependence. ANXA2 knockdown (G2) reduced exosomal *circTspan3* to ∼35% of control levels. Wild‐type ANXA2 (G3) fully restored *circTspan3* loading to ∼95% of control. However, S26A (G4) and ΔS26 (G5) achieved only partial restoration, reaching ∼60% and ∼55% of control levels, respectively (Figure 8Q). Critically, this selective cargo loading defect occurred despite normal exosome biogenesis in G4 and G5, demonstrating that selective packaging of *circTspan3* into exosomes requires Ser26 phosphorylation, mechanistically distinct from the Ser26‐independent exosome production machinery.

Across all the above functional assays, the S26A point mutant (G4) and ΔS26 deletion mutant (G5) produced statistically indistinguishable results. In Ser26‐independent assays (cartilage anabolism, apoptosis, ferroptosis, exosome biogenesis, cytoplasmic binding), both mutants performed identically to wild‐type (G3). In Ser26‐dependent assays (nuclear export, exosomal loading), both mutants failed equally to rescue the phenotype. This concordance demonstrates that the deletion of mutants does not cause non‐specific structural disruptions and that observed phenotypes are specifically attributable to loss of Ser26 phosphorylation.

These findings establish a bifunctional model for ANXA2 in *circTspan3* biology (Figure 8R). Ser26 phosphorylation specifically enables *circTspan3* nuclear export and selective loading into exosomes. In contrast, total ANXA2 protein, regardless of phosphorylation status, mediates cytoplasmic binding to *circTspan3*, general exosome biogenesis, and execution of downstream chondroprotective signaling.

The observation that all functional phenotypes (anabolism, anti‐apoptosis, anti‐ferroptosis) are Ser26‐independent indicates that cytoplasmic *circTspan3* pools—regardless of how they reach the cytoplasm—execute biological functions through Ser26‐independent ANXA2 interactions. Thus, Ser26 phosphorylation primarily governs *circTspan3* subcellular trafficking and intercellular communication via exosomal transfer, rather than its intracellular chondroprotective activities.

## 4 Discussion

In this study, we identified *circTspan3* as a previously unrecognised yet essential downstream effector of XBP1s signalling, orchestrating cartilage development through both intracellular and extracellular pathways. Our findings delineate a novel XBP1s –*circTspan3* – ANXA2 regulatory circuit. This pathway sustains chondrocyte anabolism, protects against apoptosis and ferroptosis, and enables paracrine circRNA communication through exosome‐mediated transfer. Strikingly, we translated this mechanistic insight into a clinically adaptable, cell‐free therapy. An injectable chitosan hydrogel embedded with *circTspan3*‐rich exosomes achieved robust in vivo cartilage regeneration. Together, our findings bridge ER stress adaptation, non‐coding RNA function, and exosome biology in skeletal homeostasis, opening new avenues for regenerative medicine and precision therapies targeting cartilage disorders.

Mounting evidence indicates that XBP1s, a key transcription factor in UPR, plays an essential role in skeletal development and extracellular matrix homeostasis by modulating chondrocyte proliferation and differentiation [5, 7, 44, 45, 46, 47]. While prior studies have characterized XBP1s‐regulated protein‐coding genes, our work broadens this regulatory landscape by identifying *circTspan3* as a novel non‐coding RNA target directly governed by XBP1s. Through ChIP‐qPCR, we mapped canonical XBP1s‐binding motifs within the *Tspan3* promoter region, validating direct transcriptional regulation.

Although XBP1s increases both linear and circular *Tspan3*, a tunicamycin pulse–chase followed by transcriptional shutoff established a short “linear‐low/circular‐high” window. During this period, *Col2a1*, *Sox9*, and *Aggrecan* remained elevated, and *circTspan3* nuclear access increased modestly while staying cytoplasm‐predominant. Thus, within our chondrocyte system and timeframe, the early anabolic and trafficking‐related effects are *circTspan3*‐driven and independent of linear *Tspan3* protein, suggesting independence rather than synergy or antagonism. We do not exclude longer‐term or tissue‐specific roles of linear *Tspan3* that future in vivo work may reveal.

The strong positive correlation between *Xbp1s*, *circTspan3*, and key anabolic genes (*Sox9*, *Col2a1*, *Aggrecan*) across murine developmental stages underscores a conserved XBP1s—CircRNA—matrix axis, advancing our understanding of non‐coding RNA integration in endochondral ossification. One of the most compelling aspects of *circTspan3* is its ability to concurrently suppress apoptosis and ferroptosis — two cell death pathways known to compromise cartilage integrity under pathological conditions such as osteoarthritis and intervertebral disc degeneration [23, 24]. Our findings position *circTspan3* within an expanding repertoire of functionally specialized cartilage circRNAs, while revealing a distinctive chondroprotective profile that addresses critical gaps in current therapeutic paradigms. Unlike *circSERPINE2* [16], *circPDE4D* [17], *circHIPK3* [18], and *circCDK14* [19]—which predominantly operate through single‐pathway mechanisms (either anti‐apoptotic or anti‐catabolic)—*circTspan3* uniquely integrates dual chondroprotective programs by simultaneously suppressing apoptosis (via Caspase‐3 inhibition) and ferroptosis (via GPX4 preservation, lipid peroxidation reduction, and iron homeostasis restoration). This comprehensive action addresses a fundamental limitation of prior circRNA therapeutics: the pathological landscape of osteoarthritis involves concurrent activation of multiple cell death pathways, with oxidative stress, iron dysregulation, and lipid peroxidation converging to drive chondrocyte loss [20, 21, 24, 26].

Recent mechanistic insights into cartilage ferroptosis further underscore the translational relevance of *circTspan3*'s dual functionality. Wang et al. demonstrated that mechanical overloading—a primary OA risk factor—induces GPX4‐regulated ferroptosis through Piezo1 channel‐mediated calcium influx [26], while Zheng et al. showed that P21 confers ferroptosis resistance by stabilizing GPX4 protein against proteasomal degradation [27]. Moreover, systemic metabolic factors can remotely regulate cartilage ferroptosis: Guan et al. reported that gut microbiota‐derived capsiate suppresses knee OA‐induced ferroptosis by modulating HIF‐1α/SLC2A1 signaling [28]. These studies collectively establish ferroptosis as a druggable target in OA, yet no prior circRNA has demonstrated coordinated suppression of both ferroptosis and apoptosis as we observe with *circTspan3*. Beyond hydrogel‐based release systems, converging evidence from stem cell–derived exosome studies reinforces the therapeutic potential of exosomal cargo in cartilage regeneration. Supporting these findings, recent advances in stem cell‐derived exosome therapies have demonstrated remarkable chondroprotective capacity through complementary mechanisms. Chen et al. showed that cartilage stem/progenitor cells‐derived exosomes (CSPCs‐EXOs) effectively inhibit chondrocyte apoptosis, with CDK9 playing a crucial role in mediating these protective effects [48]. Furthermore, transcriptomic profiling revealed that CSPCs‐EXOs modulate immune pathways and reduce inflammatory cytokines such as TNF and IL‐17 [49], demonstrating that exosome‐based therapies can simultaneously address multiple pathological mechanisms in OA. These studies provide complementary evidence that exosomal delivery of chondroprotective factors—such as *circTspan3* identified in the present work—represents a viable therapeutic strategy for cartilage regeneration by integrating anti‐ferroptotic, anti‐apoptotic, and immunomodulatory functions within a single biological platform.

Beyond its dual anti‐death activity, *circTspan3* exhibits three additional distinguishing features. First, its biogenesis is directly governed by XBP1s, establishing an unprecedented regulatory link between proteostasis networks and cartilage‐protective circRNAs. This contrasts with other cartilage circRNAs whose transcriptional regulation remains largely undefined. Second, *circTspan3* engages in active exosome‐mediated paracrine signaling, enabling donor chondrocytes to confer chondroprotection on distant recipient cells—a property not reported for *circSERPINE2*, *circPDE4D*, *circHIPK3*, or *circCDK14*. Third, our demonstration of phosphorylation‐dependent (ANXA2 Ser26) circRNA trafficking reveals a novel post‐translational regulatory layer governing circRNA subcellular distribution and secretion, expanding the functional repertoire of Annexin proteins beyond their canonical roles in membrane dynamics [50, 51, 52].

While *circTRIM25* also engages ferroptosis regulation [22], it lacks the apoptotic suppression and paracrine signaling capacities of *circTspan3*. Conversely, while *circSERPINE2* demonstrates robust in vivo cartilage protection [16], its mechanism is restricted to apoptosis/ECM pathways without addressing ferroptosis. *CircTspan3* thus represents the first circRNA to integrate: (1) XBP1s‐dependent transcriptional control, (2) dual anti‐apoptotic and anti‐ferroptotic programs, (3) ANXA2‐mediated exosomal trafficking, and (4) paracrine chondroprotection. This multifaceted mechanism may confer superior therapeutic efficacy in the complex, multi‐pathway pathological milieu of OA, where targeting a single node often proves insufficient for durable cartilage preservation [17, 44].

Exosomes derived from *circTspan3*‐overexpressing chondrocytes delivered functional *circTspan3* to independent recipient cells, enhancing anabolic gene expression and reducing stress‐induced cell death. In vivo, *circTspan3* is detectable in EVs from peripheral blood; we therefore interpret this as a systemic, EV‐associated pool rather than proof of a defined tissue source or long‐distance endocrine signaling. Future work using cartilage‐restricted EV reporters (e.g., tissue‐specific CD63/tetraspanin tags or Cre‐dependent Nanoluc‐EV systems) and selective perturbation of EV biogenesis will be necessary to trace source(s) and to test whether *circTspan3* undergoes bona fide endocrine‐like trafficking.

Mechanistically, our study reveals an unprecedented role for ANXA2 and its phosphorylated form (P‐ANXA2 Ser26) in coordinating *circTspan3* trafficking. We show that P‐ANXA2 Ser26 is required for nuclear export of *circTspan3*, while ANXA2 itself is essential for its packaging into exosomes. These findings build on recent insights into RNA‐binding capabilities of Annexin family proteins [50, 51, 52, 53] and provide a mechanistic basis for how specific post‐translational modifications regulate circRNA compartmentalization and secretion. Our structural modeling and IF‐FISH data further support this bifunctional role.

Importantly, we translated these findings into a therapeutic platform by formulating exosomal *circTspan3* within a chitosan‐based hydrogel. Chitosan, known for its biocompatibility and exosome stabilization capacity [42], supports an early, enzyme‐augmented release pulse in vivo that is sufficient to prime durable chondrogenic remodeling. This mechanistic view reconciles near‐complete early release with long‐term histological benefit, consistent with pulse‐initiated cascades reported for bioactive cargo delivered from degradable hydrogels [54].

Because circRNAs are stable and exosome‐packaged, *circTspan3* is a promising liquid‐biopsy biomarker. We envisage RT‐qPCR of *circTspan3* in serum or synovial fluid, with spike‐in normalization and optional EV enrichment, to support diagnosis/monitoring in disorders of cartilage growth such as growth‐plate dysplasia. Prospective pediatric studies should establish reference ranges, specificity related to other tissues, and longitudinal responsiveness, and may combine *circTspan3* with complementary cartilage markers to enhance performance.

While this study offers critical insights, several limitations merit acknowledgment. First, although the murine model offers valuable proof‐of‐concept, it may not fully capture the complexity and heterogeneity of human cartilage pathophysiology. Second, although we demonstrated *circTspan3*’s role in suppressing ferroptosis and apoptosis, its precise molecular targets and interacting partners remain to be identified. While bioinformatics analysis identified 12 miRNAs predicted to interact with both *circTspan3* and ANXA2, we did not experimentally validate these miRNA interactions. Our study prioritized direct biochemical identification and functional characterization of the *circTspan3*–ANXA2 protein complex, which we demonstrate is necessary and sufficient for *circTspan3*'s chondroprotective effects. Whether specific miRNAs modulate this axis—for instance, by fine‐tuning ANXA2 expression in response to mechanical loading or inflammatory cues—remains an important question for future investigation. Such studies could employ luciferase reporter assays, miRNA mimic/inhibitor experiments, and developmental expression profiling to determine if miRNAs provide context‐dependent regulation of the *circTspan3*–ANXA2 pathway in distinct cartilage pathologies.

Third, we did not perform serial in vivo sampling to directly couple release/degradation kinetics with histological repair. Our new in vitro enzymatic data (collagenase/hyaluronidase) narrow the gap between PBS testing and the physiological joint environment, and future work may integrate noninvasive in vivo imaging or synovial fluid sampling for pharmacokinetic–pharmacodynamic alignment. Additionally, our in vivo data were generated in an acute injury setting, which captures traumatic cartilage damage but not the chronic, low‐grade inflammatory milieu of osteoarthritis. Because exosome biodistribution, joint clearance, and cell‐type targets may differ in chronically degenerated joints, future work should evaluate *circTspan3*‐EV dosing in longitudinal OA models (e.g., DMM/ACLT, aging/metabolic OA) using repeat intra‐articular administration, extended follow‐up, and endpoints encompassing OARSI histology, osteophytes, synovitis, and pain/gait behavior. These studies will define pharmacokinetics/retention and durability in aged cartilage and address the safety of repeated dosing, thereby refining clinical translation.

Despite the promising therapeutic potential of exosomal *circTspan3*, several challenges remain for its clinical translation. First, the scalability of exosome production is a major concern. Efficient isolation and enrichment of exosomes from large quantities of cells or biofluids are crucial for clinical applications, and current methods may not yet be optimized for large‐scale production. Second, the standardization of exosome preparations is vital to ensure reproducibility and consistency across clinical trials. Variability in exosome composition, size, and cargo content can impact therapeutic outcomes. Third, regulatory considerations must be addressed, including compliance with safety and quality control guidelines for exosome‐based therapies. Regulatory agencies may require extensive preclinical studies to evaluate the safety, pharmacokinetics, and immunogenicity of exosome‐based treatments before clinical approval.

## 5 Conclusion

Our findings position *circTspan3* as a systematically XBP1s‐regulated circular RNA that exerts potent anabolic and cytoprotective effects on cartilage through coordinated intracellular signaling and paracrine exosomal delivery. We uncover an ANXA2–mediated circRNA trafficking pathway, offering fresh insight into RNA dynamics and export mechanisms in skeletal tissues. Finally, the successful application of a *circTspan3*‐enriched exosomal hydrogel for in vivo cartilage repair underscores the translational promise of circRNA‐based, cell‐free therapeutics. Collectively, our work lays the groundwork for next‐generation regenerative strategies targeting growth plate abnormalities and degenerative cartilage diseases, with broad implications for orthopaedic precision medicine.

## Author Contributions

All authors contributed to the study conception and design. F.J.G designed the research and supervised the project. Y.M.P. executed all experiments. Y.M.P., J.Y.Z., and Q.G. were responsible for sample collection. YMP and NNG performed statistical analysis of data. F.M.Z., M.Y., Q.Q.Z, Q.M.L., C.L.Z., D.P.Z., Y.D., M.N., and Z.B.W. provided experimental technical support. C.C., Y.D., B.K., and M.T.F. provided validation and methodological support. Y.M.P and F.J.G wrote the manuscript. All authors read and approved the final manuscript.

## Funding

This work was supported by the National Natural Science Foundation of China (No. 82472422, 82272550, 82202760), Chongqing Science and Technology Bureau Foundation (China) (No. CSTB2024NSCQMSX0243, CSTB2023NSCQ‐MSX0288, CSTB2024NSCQ‐MSX0328), CQMU Program for Youth Innovation in Future Medicine (W0146), and Foundation of State Key Laboratory of Ultrasound in Medicine and Engineering (No.2024KFKT017).

## Ethics Statement

All animal studies were performed in accordance with institutional guidelines and approval by the Ethics Committee of Chongqing Medical University (IACUC‐CQMU‐2023‐0121).

## Conflicts of Interest

The authors declare no conflicts of interest.

## Supporting information




**Supporting File 1**: advs73532‐sup‐0001‐SuppMat.docx.

**Supporting File 2**: advs73532‐sup‐0002‐Data.zip.

## Data Availability

The data that support the findings of this study are available from the corresponding author upon reasonable request.
