# Supplementary material for: CircTspan3 Promotes Cartilage Development Through ANNEXIN A2‐Mediated Ferroptosis and Apoptosis Inhibition and Exosome‐Mediated Paracrine Signaling
Source: Adv Sci (Weinh). 2026 Jan 4;13(10):e13418. doi: 10.1002/advs.202513418 (PMC12915206; doi:10.1002/advs.202513418)
Supplement: Supplementary file 1 — Supporting File 1: advs73532‐sup‐0001‐SuppMat.docx. [file ADVS-13-e13418-s002.docx]

***CircTspan3* Promotes Cartilage Development through ANNEXIN A2-mediated Ferroptosis and Apoptosis Inhibition and Exosome-mediated Paracrine Signaling**

Yiming Pan^1^, Fengmei Zhang^1^, Jiayan Zhong^1^, Qian Gong^1^, Nana Geng^1^, Biao Kuang^2^, Qiumei Lan^1^, Miao Yi^1^, Qiqi Zeng^1^, Cheng Chen^3^, Mengtian Fan^1^, Chunliang Zhao^4^, Deping Zeng^4^, Yu Du^2^, Mao Nie^2^, Zhibiao Wang^4^, Fengjin Guo^1^**^*^**

*^1^Department of Orthopedics, State Key Laboratory of Ultrasound in Medicine and Engineering, School of Basic Medical Sciences, The Second Affiliated Hospital of Chongqing Medical University, Chongqing Medical University, Chongqing, China.*

*^2^Department of Orthopedics, The Second Affiliated Hospital of Chongqing Medical University, Chongqing, China.*

*^3^Department of Orthopedics, The First Affiliated Hospital of Chongqing Medical University, Chongqing, China*

*^4^State Key Laboratory of Ultrasound in Medicine and Engineering, Chongqing Medical University, Chongqing, China.*

**Corresponding Author**: Fengjin Guo, Department of Orthopedics, State Key Laboratory of Ultrasound in Medicine and Engineering, School of Basic Medical Sciences, The Second Affiliated Hospital of Chongqing Medical University, Chongqing Medical University, Chongqing 400016, China. E-mail: guo.fengjin@cqmu.edu.cn;

**Keywords**: *circTspan3*, exosomes; *Xbp1*s; Cartilage development; Annexin A2; P-Annexin A2 (Ser26); Apoptosis; Ferroptosis

**Materials and methods**

**Mice**

The primers for determine the genotype of the transgenic mice were as follows:

*Xbp1*^flox/flox^-F: 5'-TGG CAA GGC TGA GCCTGA TCG-3';

*Xbp1^f^*^lox/flox^-R: 5'-GGA ACTAGAGATACCACTGAG-3'

*Col2*Cre-F: 5'- GAGGGTCCAGCCCGAGCTACTT-3',

*Col2*Cre-R: 5'-GCATCGACCGGTAATGCA GGC-3'.

All animal research was conducted in accordance with the guidelines of Chongqing Medical University's Institutional Animal Care and Use Committee. Genotyping of littermates of *Xbp1* ^flox/flox^ *Col2*Cre ^+^ (cKO) mice and control mice (*Xbp1* ^flox/flox^ *Col2*Cre-) was performed as previously described^[1-3]^. Cartilage tissue was collected from three pairs of littermates (*Xbp1* ^flox/flox^ *Col2*Cre ^+^ and ^-^) at embryonic day 18 (E18), 1 week, 2 weeks, and 3 weeks after birth for further research.

**Cell Cultures**

The chondrogenic cell line ATDC5 (Passage 5-13, P5-13) was used as a cell model to evaluate *in vitro* chondrogenesis ^[4]^. Mouse ATDC5 immortalized chondrocytes were purchased from the Chinese Academy of Sciences (Shanghai) and cultured in DMEM/F12 medium containing 5% fetal bovine serum (FBS) and 1% penicillin-streptomycin (Gibco; Thermo Fisher Scientific, Waltham, MA, USA) at 37 °C and 5% CO_2_. ATDC5 cells were treated with insulin-transferrin-selenium (ITS) (Gibco) for 14 days before further experimental treatment.

**RNase R Resistance Assay**

For samples requiring linear RNA removal, total RNA (2 mg) was treated with or without 3 U/mg RNase R (RNR07250; Epicentre Technologies-Illumina, San Diego, CA, USA) at 37 °C for 20 min. RT-qPCR was then performed. The relative expression of *circTspan3* or *Tspan3* was quantified using the comparative CT method (2 ^-ΔΔCt^ ) and normalized to *Gapdh*.

**RNA immunoprecipitation (RIP–qPCR) of the p-ANXA2(Ser26)**

CircTspan3 complex was performed under native conditions using five predefined groups in parallel. Primary mouse chondrocytes (P1) (70–90% confluence; 5 biological replicates per group) were lysed on ice for 10–15 min in RIP lysis buffer supplemented with protease inhibitor and RNase inhibitor (1 U/μL; omitted only in the RNase-treated group below). Lysates were cleared (12,000 × g, 10 min, 4 °C) and 10% was reserved as Input. Protein A/G magnetic beads were pre-blocked (1% BSA + 0.5 mg/mL yeast tRNA, 30 min, 4 °C) and coupled to either anti-phospho-ANXA2(Ser26) antibody or normal IgG (2–5 μg antibody with 30 μL beads, 1 h, 4 °C). Equal volumes of cleared lysate were incubated with antibody-bead complexes for overnight at 4 °C with rotation, followed by four washes on ice. On-beads nuclease challenges defined the five groups: (1) Input (saved before IP for %Input normalization); (2) IgG-IP (−RNase) negative control; (3) p-ANXA2(Ser26)-IP (−RNase) to assess association; (4) p-ANXA2(Ser26)-IP + RNase: on-beads treatment with RNase A (final 100 μg/mL) plus RNase T1 (final 1,000 U/mL) for 15 min at room temperature (no RNase inhibitor), followed by two immediate washes in standard wash buffer; and (5) p-ANXA2(Ser26)-IP + DNase: on-beads treatment with RNase-free DNase I (final 10 U/mL) in manufacturer’s DNase buffer containing 10 mM MgCl₂for 20 min at 37 °C, then two washes in buffer containing 10 mM EDTA to chelate Mg²⁺ and terminate DNase, after which RNase inhibitor was restored. RNA was extracted directly from beads (TRIzol) alongside proportionally processed Input. RIP signals were reported as %Input.

**Terminal deoxynucleotidyl transferase deoxyuridine triphosphate nick end labeling (TUNEL) staining assay**

TUNEL staining was performed using the colorimetric TUNEL apoptosis detection kit (Beyotime Biotech, Shanghai, China) according to the manufacturer's instructions. Briefly, cells were fixed with 4% paraformaldehyde, and washing three times with PBS, then incubated with the TUNEL reaction mixture in a humidified chamber at 37 °C for 2 h. DAPI was used to stain the nuclei.

**Nanoparticle Tracking Analysis (NTA)**

Exosome size distribution and concentration were determined using NanoSight NS300 instrument (Malvern Panalytical, UK) equipped with a 488 nm laser and NTA 3.4 software. Exosome samples were diluted 1:1000 in particle-free PBS to achieve optimal particle concentration (20-100 particles per frame). Five 60-second videos were recorded per sample at camera level 14, with manual temperature monitoring at 25°C. Data analysis was performed using NTA software with detection threshold set at 5^[5]^.

**Immunofluorescence**

For immunofluorescence (IF) evaluation, ATDC5 cells were plated and cultured on slides in six-well plates. When cells reached 10% confluence, various treatments were initiated. Subsequently, slides were fixed with 4% paraformaldehyde for 48 h at room temperature and then immersed in PBS (pH 7.4) and washed three times for 5 min each. Autofluorescence quencher was added to the sections and allowed to stand for 5 min, followed by blocking with blocking buffer (Cell Signaling Technology) for 30 min at room temperature. Subsequently, slides were incubated with primary antibodies in a humidified chamber at 4°C overnight. Anti-AGGRECAN or anti-P-ANNEXIN A2 (Ser26) primary antibodies were used at a dilution of 1:100. The next day, slides were washed with PBS and incubated with recombinant Alexa Fluor 555 anti-mannose-6-phosphate receptor antibody (ab203438) for 50 min at room temperature in the dark. Subsequently, the slides were washed with PBS and incubated with DAPI solution for 5 minutes at room temperature in the dark to stain the cell nuclei. After the last PBS wash, the samples were air-dried and sealed with an anti-fluorescence quencher sheet. The antibodies used for IF were the same as those for Western Blot.

**Transwell co-culture**

This study used the Transwell (Corning, New York, NY, USA, 0.4 µm) co-culture system. The cells were divided into 2 groups. ATDC5 cells overexpressing *circTspan3* were cultured in the upper layer of each group, and wild-type ATDC5 cells were planted in the lower layer. GW4869 (Sigma-Aldrich, St. Louis, MO, USA, 10 μM) was added to the culture medium in group 1, and an equal amount of DMSO was added to group 2. The cells were co-cultured for 7 days, and then RT-qPCR experiments or ELISA were performed.

**RNA extraction and RT-qPCR**

The collected exosomes from cells, frozen tissues, and cell culture medium were added to Trizol (Invitrogen, USA), and RNA was extracted using RNAExpress Total RNA Kit (NCM Biotech, China). Reverse transcription was performed using Superscript II reverse transcriptase (Invitrogen, USA). RT-qPCR was performed using BlastaqTM 2X qPCR MasterMix (ABM, Jiangsu, China) and CFX96 Touch Real Time PCR Detection System (Bio Rad, Hercules, CA, USA). The internal references for circRNA and mRNA were U6 and Gapdh, respectively. Exosomal RNA Isolation Kit (58000, Norgen Biotek) were used for CircRNA extraction from EVs. The relative expression levels were analyzed using the 2^-ΔΔCt^ method. The RT-qPCR primer sequences in our work are shown in Table S3.

**Exosome Isolation and *circTspan3* Detection from Blood**

Blood samples (1 mL) were collected from *Xbp1*cKO and Flox control mice through the retro-orbital method. The collected blood was immediately processed to obtain serum. First, the blood was allowed to coagulate at room temperature for 30 minutes. The serum was then separated by centrifugation at 3,000 g for 10 minutes at 4°C to remove cells and debris. The supernatant (serum) was collected for further processing. To further clarify the serum, it was subjected to an additional centrifugation at 10,000 g for 10 minutes to remove any residual particulate matter.

To isolate exosomes, Exosome Isolation and Purification Kit (from plasma and serum) were used (HY-K1063, MCE). The serum was mixed with PBS and Blood PureExo Solution (BPS). The serum-BPS mixture was incubated at 4°C for 2 hours to allow exosome precipitation. The mixture was then centrifuged at 10,000 g for 60 minutes at 4°C, and the exosome pellet was collected. The exosome pellet was resuspended in PBS and purified using an Exosome Purification Filter (EPF) to remove any remaining impurities. The purified exosome fraction was then collected for subsequent RNA extraction.

Exosomal RNA was extracted from the purified exosome fraction using a standard RNA isolation kit according to the manufacturer's protocol. The RNA was then subjected to quantitative PCR (qPCR) to detect *circTspan3*. Specific primers for *circTspan3* were used in the qPCR assay to identify and quantify the presence of *circTspan3* in the exosome-enriched fraction. The qPCR results were analyzed to confirm the presence and relative abundance of *circTspan3* in the blood-derived exosomes.

**Western Blot Analysis of Exosome Markers**

Exosome lysates were prepared by adding RIPA lysis buffer (Cell Signaling Technology, 9806S) supplemented with protease inhibitor cocktail (Roche, 11873580001). Equal amounts of protein (20 μg) were resolved by SDS-PAGE and transferred to PVDF membranes (Millipore, IPVH00010). Membranes were blocked with 5% non-fat milk in TBST for 1 hour and incubated overnight at 4°C with primary antibodies: anti-CD9 , anti-TSG101, and anti-GRP94 as negative control for cellular contamination^[5]^. After washing, membranes were incubated with HRP-conjugated secondary antibodies (1:5000, Cell Signaling Technology, 7074S or 7076S) for 1 hour at room temperature. Protein bands were visualized using enhanced chemiluminescence substrate (Pierce, 32106) and imaged with ChemiDoc MP imaging system (Bio-Rad, 12003154).

**Lentivirus infection**

The luciferase-tagged lentivirus (KD-*circTspan3*) and its negative control (KD-NC) for stable knockdown of *circTspan3* were constructed by Shanghai Genetech Gene Engineering Technology Co., Ltd. CAL-51 cells were infected with a multiplicity of infection (IM) of 100 and screened with puromycin (2 μg/mL, Solarbio, China). RT-qPCR was used to detect the knockdown efficiency of *circTspan3*.

**Isolation and characterization of exosomes**

To collect exosomes, ATDC5 cells overexpressing *circTspan3* at 80% confluence were cultured in complete medium for exosome collection for 12 hours. The supernatant was then collected and transferred to ultracentrifuge tubes (Beckman Coulter, Brea, CA, USA). The samples were centrifuged at 3000× g for 10 min to remove microvesicles. The supernatant was carefully collected and centrifuged at 120,000× g for 2.5 h at 4 °C. The exosome pellet was reconstituted in PBS and stored at −80 °C.

Exosome concentrations were measured using a bicinchoninic acid (BCA) protein assay kit (CWBioTech, Beijing, China). Exosomal markers were detected by Western blotting. The morphology of exosomes was evaluated using transmission electron microscopy (TEM; JEOL, Tokyo, Japan). Briefly, exosomes were loaded onto copper grids. Exosomes were examined by transmission electron microscopy (TEM) after staining with 2% (w/v) phosphotungstic acid for 5 min. Size distribution was determined by nanoparticle tracking analysis (NTA) using a NanoSight NS300 instrument (Malvern, Worcestershire, UK).

**Exosome Dose–Response Optimization**

To determine the optimal particle-defined dose of *circTspan3*-enriched exosomes, we performed a controlled dose–response titration experiment using primary mouse chondrocytes. Briefly, *circTspan3* expression was first knocked down by lentiviral transduction to establish a *circTspan3*-deficient baseline. Exosomes were isolated from conditioned media using differential ultracentrifugation and purified through a 0.22 µm filter. All exosome preparations were pooled into a single batch and quantified by nanoparticle tracking analysis (NTA) to ensure uniform particle size distribution and concentration.

For dose titration, cells were treated with three defined exosome particle doses:

D1 = 1 × 10⁸, D2 = 3 × 10⁸, and D3 = 1 × 10⁹ particles/mL, corresponding to low, intermediate, and high dose levels, respectively. To verify that biological effects reflected true dose dependency rather than variation in RNA payload loading, matched aliquots of each dose were subjected to RT-qPCR quantification of *circTspan3* using the same number of particles. A *circTspan3*-deficient exosome vector was used as a negative control at the same total particle number as Dose 3.

After 48 hours of incubation, chondrocyte anabolic and stress-response endpoints were assessed. *Col2a1* expression was measured by RT-qPCR as an index of cartilage anabolism. Apoptosis was evaluated using Annexin-V/propidium iodide (PI) staining followed by flow cytometry. Ferroptosis activity was determined by quantifying glutathione peroxidase-4 (GPX4) enzymatic activity using a commercial assay kit.

**Construction of ANXA2 Expression Plasmids**

The mouse *Anxa2* coding sequence (GenBank accession: NM_007585.3) was amplified from mouse ATDC5 cDNA using high-fidelity PrimeSTAR HS DNA Polymerase (Takara R010A) with the following primers: forward,

5'-CGCGGATCCATGAGCATGGCAAATCAA-3' (BamHI site underlined); reverse, 5'-CCGCTCGAGTCATCCTCCACACAGTTC-3' (XhoI site underlined). The PCR product was digested with BamHI and XhoI (New England Biolabs) and ligated into pcDNA3.1(+) vector (Invitrogen V79020) containing an N-terminal FLAG tag (sequence: DYKDDDDK). The resulting plasmid was designated FLAG-ANXA2(WT).

To generate the Ser26-to-Ala point mutant (S26A), site-directed mutagenesis was performed using the QuikChange II Site-Directed Mutagenesis Kit (Agilent 200523) with the following primers:

forward, 5'-GGAAGAGGATCACGCCATCCCTGTTGTCAAG-3';

reverse, 5'-CTTGACAACAGGGATGGCGTGATCCTCTTCC-3' (mutated codon in bold). The mutagenesis reaction contained 50 ng template (FLAG-ANXA2(WT)), 125 ng each primer, 1× reaction buffer, 1 μL dNTP mix, and 2.5 U PfuUltra HF DNA polymerase in a total volume of 50 μL. Thermal cycling conditions were: 95°C for 30 seconds; 18 cycles of 95°C for 30 seconds, 55°C for 1 minute, 68°C for 6 minutes; final extension at 68°C for 10 minutes. The PCR product was treated with 10 U DpnI (New England Biolabs R0176) at 37°C for 1 hour to digest parental (methylated) template DNA, then transformed into DH5α competent cells.

To generate the ΔSer26 deletion mutant (ΔS26), overlap extension PCR was performed using the following primers: Fragment 1 forward,

5'-CGCGGATCCATGAGCATGGCAAATCAA-3'; Fragment 1 reverse,

5'-GATCCTCTTCCGGATCCCTGTTGTCAAG-3' (deletion site in bold); Fragment 2 forward, 5'-CTTGACAACAGGGATCCGGAAGAGGATC-3' (deletion site in bold); Fragment 2 reverse, 5'-CCGCTCGAGTCATCCTCCACACAGTTC-3'. Two separate PCR reactions were performed to generate overlapping fragments, which were then purified using a DNA Gel Extraction Kit (Omega D2500) and mixed in equimolar ratios for the final overlap extension PCR. The final product was digested and cloned into pcDNA3.1(+)-FLAG vector as described above.

All constructs were verified by Sanger sequencing (BGI Genomics, Shenzhen, China) across the entire coding region to confirm the intended mutations and exclude unintended secondary mutations. Plasmids were prepared using EndoFree Maxi Plasmid Kit (Qiagen 12362) to remove endotoxin contamination for mammalian cell transfection. Plasmid concentration was measured using a NanoDrop 2000 spectrophotometer (Thermo Scientific), and purity was confirmed by A260/A280 ratio >1.8 and A260/A230 ratio >2.0.

**Exosome Isolation and Nanoparticle Tracking Analysis**

Exosomes were isolated from conditioned medium using differential ultracentrifugation according to established protocols. ATDC5 cells were cultured in 150 mm dishes (Corning 430599) and transfected as described. Twenty-four hours before exosome collection, culture medium was replaced with DMEM/F12 containing 10% exosome-depleted FBS. Exosome-depleted FBS was prepared by ultracentrifugation of commercial FBS at 100,000 × g for 16 hours at 4°C (Beckman Coulter Optima L-100 XP ultracentrifuge, Type 70 Ti rotor) to remove bovine exosomes, and the supernatant was collected and filtered through a 0.22 μm filter (Millipore SLGP033RS).

Conditioned medium was collected 48 hours post-transfection (approximately 30 mL per dish from 80-90% confluent cells) and subjected to sequential centrifugation steps to remove cells, debris, and large vesicles: 300 × g for 10 minutes at 4°C (to pellet floating cells), 2,000 × g for 20 minutes at 4°C (to remove dead cells and debris), and 10,000 × g for 30 minutes at 4°C (to remove microvesicles). The resulting supernatant was filtered through a 0.22 μm PES syringe filter (Millipore SLGP033RS) to remove any remaining large particles. Exosomes were pelleted by ultracentrifugation at 100,000 × g for 70 minutes at 4°C in a Type 70 Ti rotor. The supernatant was carefully removed, and the pellet was resuspended in 200 μL sterile PBS (filtered through 0.22 μm filter), followed by a second ultracentrifugation wash at 100,000 × g for 70 minutes at 4°C. The final exosome pellet was resuspended in 100-200 μL sterile PBS and stored at -80°C in low-binding tubes (Eppendorf LoBind 022431021) until analysis.

Nanoparticle tracking analysis (NTA) was performed using a NanoSight NS300 instrument (Malvern Panalytical, UK) equipped with a 488 nm laser and sCMOS camera. Exosome samples were diluted 1:100 to 1:1000 in particle-free PBS (filtered through 0.02 μm Anotop filters, Whatman 6809-1002) to achieve a concentration of 20-100 particles per frame (optimal for NTA analysis). Diluted samples (1 mL) were loaded into the sample chamber using a sterile 1 mL syringe. Five 60-second videos were recorded per sample at camera level 14 with manual shutter and gain settings optimized for consistent particle detection. Videos were captured at 25 frames per second. Analysis was performed using NTA software (version 3.4) with detection threshold set to 5 and screen gain set to 10. Particle concentration (particles/mL) and size distribution were automatically calculated by the software based on Brownian motion tracking. To account for dilution factors, the final concentration was backcalculated and expressed as particles per mL of original conditioned medium.

**Exosomal RNA Extraction and Quantification**

RNA was extracted from isolated exosomes using the exoRNeasy Serum/Plasma Kit (Qiagen 77044), which is specifically optimized for low-input vesicular RNA extraction. Exosome pellets (from ultracentrifugation) were resuspended in 500 μL QIAzol Lysis Reagent and vortexed for 5 seconds. After incubation at room temperature for 5 minutes, 100 μL chloroform was added, and tubes were shaken vigorously for 15 seconds. The mixture was incubated at room temperature for 3 minutes and centrifuged at 12,000 × g for 15 minutes at 4°C to achieve phase separation. The upper aqueous phase (approximately 250 μL) was carefully transferred to a new tube without disturbing the interphase. An equal volume of 70% ethanol (250 μL) was added and mixed thoroughly by pipetting. The sample was loaded onto an exoRNeasy spin column and centrifuged at 8,000 × g for 15 seconds. The flow-through was discarded, and the column was washed with 700 μL Buffer RWT followed by two washes with 500 μL Buffer RPE according to the kit protocol.On-column DNase I digestion was performed to eliminate genomic DNA contamination. Briefly, 80 μL DNase I digestion mix (10 μL DNase I stock + 70 μL Buffer RDD, Qiagen 79254) was applied directly to the spin column membrane and incubated at room temperature for 15 minutes. The column was then washed with Buffer RWT (700 μL) and Buffer RPE (2× 500 μL) as above. After a final dry spin at 16,000 × g for 1 minute to remove residual ethanol, RNA was eluted in 14 μL RNase-free water by centrifugation at 8,000 × g for 1 minute.Due to the low concentration of exosomal RNA, reverse transcription was performed using the entire eluate (10 μL) with PrimeScript RT Reagent Kit (Takara RR047A) in a 20 μL reaction volume. To quantify absolute circTspan3 copy numbers, a standard curve was generated using synthetic *circTspan3* (606 nucleotides, corresponding to exons 2-6 junction, synthesized and PAGE-purified by Sangon Biotech, Shanghai, China). Serial dilutions of synthetic *circTspan3* (10⁷ to 10² copies/μL) were reverse transcribed and amplified alongside exosome-derived cDNA samples. RT-qPCR was performed in triplicate for each sample using divergent primers specific for the back-splice junction. The absolute copy number of circTspan3 in each exosome sample was determined by interpolation from the standard curve. To normalize for exosome quantity, *circTspan3* copy numbers were divided by the total exosome particle count (measured by NTA) and expressed as relative *circTspan3* copies per 10⁹ exosome particles.

**Flow Cytometry Analysis**

**Apoptosis Detection**

Apoptotic cells were detected using FITC Annexin V Apoptosis Detection Kit I (BD Biosciences, 556547) according to manufacturer's protocol. Briefly, cells were harvested by trypsinization, washed twice with cold PBS, and resuspended in 1× Annexin V Binding Buffer at a concentration of 1×10^6^ cells/mL. For each sample, 100 μL of cell suspension (1×10^5^ cells) was transferred to a 5 mL culture tube, and 5 μL of FITC Annexin V and 5 μL of propidium iodide (PI) were added. Cells were gently vortexed and incubated for 15 min at room temperature in the dark. After adding 400 μL of 1× Binding Buffer, samples were analyzed within 1 hour using a BD FACSCelesta flow cytometer (BD Biosciences). Data acquisition and analysis were performed using FlowJo software v10.8 (BD Biosciences). At least 6,000 events were collected per sample. Early apoptotic cells were identified as Annexin V+/PI- population, late apoptotic cells as Annexin V+/PI+ population, and viable cells as Annexin V-/PI- population.

**Intracellular Fe**^2+^ **Detection**

Intracellular ferrous iron (Fe^2+^) was measured using FerroOrange probe (Dojindo, F374). Cells were washed twice with HBSS (Gibco, 14025092) and incubated with 1 μM FerroOrange in HBSS for 30 min at 37°C in the dark. After incubation, cells were washed once with HBSS, trypsinized, and resuspended in HBSS for immediate flow cytometric analysis. FerroOrange fluorescence was detected using PE channel (excitation: 543 nm, emission: 580 nm). Mean fluorescence intensity (MFI) was quantified using FlowJo software. At least 10,000 events were collected per sample.

**Chondrogenic Marker Detection**

For intracellular staining of COL2, SOX9, AGGRECAN, and COL10, cells were fixed with 4% paraformaldehyde for 15 min at room temperature, permeabilized with 0.1% Triton X-100 (Sigma-Aldrich, T8787) in PBS for 10 min, and blocked with 5% BSA (Sigma-Aldrich, A7906) for 30 min. Cells were then incubated with primary antibodies overnight at 4°C: anti-COL2A1, anti-AGGRECAN, or anti-COL10A1. After washing, cells were incubated with Alexa Fluor 488-conjugated secondary antibody for 1 hour at room temperature in the dark. Cells were washed, resuspended in PBS, and analyzed by flow cytometry using FITC channel. Unstained and isotype control samples were included in each experiment.

**Biochemical Assays**

**Caspase-3 Activity Assay**

Caspase-3 enzymatic activity was measured using Caspase-3 Colorimetric Activity Assay Kit (Sigma-Aldrich, CASP3C-1KT) according to manufacturer's protocol[9]. Cells were lysed in cold lysis buffer provided in the kit and incubated on ice for 15 min. After centrifugation at 16,000 × g for 10 min at 4°C, supernatants were collected and protein concentration was determined using BCA Protein Assay Kit (Pierce, 23225). Equal amounts of protein (100 μg) were incubated with 10 μL of 4 mM DEVD-pNA substrate (Ac-Asp-Glu-Val-Asp-p-nitroanilide) in assay buffer at 37°C for 2 hours. Absorbance was measured at 405 nm using a SpectraMax M5 microplate reader (Molecular Devices). Caspase-3 activity was calculated as pmol pNA released per minute per mg protein using a pNA standard curve.

**Glutathione Peroxidase 4 (GPX4) Activity Assay**

GPX4 activity was determined using Cellular Glutathione Peroxidase Assay Kit (Cayman Chemical, 703102) with modifications for GPX4 specificity. Briefly, cells were homogenized in cold assay buffer (50 mM Tris-HCl pH 7.5, 5 mM EDTA, 1 mM DTT) and centrifuged at 10,000 × g for 15 min at 4°C. The assay mixture contained 50 μL of sample, 50 μL of co-substrate mixture (NADPH and glutathione reductase), and 20 μL of cumene hydroperoxide (substrate specific for GPX4) in a final volume of 200 μL. The decrease in NADPH absorbance was monitored at 340 nm for 5 min at 25°C. GPX4 activity was calculated as nmol NADPH oxidized per minute per mg protein.

**GSH/GSSG Ratio Measurement**

Reduced glutathione (GSH) and oxidized glutathione (GSSG) levels were determined using GSH/GSSG Ratio Detection Assay Kit II (Abcam, ab205811) following manufacturer's instructions. Cells were deproteinized with 5% 5-sulfosalicylic acid solution, vortexed, and incubated on ice for 10 min. After centrifugation at 8,000 × g for 10 min at 4°C, supernatants were collected for analysis. For total glutathione measurement, 10 μL of sample was mixed with 150 μL of assay mixture containing glutathione reductase and DTNB (5,5'-dithiobis-(2-nitrobenzoic acid)). For GSSG measurement, samples were first treated with GSH quencher (2-vinylpyridine) to remove GSH. Absorbance was measured at 412 nm kinetically for 5 min. GSH and GSSG concentrations were calculated from standard curves, and GSH/GSSG ratio was determined.

**Malondialdehyde (MDA) Assay**

Lipid peroxidation was assessed by measuring MDA levels using Lipid Peroxidation (MDA) Assay Kit (Abcam, ab118970). Cell lysates were mixed with thiobarbituric acid (TBA) solution and incubated at 95°C for 60 min. After cooling on ice, samples were centrifuged briefly and transferred to a 96-well plate. Absorbance was measured at 532 nm. MDA concentration was calculated from an MDA standard curve and normalized to protein content.

**Cell Viability Assay**

Cell viability was determined using Cell Counting Kit-8 (CCK-8, Dojindo, CK04) according to manufacturer's protocol. Cells were seeded in 96-well plates at 5,000 cells per well and treated as indicated. At designated time points, 10 μL of CCK-8 solution was added to each well and incubated for 2 hours at 37°C. Absorbance was measured at 450 nm using a microplate reader. Cell viability was calculated as percentage relative to control group.

**Cycloheximide (CHX) Chase Assay**

ATDC5 cells (P5-7) were transfected with *circTspan3*-overexpressing plasmid or empty vector for 48 hours. Cells were then treated with 50 μg/mL cycloheximide (Sigma-Aldrich, C4859) to block new protein synthesis. At indicated time points (0, 2, 4, 8, 12 hours), cells were harvested and lysed for Western blot analysis. ANXA2 protein levels were quantified by densitometry and normalized to GAPDH loading control at each time point. Protein half-life was calculated by fitting the decay curve to a first-order exponential decay model using GraphPad Prism software.

**Proteasome Inhibition Assay**

To assess whether *circTspan3* affects ANXA2 protein synthesis rate, ATDC5 cells were pretreated with 10 μM MG132 proteasome inhibitor (Selleck Chemicals, S2619) for 6 hours to block proteasomal degradation. Cells were then lysed and ANXA2 protein levels were measured by Western blot to evaluate de novo protein synthesis in the presence or absence of *circTspan3* overexpression.

**Labeling and internalization of exosomes**

Purified exosomes were incubated in 4 μM PKH67 (HY-D142, MCE, USA) at 37 °C for 15 min and then ultracentrifuged at 120,000×g for 90 min to remove unbound dye. After washing twice by centrifugation at 120,000× g in PBS, labeled exosomes were resuspended in PBS before use. ATDC5 cells seeded on 24-well glass slides were seeded in 24-well plates and incubated with PKH67-labeled exosomes at 37 °C. Then, the cells were fixed in 4% paraformaldehyde (PFA) for 15 min and permeabilized in PBS solution containing 0.1% Triton-X for 15 min. The samples were then labeled for span3 by FISH and stained with 4′,6-diamidino-2-phenylindole (DAPI; Invitrogen, CA, USA) for 3 min.

**Chitosan Hydrogel Preparation and Characterization**

**Exosomal *circTspan3*-Chitosan (Exo-*circTspan3*-CS) Hydrogel Formulation**

Thermosensitive chitosan hydrogel was prepared using chitosan-β-glycerophosphate system as described by Chenite et al. with modifications^[6]^. Medical-grade chitosan (degree of deacetylation ≥85%, Mw 50-190 kDa, Sigma-Aldrich, 448877) was dissolved in 0.1 M acetic acid solution at 2% (w/v) concentration under magnetic stirring overnight at 4°C. The chitosan solution was neutralized by dropwise addition of 10 M NaOH until pH 6.8-7.0, followed by filtration through 0.45 μm filter for sterilization.

β-glycerophosphate disodium salt hydrate (Sigma-Aldrich, G9422) was dissolved in sterile distilled water at 56% (w/v) concentration. The β-GP solution was added dropwise to chitosan solution at 4°C under magnetic stirring to achieve final concentrations of 1.5% (w/v) chitosan and 8% (w/v) β-GP. Exosomes enriched with *circTspan3* (3 × 10⁸ particles/mL) were mixed into the chitosan-β-GP solution at 4°C. The mixture remained liquid at 4°C but rapidly formed a semi-solid hydrogel upon warming to 37°C within 5-10 minutes due to temperature-triggered sol-gel transition.

**Scanning Electron Microscopy (SEM)**

Hydrogel microstructure was examined by SEM. Hydrogel samples were rapidly frozen in liquid nitrogen, lyophilized using FreeZone freeze dryer (Labconco, 7670520), and fractured to expose internal structure. Samples were sputter-coated with gold using Cressington 108auto sputter coater (Ted Pella) for 60 seconds at 20 mA. Images were acquired using a Zeiss Sigma 300 VP field emission SEM at accelerating voltage of 5 kV.

**Mechanical Testing**

Compression testing was performed using TA.XTplus Texture Analyzer (Stable Micro Systems, UK) equipped with a 5 kg load cell^[7]^. Cylindrical hydrogel samples (10 mm diameter × 10 mm height) were prepared in sterile molds and incubated at 37°C for 30 min to ensure complete gelation. Native mouse articular cartilage explants (same dimensions) were harvested from knee joints as positive controls. Uniaxial unconfined compression was applied at a strain rate of 1%/s up to 30% strain at room temperature. Compression modulus (E) was calculated from the linear region (10-20% strain) of the stress-strain curve using equation E = σ/ε, where σ is compressive stress (kPa) and ε is strain. At least three independent samples per group were tested.

**Adhesion Testing**

Hydrogel adhesive properties were evaluated using a custom adhesion test as described by Radhakrishnan et al.^[8]^. Briefly, 50 μL of pre-warmed hydrogel (37°C) was applied to a latex glove surface (to simulate biological tissue) and allowed to gel for 5 min at 37°C. The gloved finger was repeatedly flexed and extended 50 times, and hydrogel adhesion was visually assessed and photographed.

***In Vitro* Exosome Release Kinetics**

Hydrogel samples (200 μL containing 2×10^9^ exosomes) were prepared in 1.5 mL microcentrifuge tubes and incubated at 37°C with gentle agitation in 1 mL of either: (1) PBS (pH 7.4, Gibco, 10010023) or (2) enzymatic degradation medium containing 0.25% collagenase type II (Sigma-Aldrich, C6885) and 0.1% hyaluronidase (Sigma-Aldrich, H3506) in PBS to simulate intra-articular enzymatic environment.

At predetermined time points (1,7,10 days), 100 μL of supernatant was collected and replaced with fresh medium to maintain sink conditions. Released exosomes were quantified by NTA. Cumulative exosome release was calculated as percentage of total loaded exosomes.

***In Vitro* Hydrogel Degradation**

Hydrogel degradation was measured by monitoring mass loss over time. Lyophilized hydrogel samples (initial dry weight W₀) were incubated at 37°C in PBS or enzymatic medium (as described above) with medium changed every 3 days. At designated time points ( 7, 14, 28 days), samples were retrieved, rinsed with distilled water, lyophilized, and weighed (Wₜ). Residual mass was calculated as (Wₜ/W₀) × 100%.

**Mouse cartilage defect model and treatment**

Adult male mice (8 weeks old) were used for cartilage defect model (n=5). The skin of the mouse knee joint area was incised, and then the incision was made along the medial side of the patellar ligament and through the quadriceps femoris to help dislocate the patella. The patellar groove was exposed, and a 3mm×3mm cylindrical full-thickness cartilage defect was made along the length of the groove on the non-weight-bearing surface using an electric drill. Exosome *circTspan3*-CS hydrogel or exosome Vector-CS hydrogel was injected into the defect site for cartilage repair. The patella was then repositioned, and the joint capsule and skin were sutured, respectively. The contralateral calf served as an uninjured internal control. The mice and rats were killed after 8 weeks for further study. The cartilage samples were fixed with 4% paraformaldehyde, processed, and embedded in paraffin. Serial sections of the cartilage were stained with H&E and safranin O according to standard protocols. The repaired cartilage sections of different groups were subjected to immunohistochemical staining for cartilage anabolism markers (SOX9, AGGRECAN, COL2) according to the standard protocol, and the staining images were taken with an optical microscope. The chondroprotective effect of the scaffold was examined by evaluating the cartilage of the tibial plateau (TP) according to the criteria of the ICRS cartilage injury classification system^[9]^.

**Plasmid construction**

The CDS sequence of gene was queried by NCBI, primer design was performed by primer primer5, and truncator primers were designed. Human or mouse cDNA was utilized as a template, amplified using Tsingke Gold Mix Ver. 2 (Cat. TSE102), and then constructed into pcDNA3.1/myc-His(-)A vector with MYC tag. These components were then submitted to Tsingke for sequencing and identification, followed by endotoxin removal plasmid extraction (Magen, Cat.P1111-02).

*circTspan3* overexpression vector were designed and the mouse full-length *circTspan3*was inserted into vector pLC5-ciR by Geenseed (Guangzhou, China), the mock vector without *circTspan3* sequence was served as a control. To knock down *circTspan3*, siRNAs (small interfering RNAs) targeting back splice junction of *circTspan3* and siRNA-NC were synthesized by Geenseed (Guangzhou, China). The KD-*circTspan3* was subcloned into the lentivirus vector GV344 by Genechem (Shanghai, China). The lentiviral vector was transiently transfected into HEK293T cells. All transfections were implemented using Lipofectamine 3000 (Invitrogen, Carlsbad, CA, USA) according to manufacturer’s instructions. The sequences used were displayed in Supplementary Table 2.

**Stable cell lines and plasmid transfection**

The mouse circTspan3 overexpression vector, the short hairpin (sh) RNA vector targeting circTspan3, and the empty lentiviral vector were purchased from GENESED (Guangzhou, China). After the lentiviral vector was co-transfected with the packaging plasmids psPAX2 and pMD2.G into 293T cells, the supernatants were collected at 48 and 72 hours, respectively, and used to infect ATDC5 cells. After 48 hours, the stably infected cell lines were selected with puromycin according to the manufacturer's recommended protocol.

**RNA sequencing analysis**

RNA was isolated from ATDC5 cells treated with circTspan3 knockdown lentivirus or control lentivirus using TRIzol reagent. RNA sequencing libraries were constructed using NEBNext® Ultra™ RNA library preparation kit and then deep sequenced using Illumina sequencer (HiSeq, Fasteris SA, Switzerland) at Beijing Novogene Technology Co., Ltd. Small RNAs from DPSC-Exo were extracted and used for miRNA sequencing. miRNA libraries were constructed and then deep sequenced using Illumina HiSeq 3000 platform at Guangzhou Ruibo Biotechnology Co., Ltd.

Raw gene count matrices were analyzed using Bioconductor. Quality control of raw sequencing data was performed using FastQC tool. Differentially expressed genes (DEGs) were analyzed using edgeR analysis package in R statistical program with the criteria of adjusted p value ≤ 0.05 and absolute log2 (fold change) > 2. Heatmaps and volcano plots were created using RStudio. Gene ontology (GO) term and Kyoto Encyclopedia of Genes and Genomes (KEGG) enrichment analysis of dysregulated DEGs were performed using the Database for Annotation, Visualization, and Integrated Discovery (DAVID).

**Histological observation**

For hematoxylin-eosin (H&E) staining, 2.5 µm-thick sections were obtained from paraffin-embedded specimens. The specimens were deparaffinized, stained with hematoxylin-eosin reagents (G1120, Solarbio). For toluidine blue staining, tissue sections were deparaffinized, soaked in toluidine blue dye (G3661, Solarbio) at room temperature for 20 minutes. For Saffron-O and Fast Green staining (G1371, Solarbio), the sections were treated with Fast Green Staining Solution for 5 minutes, stained with Saffron-O Staining Solution for 5 minutes. The morphological changes were observed and photographed using a microscope (Olympus, Tokyo, Japan).

At 8 weeks post-treatment, heart, liver, spleen, lung, and kidney were harvested, fixed in 10% neutral buffered formalin (≥24 h), paraffin-embedded, sectioned at 4–5 μm, and stained with hematoxylin–eosin (H&E) following standard protocols. Sections were examined by light microscopy at ×100 and ×400 magnifications. Representative fields were imaged under identical acquisition settings. Qualitative endpoints included parenchymal architecture, necrosis, inflammatory cell infiltration, hemorrhage/congestion, fibrosis, and cytoplasmic vacuolation.

**Immunohistochemistry for collagen type (COL) 2 and COL10**

Cartilage sections were deparaffinized and rehydrated, followed by antigen retrieval in citrate buffer (pH 6.0). Sections were incubated with anti-COL2 or anti-COL10 primary antibodies overnight at 4°C. Secondary antibodies (goat anti-rabbit or goat anti-mouse HRP-conjugated, 1:500, Thermo Fisher) were used for detection. Immunostaining was visualized using DAB substrate, and sections were counterstained with hematoxylin. Quantification of COL2 and COL10 expression was measured using ImageJ software (NIH).

**Molecular docking analysis**

The structure of ANNEXIN A2 was downloaded from the AlphaFold database (https://www.alphafold.ebi.ac.uk). The MFE structure of *circTspan3* was predicted using RNA folding analysis (http://rna.tbi.univie.ac.at/). The 3D model of *circTspan3* was predicted by 3D-RNA (https://biophy.hust.edu.cn/new/3dRNA). The molecular interaction model of *circTspan3*-ANNEXIN A2 was constructed using the HDOCK server (http://hdock.phys.hust.edu.cn/). To evaluate the binding quality of the model, the specific binding energy was predicted and the electrostatic force of the amino acid level interaction at the binding interface was calculated using the Schrödinger software. In brief, the binding interface of *circTspan3* in the model was predicted by 3DRNA and then processed using the Nucleotide Preparation Wizard module of the Schrödinger software. Protein crystals of ANNEXIN A2 were pre-processed using the Protein Preparation Wizard module of Schrodinger software. Molecular docking analysis was performed using the Nucleotide Protein Docking module. The lower the docking score, the lower the binding free energy, indicating a higher binding stability. If the binding energy is <1000 kcalmol ^−1^ , the interaction between nucleic acid and protein were considered to be highly stable. Finally, the binding interface was visualized using PyMOL version 2.50.

**Immunofluorescence (IF)**

The cells were cultured on glass coverslips were incubated in primary antibodies overnight at 4°C. After that, appropriate fluorescent probe-conjugated secondary antibodies were applied for 4 hours at room temperature. DAPI was used to counterstain cell nuclei. The primary antibodies used were Collagen Type II Polyclonal antibody, or Aggrecan Polyclonal antibody, Annexin A2 Polyclonal antibody, Phospho-Annexin A2 (Ser26) Antibody, and Xbp1 Antibody.

**Nuclear-cytoplasmic fractionation**

Total RNA was extracted from tissues and cells using the FreeZol Reagent (R711-01, Vazyme, China). In addition, nuclear and cytoplasmic RNA in cells were isolated using the Cyolasmic & Nuclear RNA Purification Kit (Cat.21000, Norgen, Canada) according to the manufacturer’s protocols. Reverse transcription was conducted using the PrimeScript RT Reagent Kit (Takara, Dalian, China), and qRT-PCR analysis was performed using the TB Green Premix Ex Taq (Takara, Dalian, China).

**Fluorescence in Situ Hybridization (FISH)**

A *circTspan3* probe tagged with Cy3 was created (GENESED, Guangzhou, China). Primary chondrocytes were cultured on coverslips, incubated with FISH probes in hybridization buffer (GENESED, Guangzhou, China), DAPI was used to label the nuclei. To examine the localisation of *circTspan3* in the growth plate, 1W mouse knee joint slices were dewaxed and treated similarly to chondrocytes. These photos were captured using an Olympus BX51 fluorescent microscope (Tokyo, Japan).

**Luciferase reporter assay, and chromatin immunoprecipitation (ChIP)**

Cells were seeded into 24-well plates and then transfected with the luciferase reporter plasmids, including vector of pGL3-basic, positive control of pGL3-control, pGL3-*Tspan3* for 48 h. The luciferase activity was measured for duplicate repeats and analysed by the Dual-Luciferase Reporter Assay System (Promega, Cat. E1910) according to the manufacturer’s instructions.

ChIP experiments were performed using Chromatin Immunoprecipitation (ChIP) Kit (Bes5001, BersinBio^TM^). The primary antibodies used were Xbp1s-specific Polyclonal antibody (1:50), and IgG (1:50).

**Western Blot (WB)**

Protein lysates were separated using SDS-polyacrylamide electrophoresis (SDS-PAGE) gels (Cat. No. P1200; Solarbio). Proteins were transferred to a PVDF membrane (Millipore) after separation, and the membrane was blocked with 5% nonfat milk before being incubated with the Collagen Type II Polyclonal antibody (Cat. No. 28459-1-AP; Proteintech), or Aggrecan Polyclonal antibody (Cat. No. 13880-1-AP; Proteintech), Xbp1s-specific Polyclonal antibody (Cat. No. 24868-1-AP; Proteintech), Annexin A2 Polyclonal antibody (Cat. No. 11256-1-AP; Proteintech), Sox9 Antibody (E-9) (Cat. No. sc-166505; Santa cruz), GAPDH Monoclonal antibody (Cat. No. 60004-1-Ig; Proteintech), Beta Actin Monoclonal antibody (Cat. No. 66009-1-Ig; Proteintech), Phospho-Annexin A2 (Ser26) Antibody (AF5440; Affinity), Secondary HRP–conjugated antibodies (Solarbio) were used. ImageJ software was used to quantify the western blot bands.

**Construction of ANXA2 Ser26 mutants.**

Full-length mouse Anxa2 cDNA (GenBank accession NM_007585.3) was PCR-amplified from mouse chondrocyte cDNA using Phusion High-Fidelity DNA Polymerase (New England Biolabs, M0530S) and cloned into pcDNA3.1(+) expression vector (Thermo Fisher, V79020) with an N-terminal FLAG tag (DYKDDDDK) via EcoRI/XhoI restriction sites. The Ser26→Ala (S26A) point mutation was introduced using QuikChange II Site-Directed Mutagenesis Kit (Agilent Technologies, 200524) with mutagenic primers:

forward, 5'-GAAGAAGATGATCAGCAAGCCATCAAGGAAACGGACAAAGAC-3'; reverse, 5'-GTCTTTGTCCGTTTCCTTGATGGCTTGCTGATCATCTTCTTC-3' (TCC→GCC codon change).

Following PfuUltra HF DNA polymerase-mediated amplification (18 cycles: 95°C 30 s; 95°C 30 s, 55°C 1 min, 68°C 6 min × 18; 68°C 7 min), the methylated parental template was digested with DpnI (37°C, 1 h) and the product transformed into XL1-Blue supercompetent cells. The ΔSer26 (ΔS26) in-frame deletion mutant was generated by overlap extension PCR. Two primary fragments were amplified separately using primer pairs OF (5'-GCGGAATTCATGGACTACAAGGACGACGATGACAAG-3') + IR (5'-GTCTTTGTCCGTTTCCTTGATTTGCTGATCATCTTCTTC-3'), and IF (5'-GAAGAAGATGATCAGCAAATCAAGGAAACGGACAAAGAC-3') + OR (5'-GCGCTCGAGTCATTCATCCCCACCACACAGG-3'), where IF and IR primers contained 18 bp complementary overlap sequences and excluded the Ser26 codon (TCC, nucleotides 76-78). Purified fragments were fused by primer-less PCR (5 cycles: 98°C 30 s; 98°C 10 s, 60°C 30 s, 72°C 30 s × 5), followed by amplification with outer primers OF + OR (25 cycles: 98°C 10 s, 64°C 20 s, 72°C 30 s × 25; 72°C 5 min). The fusion product was digested with EcoRI/XhoI and ligated into pcDNA3.1(+). All constructs were verified by Sanger sequencing of the entire ANXA2 coding region (Eurofins Genomics) and purified using EndoFree Plasmid Maxi Kit (Qiagen, 12362) for transfection. Expression and phosphorylation status were confirmed by Western blot using anti-FLAG M2 (1:1,000), anti-ANXA2 (1:1,000), anti-phospho-ANXA2(Ser26) (1:500;), and anti-Tubulin B (1:5,000) antibodies. Anti-phospho-ANXA2(Ser26) signal was detected only in wild-type but not S26A or ΔS26 constructs, confirming successful ablation of Ser26 phosphorylation, while total ANXA2 expression levels were equivalent across all three constructs when normalized to Tubulin B.

**Supplementary Figures**


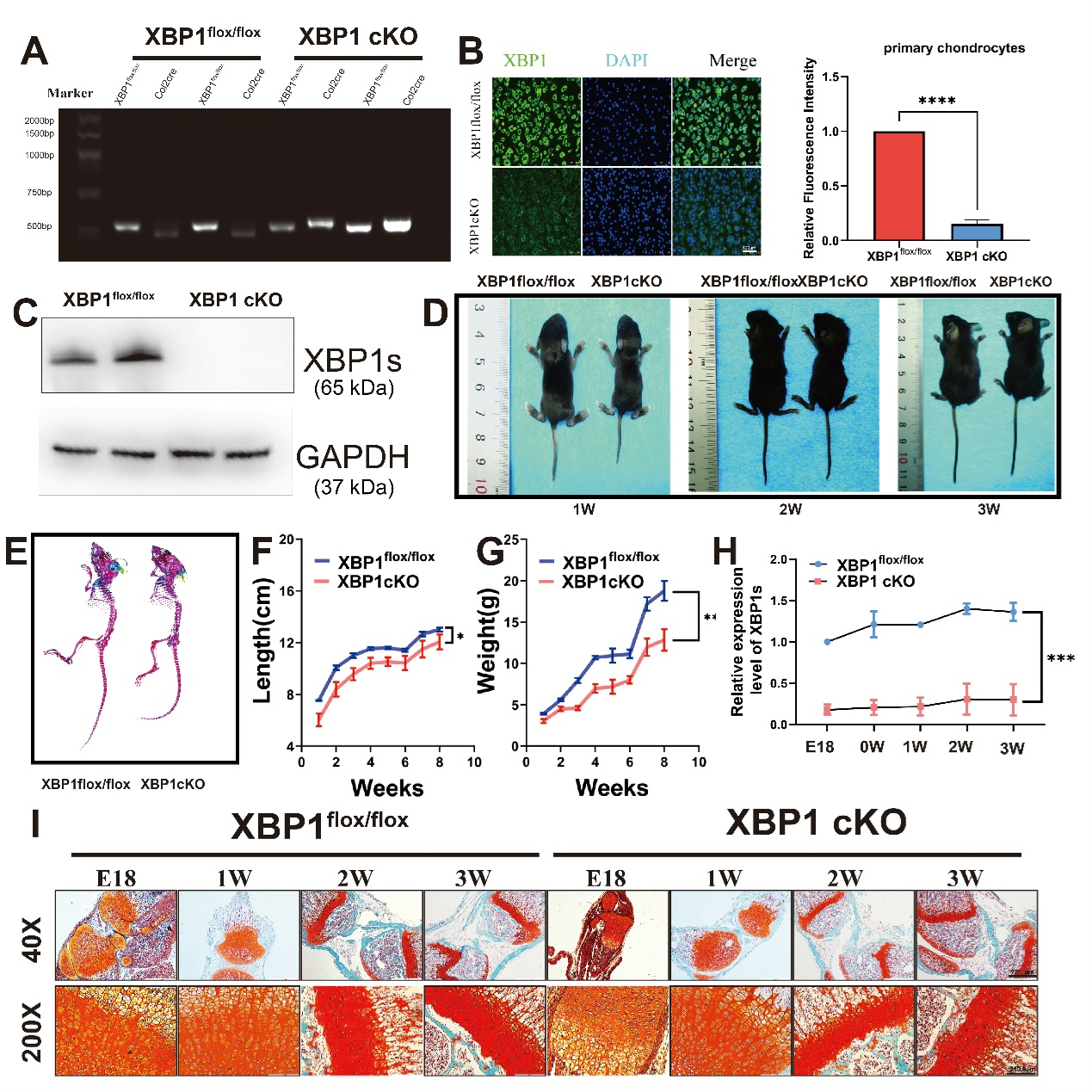


**Figure S1.** Generation and characterization of cartilage-specific *Xbp1* knockout mice.

(A) Representative agarose gel electrophoresis images showing PCR-based genotyping results for *Xbp1*^flox/flox^ and *Xbp1*cKO (*Xbp1*^flox/flox^; *Col2a1*-Cre) mice. Representative gels from n = 6 mice per genotype.

(B) Immunofluorescence (IF) staining confirms efficient deletion of XBP1s protein in primary chondrocytes isolated from *Xbp1*cKO mice compared to *Xbp1*^flox/flox^ controls. Cells were stained with anti-XBP1s antibody (Green) and DAPI (blue, nuclear counterstain). Scale bar: 50 μm. Representative images from n = 3 independent cell isolations per genotype.

(C) Western blot analysis of XBP1s protein levels in primary chondrocytes from *Xbp1*^flox/flox^ and *Xbp1*cKO mice. Total protein lysates (30 μg per lane) were probed with anti-XBP1s antibody and anti-GAPDH antibody.

(D) Representative photographs of 1-week-old *Xbp1^f^*^lox/flox^ and *Xbp1*cKO littermate mice showing visible difference in body size. Mice were photographed on a standardized grid background for size comparison. Representative images from n = 8 mice per genotype.

(E) Skeletal preparations stained with Alcian blue/Alizarin red comparison of *Xbp1* cKO and littermate control *Xbp1*^flox/flox^ mice at 1 week old. Representative images from n = 5 mice per genotype.

(F) Body length(cm) comparison of *Xbp1* cKO and littermate control *Xbp1*^flox/flox^ mice at 1-8 weeks. Measurements were performed weekly at the same time of day to minimize diurnal variation. n = 8 mice per genotype (4 males, 4 females per group; no sex-dependent differences observed). Data presented as mean ± SEM using Paired-Student’s t test.*P<0.05.

(G) Body weight(g) comparison of *Xbp1* cKO and littermate control *Xbp1*^flox/flox^ mice at 1-8 weeks. n = 8 mice per genotype. Data presented as mean ± SEM using Paired-Student’s t test.**P<0.01.

(H) Comparison of *Xbp1s* levels in knee articular cartilage of *Xbp1* cKO and littermate control Xbp1^flox/flox^ mice at embryonic day 18(E18), postnatal 1-3 weeks (n=8). n = 8 mice per genotype per time point. ***P<0.001.

(I) Comparison of Saffron-O and Fast Green staining in knee articular cartilage of *Xbp1* cKO and littermate control *Xbp1*^flox/flox^ mice at E18, postnatal 1-3 weeks.


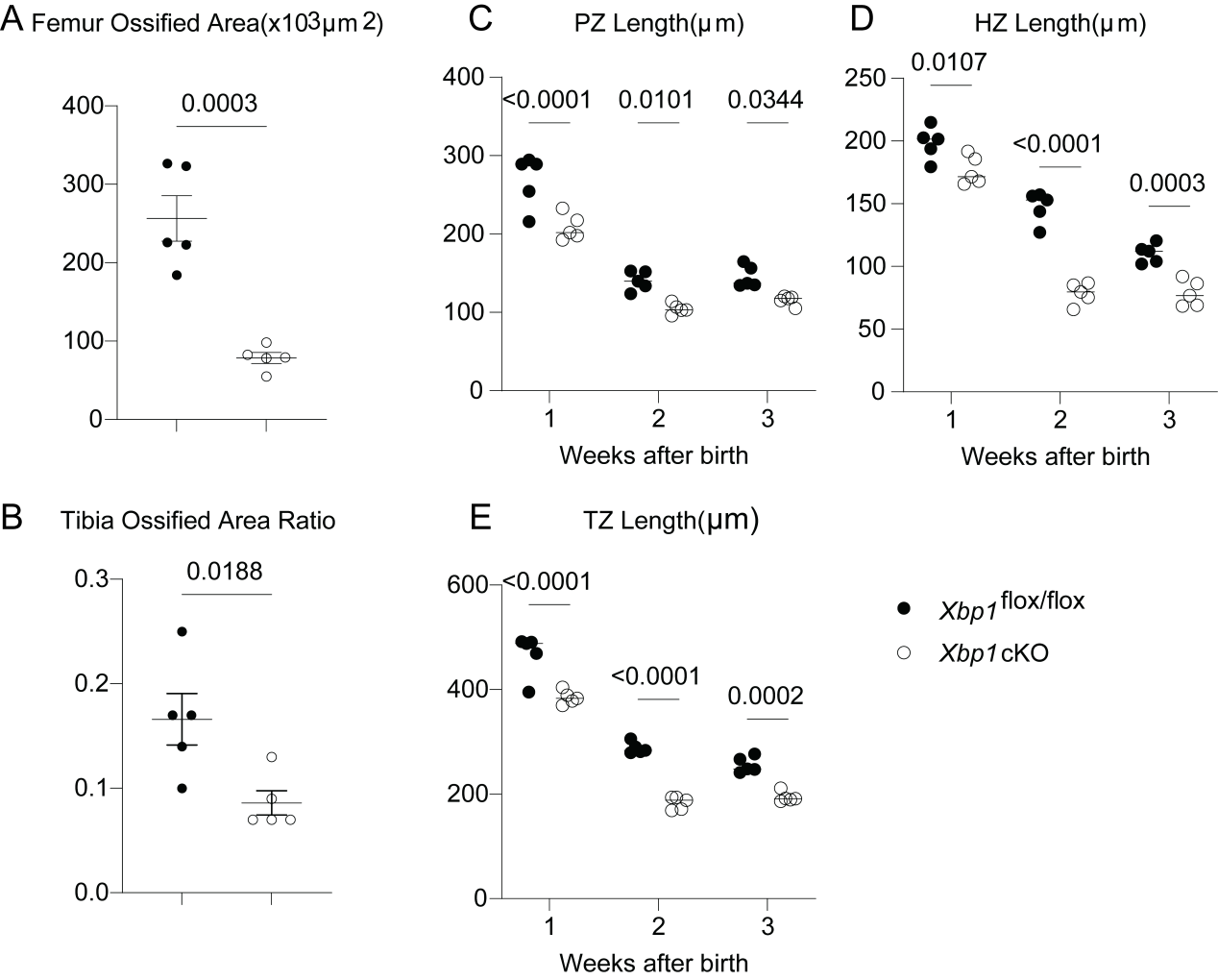


**Figure S2.** Compared with littermates *Xbp1*^flox/flox^ and *Xbp1*cKO, cartilage growth plates in cKO mice are hypoplastic.

1. Quantification of ossified femoral area in *Xbp1*^flox/flox^ and *Xbp1*cKO mice at postnatal day 7 (W1), measured from high-magnification (100×) H&E-stained sections (corresponding to Figure 1C). Ossified bone area was defined as the region with mature bone trabeculae and quantified using ImageJ software by tracing the bone boundary and calculating total area. *Xbp1*cKO mice show significantly reduced ossified area. Unpaired Student's t-test; n = 5 mice per genotype. Data presented as mean ± SEM.
2. Quantification of ossified tibial area in *Xbp1*^flox/flox^ and *Xbp1*cKO mice at W1, measured from the same histological sections as panel A. Unpaired Student's t-test; n = 5 mice per genotype. Data presented as mean ± SEM.

(C) Quantification of proliferative zone (PZ) height in tibial growth plates from *Xbp1*^flox/flox^ and *Xbp1*cKO mice at P7, measured from high-magnification (200×) H&E-stained sections (corresponding to Figure 1E). PZ was defined as the region containing flattened-to-columnar chondrocytes arranged in longitudinal columns, extending from the resting zone to the onset of chondrocyte enlargement. Three measurements per section were taken at evenly spaced intervals across the central growth plate region and averaged. *Xbp1*cKO mice display significantly reduced PZ height. Unpaired Student's t-test; n = 5 mice per genotype. Data presented as mean ± SEM.

(D) Quantification of hypertrophic zone (HZ) height in tibial growth plates from the same sections as panel C. HZ was defined as the region containing enlarged, rounded chondrocytes with abundant cytoplasm, extending from the last columnar cell to the chondro-osseous junction. *Xbp1*cKO mice show significantly shortened HZ. Unpaired Student's t-test; n = 5 mice per genotype. Data presented as mean ± SEM.

(E) Quantification of transitional zone (TZ, also termed pre-hypertrophic zone) height in tibial growth plates from the same sections as panels C-D. TZ was defined as the narrow region between the proliferative and hypertrophic zones containing chondrocytes transitioning from columnar to enlarged morphology. *Xbp1*cKO mice exhibit significantly reduced TZ height. Unpaired Student's t-test; n = 5 mice per genotype. Data presented as mean ± SEM.

All measurements were performed using ImageJ software (version 1.53a, NIH) by two independent observers blinded to genotype, with high inter-rater reliability (Pearson's r > 0.95). For each mouse, three non-consecutive sections spaced ≥50 μm apart were analyzed, and values were averaged to obtain a single biological replicate value. Zone boundaries were determined based on established histomorphometric criteria: RZ (small, round cells with sparse matrix), PZ (flattened cells in longitudinal columns), TZ (chondrocyte enlargement initiation), HZ (maximally enlarged cells), and ossification front (vascular invasion and osteoblast appearance).


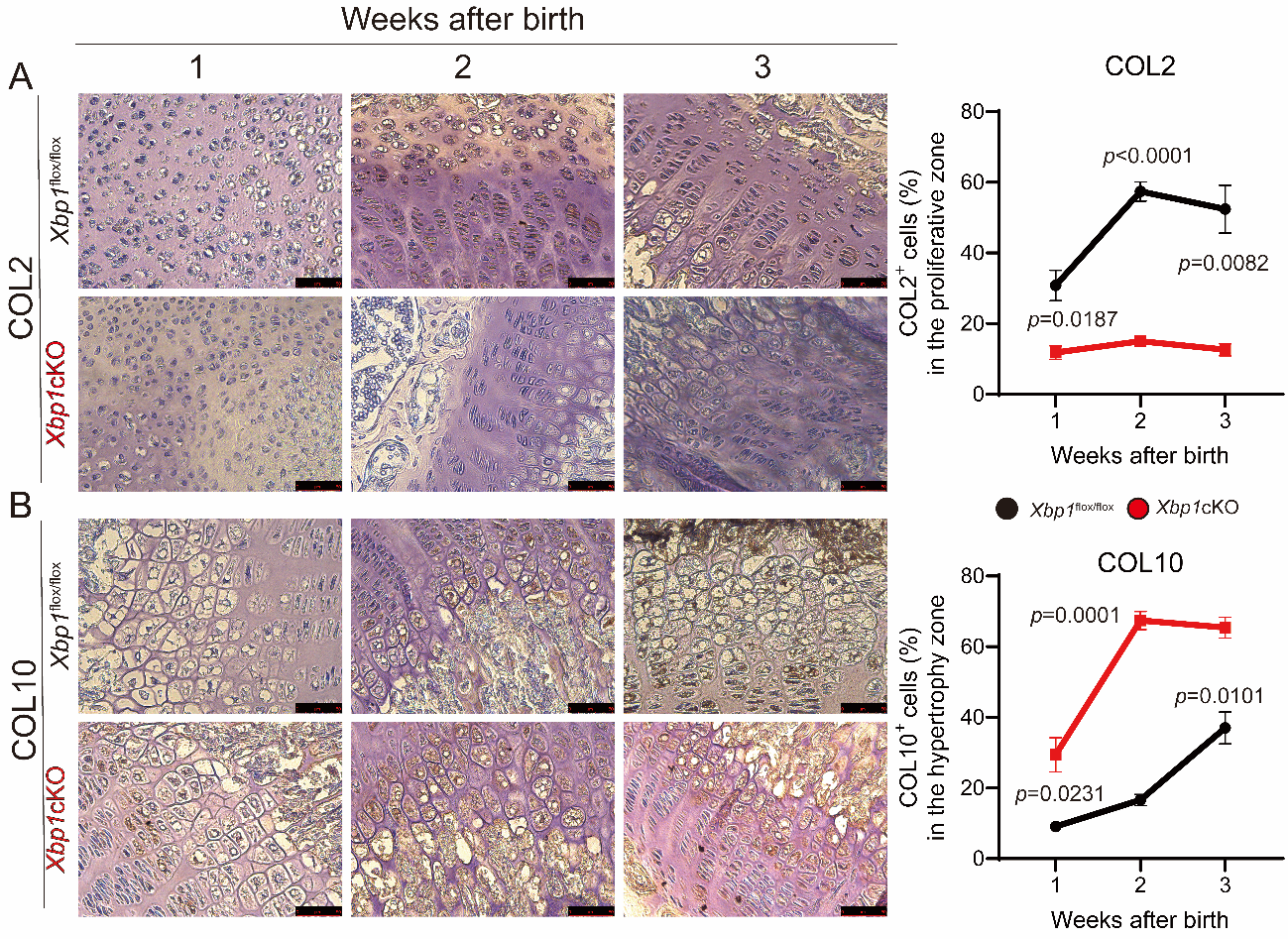


**Figure S3.** Temporal analysis of cartilage matrix protein expression reveals impaired COL2 synthesis and premature COL10 expression in *Xbp1*cKO growth plates.

(A) Representative immunohistochemistry images and quantification of COL2-positive chondrocytes in the proliferative zone (PZ) of tibial growth plates from *Xbp1*^flox/flox^ and *Xbp1*cKO littermates at postnatal day 7 (W1), day 14 (W2), and day 21 (W3). Scale bars: 50 μm. Brown DAB staining indicates COL2-positive cells. Quantification represents percentage of COL2-positive cells within the PZ (total cells counted: 300-500 per section). Two-way ANOVA followed by Tukey's post hoc test; n = 5 biological replicates per genotype per time point. Data represents means ± SEM.

(B) Representative immunohistochemistry images and quantification of COL10-positive chondrocytes in the hypertrophic zone (HZ) of tibial growth plates from littermate mice at W1-3. Scale bars: 50 μm. Quantification represents percentage of COL10-positive cells within the HZ. Two-way ANOVA followed by Tukey's post hoc test; n = 5 biological replicates per genotype per time point. Data represents means ± SEM.


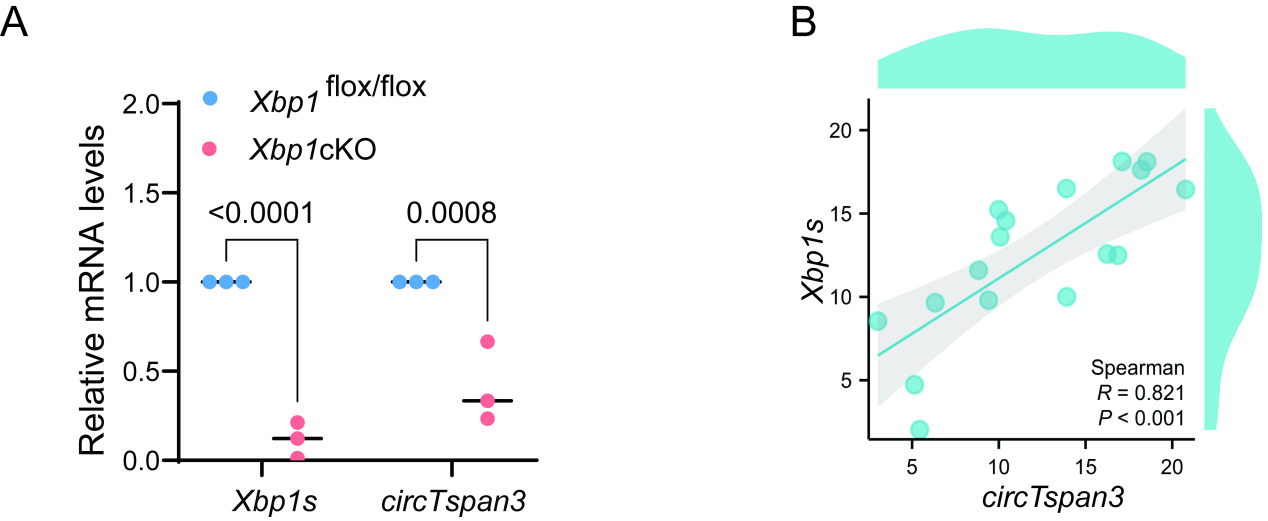


**Figure S4.** *Xbp1s* expression positively correlates with *circTspan3* levels in mouse cartilage

(A)RT-qPCR was used to detect the expression levels of *Xbp1s* and *circTspan3* in cartilage of *Xbp1*^flox/flox^ mice and *Xbp1*cKO mice at postnatal day 7 (W1). Relative expression was calculated using the 2^-ΔΔCt^ method with *Gapdh* as the internal reference gene. Data are expressed as fold change relative to *Xbp1*^flox/flox^ group. Paired Student's t-test was used to compare expression levels between littermate pairs (n = 3 matched pairs of mice). Data presented as mean ± SEM. Each biological replicate represents cartilage pooled from both knees of one individual mouse to obtain sufficient RNA yield.

(B)Pearson correlation analysis showed that *Xbp1s* have positively correlated with *circTspan3*. Pearson correlation analyses were performed using IBM SPSS Statistics 23. n=17.

**
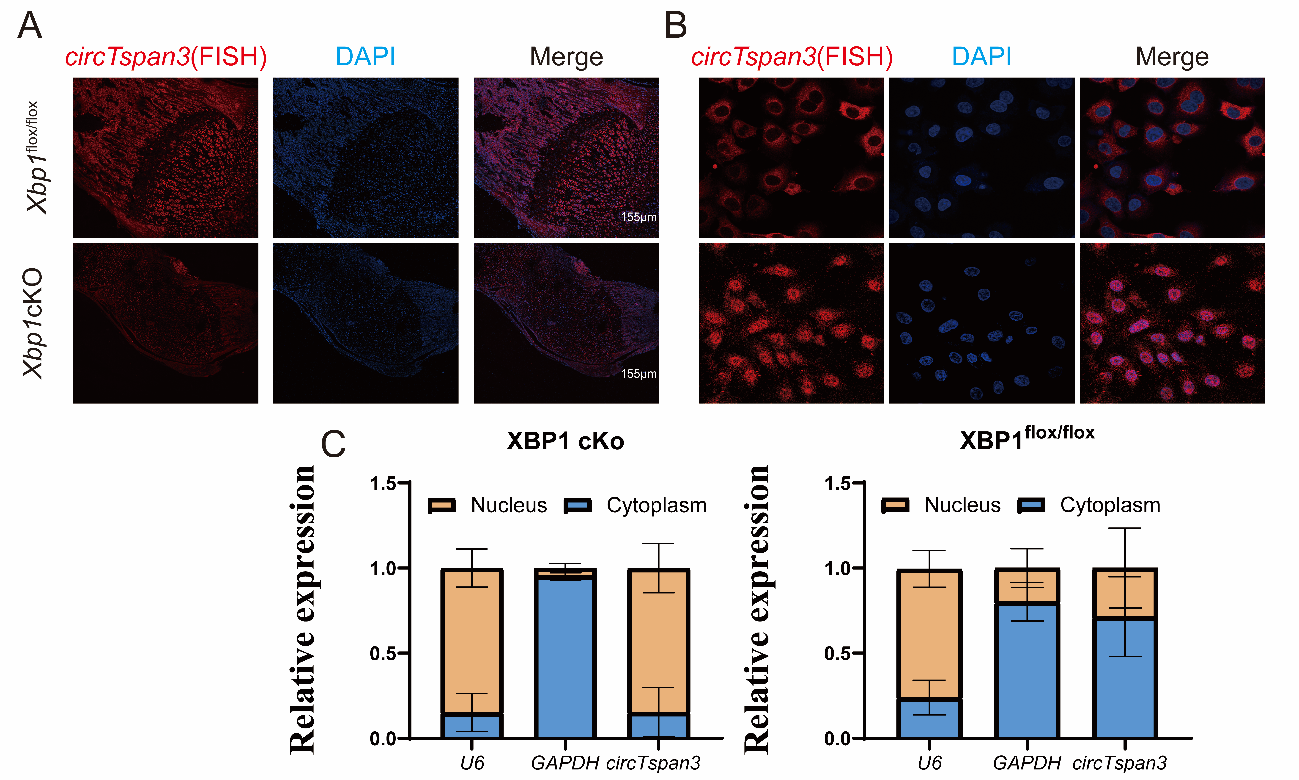
**

**Figure S5.** *CircTspan3* exhibits predominantly cytoplasmic localization with reduced overall abundance in *Xbp1*cKO mice compared to littermate controls.

(A)Representative fluorescence in situ hybridization (FISH) images showing *circTspan3* subcellular localization in tibial growth plate sections from *Xbp1*^flox/flox^ and *Xbp1*cKO littermate mice at postnatal day 7. Nuclei were counterstained with DAPI (blue). Representative images from n=3 mice per genotype. Scale bar: 155 μm.

(B)Representative FISH images showing *circTspan3* subcellular localization in primary chondrocytes (Passage 1, P1) isolated from *Xbp1*^flox/flox^ and *Xbp1*cKO littermate mice. Processed for FISH as described in panel A. Scale bar: 50 μm. Representative images from n=3 independent primary cell isolations per genotype.

(C)Nuclear-cytoplasmic fractionation followed by RT-qPCR analysis of *circTspan3* subcellular distribution in primary chondrocytes (P1) from *Xbp1* cKO and littermate control *Xbp1*^flox/flox^ mice. *U6* was considered as nuclear control and *Gapdh* was used as a cytoplasmic gene control. n=3 biological replicates per genotype. Data presented as mean ± SEM.


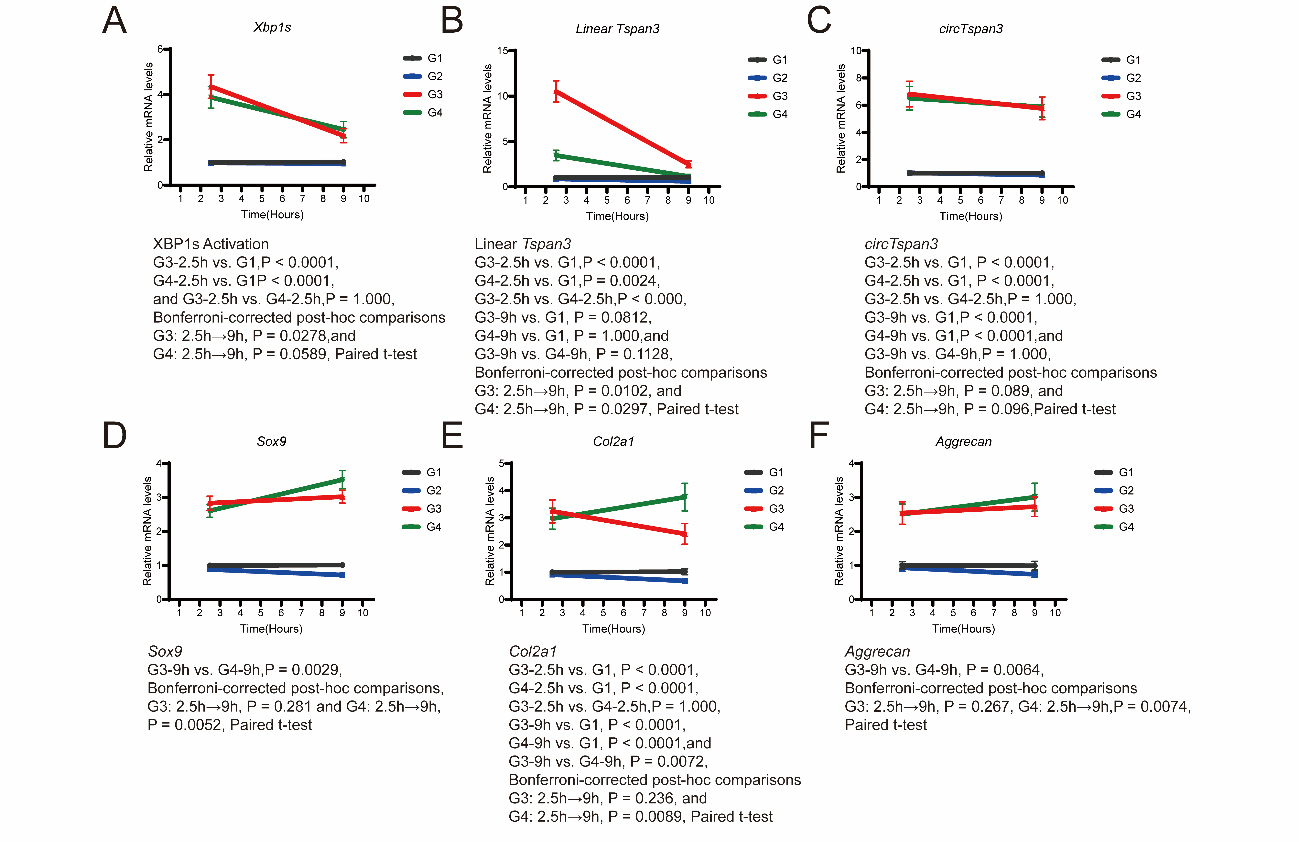


**Figure S6.** Dual-time point pulse-chase experiment reveals differential stability and functional contributions of linear versus circular *Tspan3*.

Primary mouse chondrocytes (P2) were treated with vehicle (G1), ActD alone (G2), tunicamycin pulse (G3), or tunicamycin pulse followed by ActD (G4), and sampled at 2.5h and 9h. Data are means ± SEM; n=3 independent biological replicates. Statistical analyses: Two-way ANOVA with Bonferroni post-hoc test for inter-group comparisons; paired t-test for within-group temporal comparisons.

(A) *XBP1s* mRNA expression levels measured by RT-qPCR at 2.5h and 9h in all four groups.

(B)Linear *Tspan3* mRNA expression levels quantified using convergent primers spanning canonical exon-exon junctions at both time points.

(C) *CircTspan3* levels measured using divergent primers specific for the back-splice junction with RNase R-treated RNA templates at both time points.

(D) *Col2a1* mRNA expression levels assessed by RT-qPCR at 2.5h and 9h across all groups.

(E) *Sox9* mRNA expression levels measured by RT-qPCR at both time points.

(F) *Aggrecan* mRNA expression levels quantified by RT-qPCR at 2.5h and 9h.


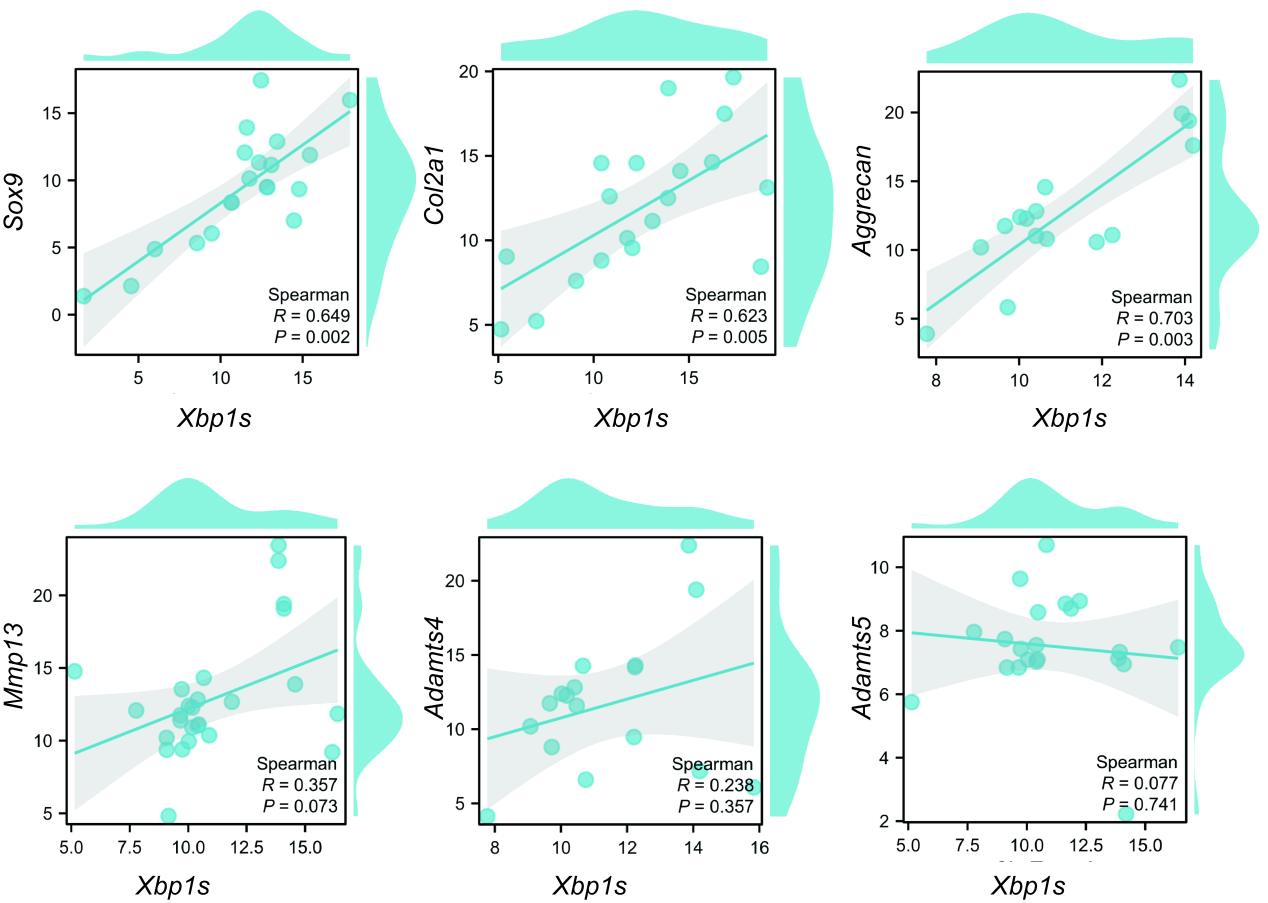


**Figure S7.** Mouse knee growth plate results showed that *Xbp1s* was positively correlated with cartilage synthesis indicators and no correlated with cartilage catabolism indicators.

Pearson correlation analysis between *Xbp1s* mRNA expression levels and cartilage matrix markers in knee growth plate cartilage samples from wild-type C57BL/6 mice across multiple developmental stages (embryonic day 18, postnatal days 7, 14, and 21). Growth plate cartilage was microdissected from the proximal tibial metaphysis. All RT-qPCR reactions were performed in technical triplicates, and relative expression was calculated using the 2^-ΔΔCt^ method with *Gapdh* as the reference gene.

Pearson correlation analysis showed that *Xbp1s* have positively correlated with *Sox9* (n=18), *Col2a1*(n=19), and *Aggrecan*(n=16), and *Xbp1s* have no correlated with *Mmp13* (n=24), *Adamts4* (n=17), and *Adamts5* (n=21). Pearson correlation analyses were performed using IBM SPSS Statistics 23. Each data point represents one biological sample.


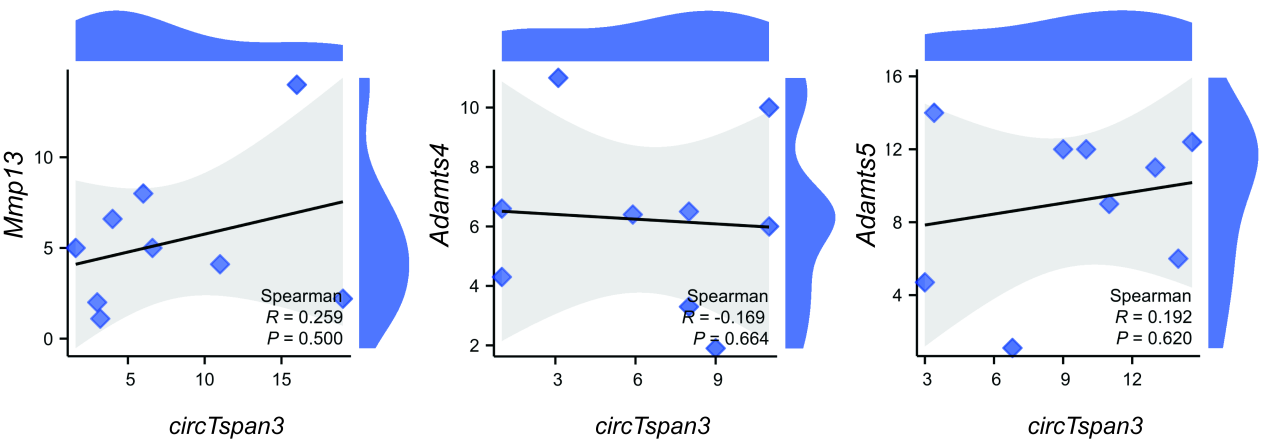


**Figure S8.** *CircTspan3* expression is consistently reduced in *Xbp1*cKO mice but does not correlate with catabolic markers across development.

Pearson correlation analysis between *Xbp1s* mRNA expression levels and cartilage matrix markers in knee growth plate cartilage samples from wild-type C57BL/6 mice across multiple developmental stages (embryonic day 18, postnatal days 7, 14, and 21). Growth plate cartilage was microdissected from the proximal tibial metaphysis. All RT-qPCR reactions were performed in technical triplicates, and relative expression was calculated using the 2^-ΔΔCt^ method with *Gapdh* as the reference gene.

Pearson correlation analysis showed that *circTspan3* have no correlated with *Mmp13* (n=9), *Adamts4* (n=9), and *Adamts5* (n=9). Pearson correlation analyses were performed using IBM SPSS Statistics 23. Each data point represents one biological sample.


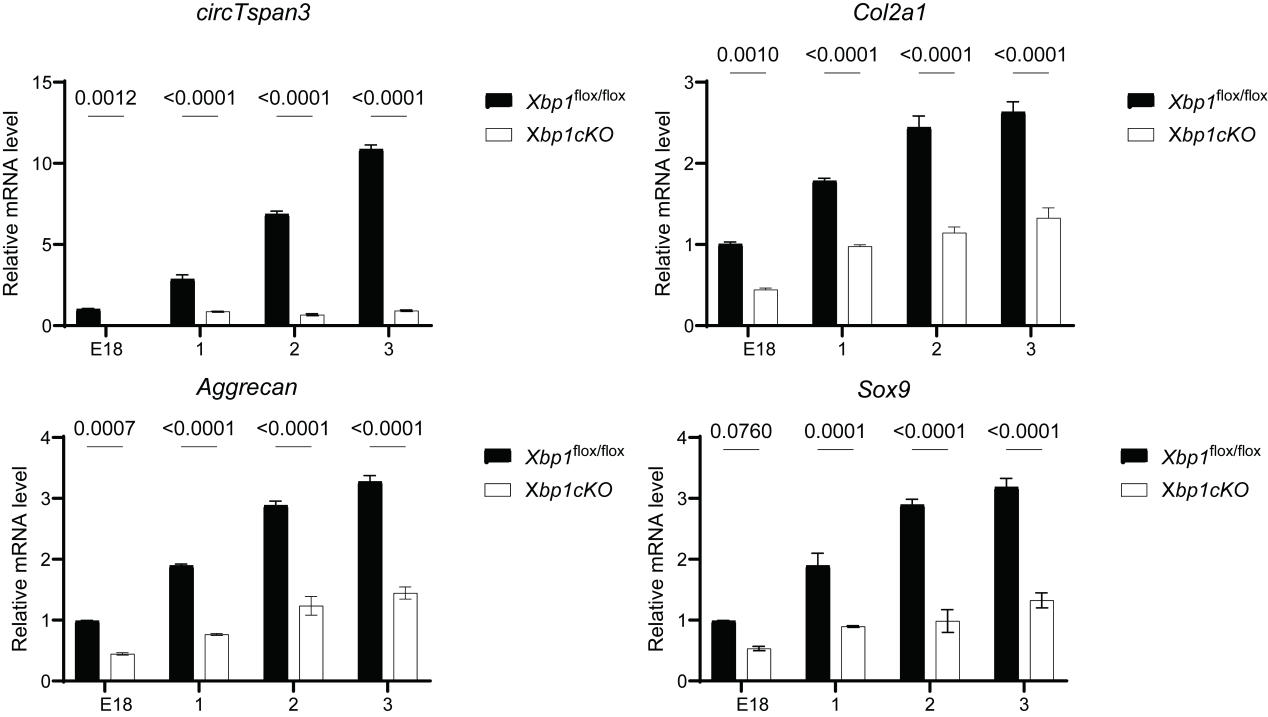


**Figure S9.** Longitudinal expression analysis of *circTspan3* and anabolic markers in *Xbp1*cKO mice.

RT-qPCR time-course analysis of *circTspan3*, *Col2a1*, *Aggrecan*, and *Sox9* expression in knee cartilage from *Xbp1*^flox/flox^ and *Xbp1*cKO littermate mice at four developmental stages: embryonic day 18 (E18), postnatal day 7 (W1), postnatal day 14 (W2), and postnatal day 21 (W3). Cartilage tissue was microdissected, total RNA extracted using TRIzol, and gene expression quantified by RT-qPCR with *Gapdh* normalization. Data expressed as fold change relative to *Xbp1*^flox/flox^ E18 group. Line graphs show temporal trajectory for each genotype. Both genotypes exhibit age-related increases in all anabolic markers, consistent with normal developmental cartilage maturation. However, *Xbp1*cKO mice show significantly lower expression at all time points compared to *Xbp1*^flox/flox^ controls, indicating sustained impairment of anabolic programs. Two-way ANOVA (genotype × time) followed by Tukey's post hoc test. n = 3 mice per genotype per time point. Data presented as mean ± SEM.


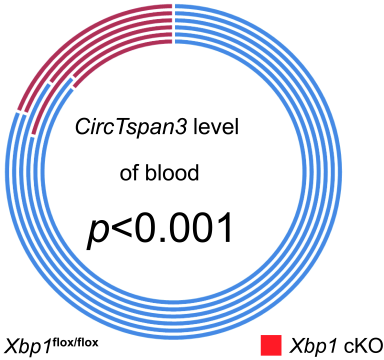


**Figure S10.** *CircTspan3* is detectable in extracellular vesicles (EVs) from peripheral blood of *Xbp1*^flox/flox^ mice. Data are expressed as fold change relative to *Xbp1*^flox/flox^ (set as 1.0), calculated using the 2^-ΔΔCt^ method with spike-in normalization.

Paired Student's t-test (comparing paired littermates, n = 3 matched pairs); Data presented as mean ± SEM. Each biological replicate represents blood EVs from one individual mouse.


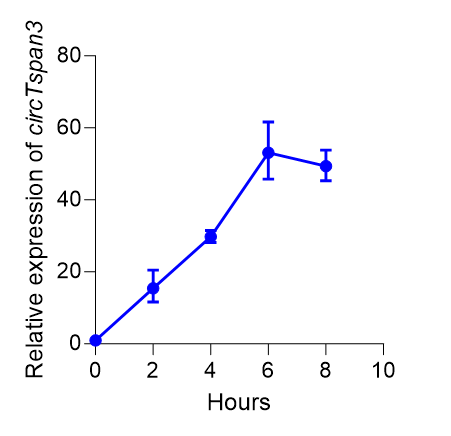


**Figure S11.** Exosomes extracted from ATDC5 cells (Passage 8-11, P8-11) overexpressing *circTspan3* (exosomal c*ircTspan3*) were added to the culture medium of wild-type ATDC5 cells. The expression of *circTspan3* in wild-type ATDC5 cells (P8-11) at different time points was detected by RT-qPCR (n = 3 biological replicates per time point. P values indicated in the figure), Data represents means ± SEM.


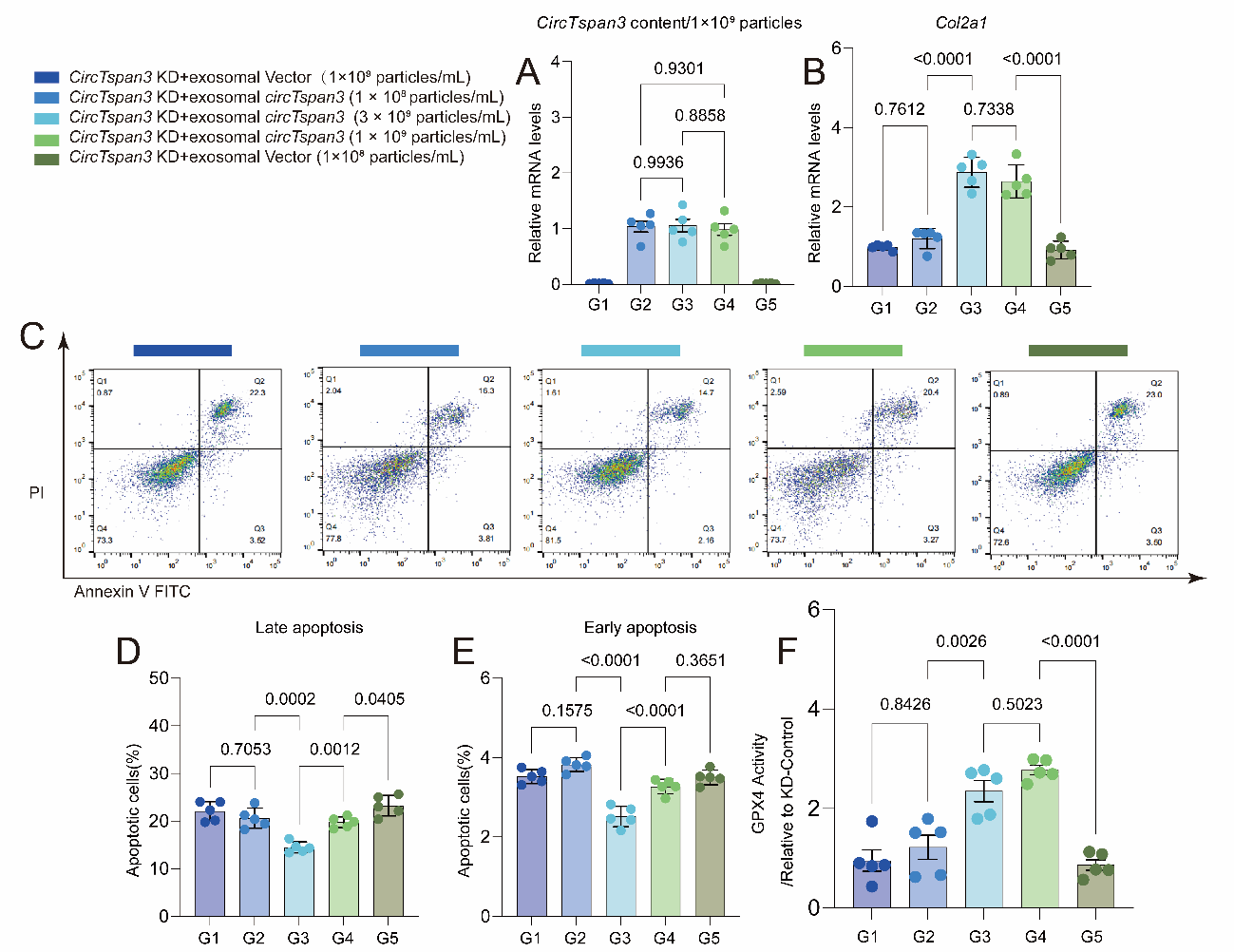


**Figure S12.** Dose titration of *circTspan3*-enriched exosomes in chondrocytes (P8-11).

(A) Quantification of *circTspan3* levels in exosome preparations using RT-qPCR, performed on equal particle numbers from each batch (n = 5). Statistical analysis: one-way ANOVA followed by Tukey’s post-hoc test for pairwise comparisons among the three groups.

(B–F) ATDC5 cells with *circTspan3* knockdown were cultured for 48 h in medium supplemented with increasing concentrations of *circTspan3*-enriched or not exosomes.

(B) RT-qPCR quantification of *Col2a1* mRNA expression.

(C) Representative Annexin V-FITC/PI flow cytometry plots showing apoptosis.

(D–E) Quantification of early and late apoptotic cell populations from panel C.

(F) GPX4 enzymatic activity measured in primary chondrocytes.

All experiments were performed in five independent biological replicates (n = 5 per group). Data are presented as mean ± s.e.m. Statistical analysis: one-way ANOVA with Tukey’s multiple comparison test for normally distributed data or Kruskal–Wallis test with Dunn’s correction for non-parametric data, with pairwise comparisons performed among all groups.


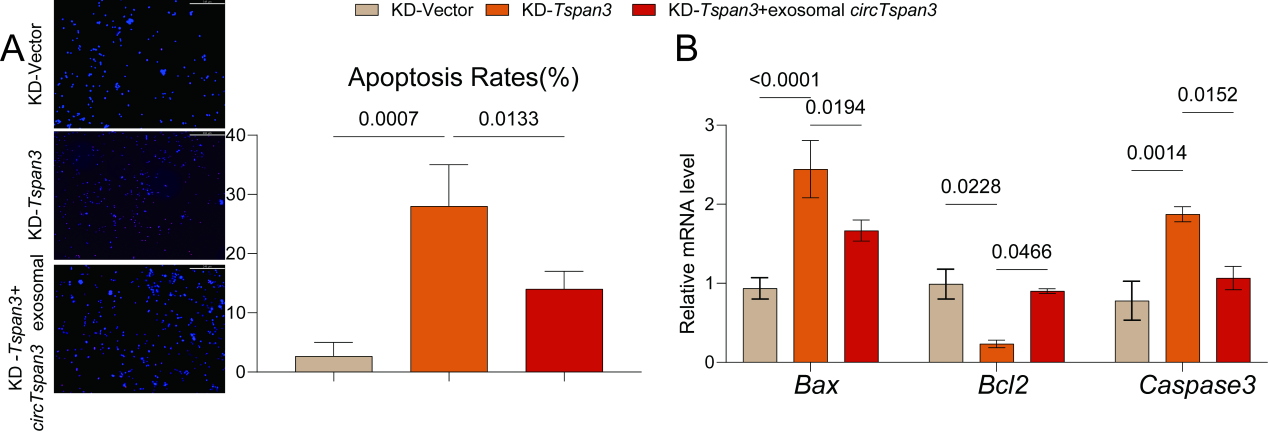


**Figure S13**. Exosomal *circTspan3* alleviated the increased apoptosis level of ATDC5 chondrocytes caused by knockdown of *Tspan3*.

1. Representative images and quantification of TUNEL staining of cells in each group.
2. RT-PCR was used to detect the apoptosis level of cells in each group.

Data represents means ± SEM using ANOVA followed by post hoc test, n = 3 biological replicates per time point. P values indicated in the figure.


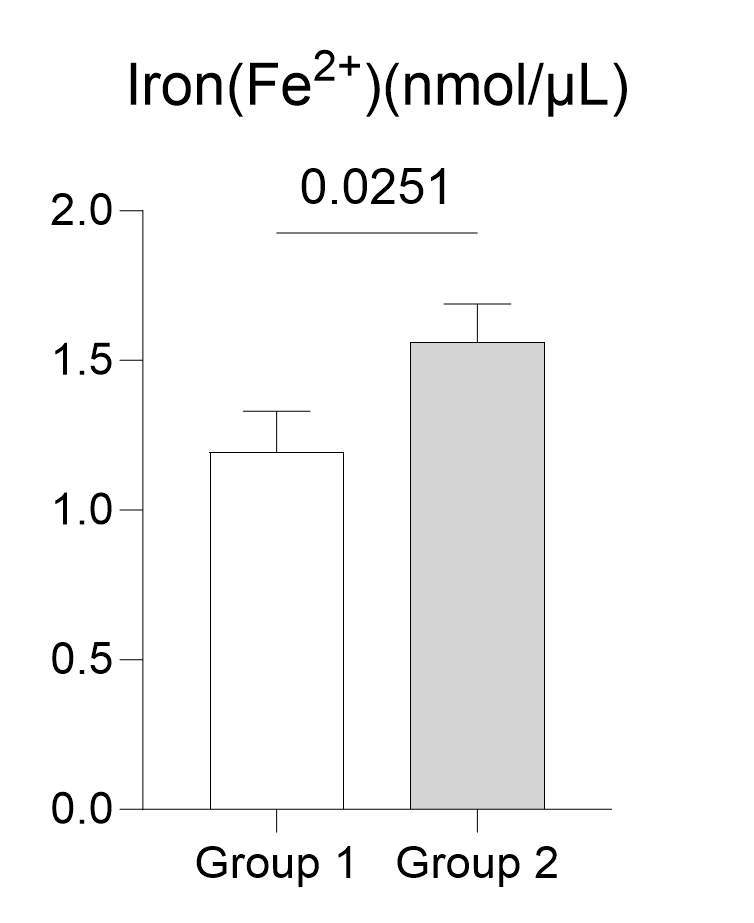


**Figure S14**. The iron ion content of the lower layer cells in the co-culture medium without (Group 1) or with GW4869 (Group 2) was compared. Data represents means ± SEM using Paired-Student’s t test, n = 3 biological replicates per time point. P values indicated in the figure.


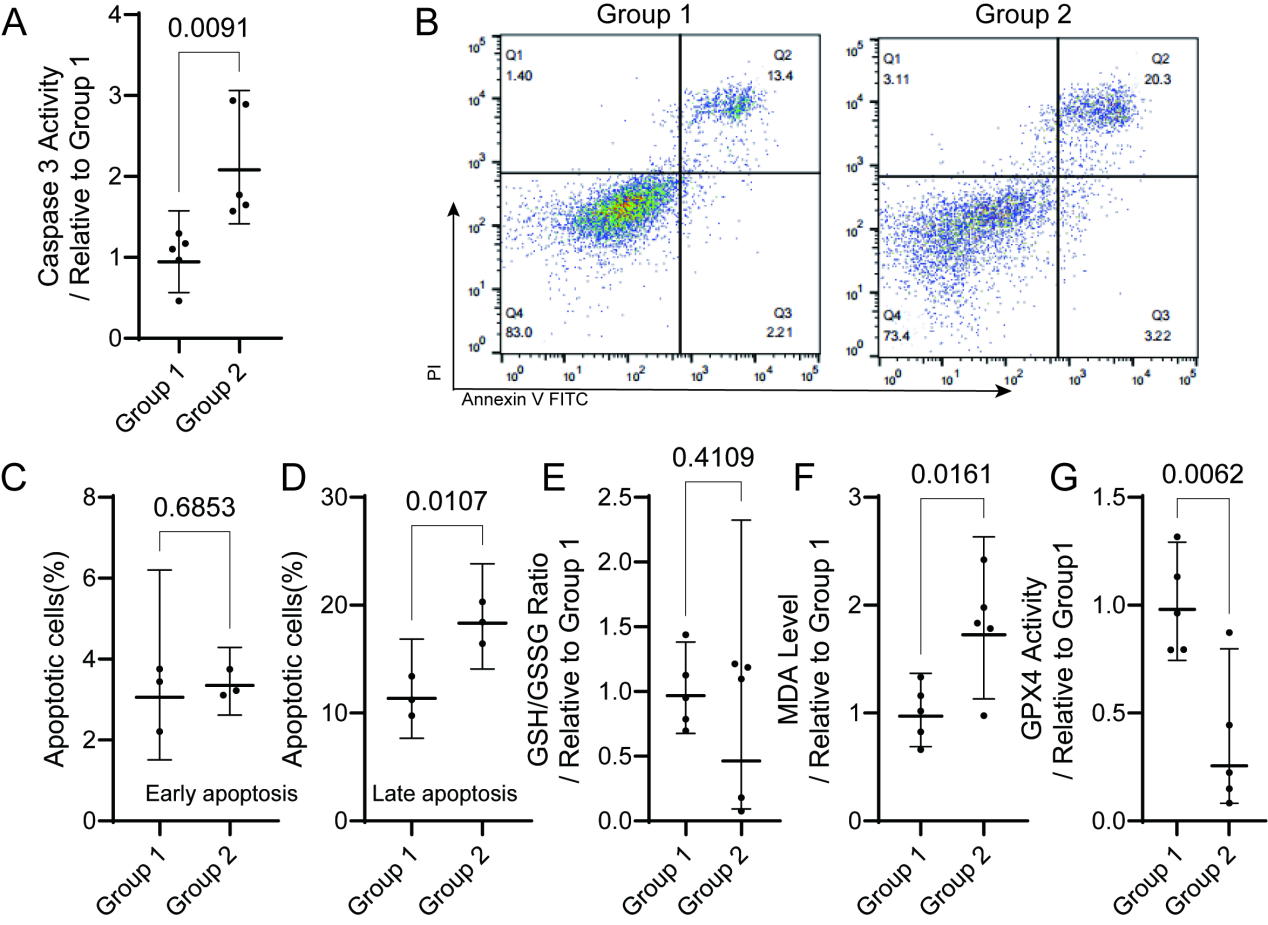


**Figure S15.** The apoptosis/ferroptosis marker of the lower layer cells in the co-culture medium without (Group 1) or with GW4869 (Group 2) was compared. Data represents means ± SEM using Paired-Student’s t test, n = 5 biological replicates per time point. P values indicated in the figure.


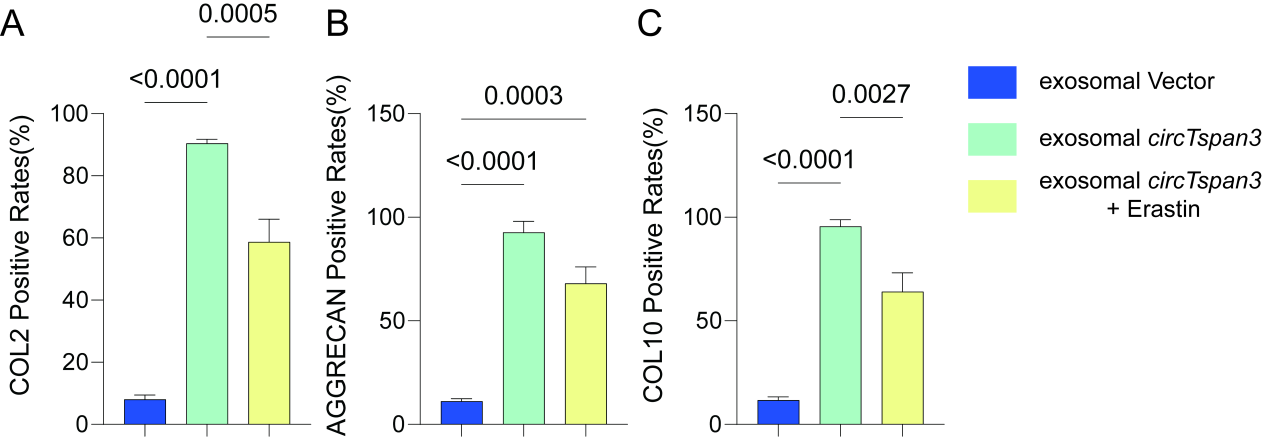


**Figure S16**.Quantification of flow cytometry results of Figures 5J-5M.

(A)Representative flow cytometry histograms showing COL2 expression in ATDC5 cells (Passage 5-7, P5-7) after 21-day chondrogenic differentiation various treatments. Two-way ANOVA (lentivirus treatment × time point) followed by Tukey's post hoc test for each gene. n = 3 biological replicates per time point. P values indicated in the figure.

(B)Representative flow cytometry histograms showing AGGRECAN expression in ATDC5 cells (P5-7) after 21-day chondrogenic differentiation with various treatments. Two-way ANOVA (lentivirus treatment × time point) followed by Tukey's post hoc test for each gene. n = 3 biological replicates per time point. P values indicated in the figure.

(C)Representative flow cytometry histograms showing COL10 expression in ATDC5 cells (P5-7) after 21-day chondrogenic differentiation with various treatments. Two-way ANOVA (lentivirus treatment × time point) followed by Tukey's post hoc test for each gene. n = 3 biological replicates per time point. P values indicated in the figure.


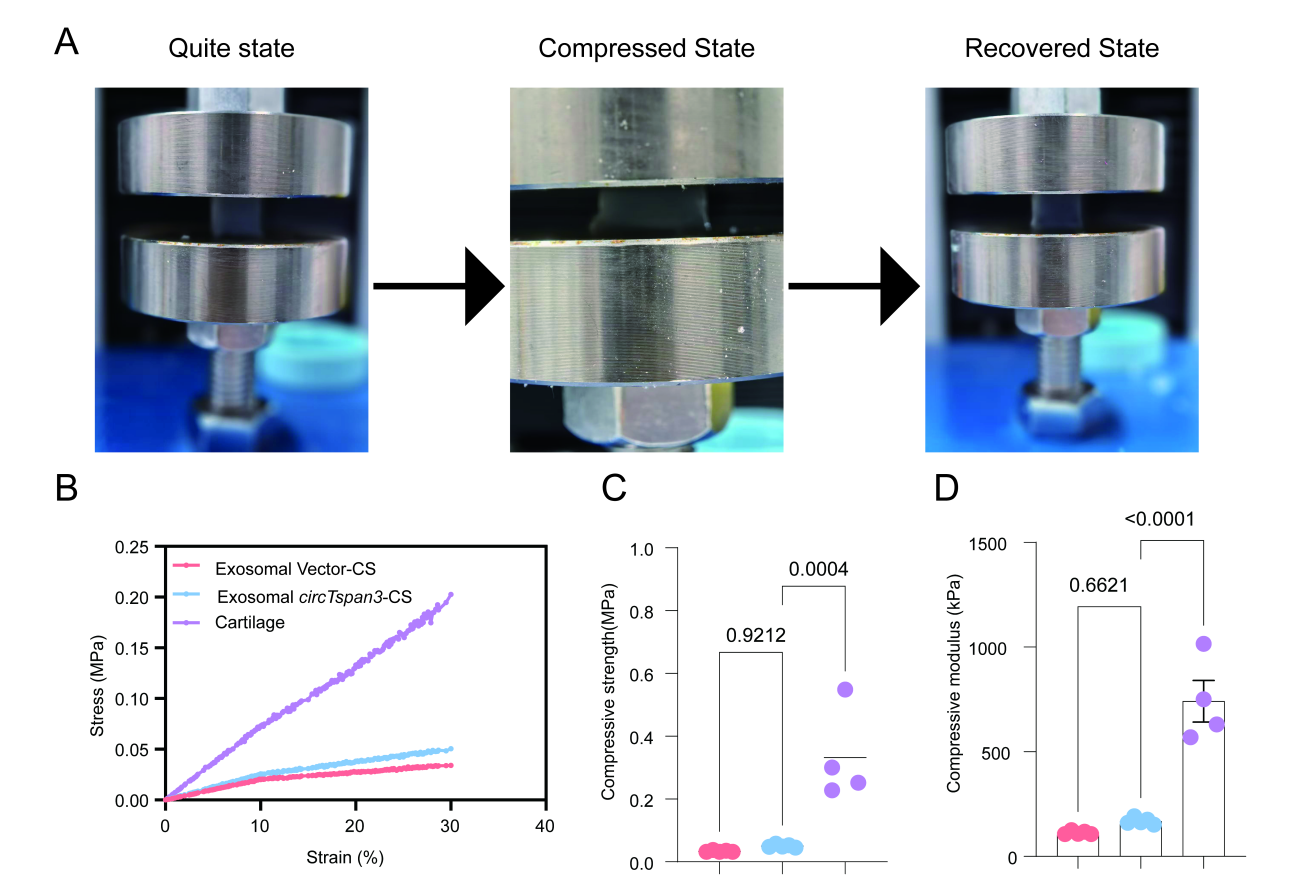


**Figure S17**. Compressive mechanical testing of exosomal *circTspan3*-CS hydrogel.

(A) Representative images of exosome *circTspan3*-chitosan hydrogel compression self-recovery.

(B) Compressive stress-strain response of exosome c*ircTspan3*-chitosan hydrogel.

(C) Quantization diagram of B.

(D) Compressive strength of exosome *circTspan3*-chitosan hydrogel.

Data are presented as mean ± SEM (absolute values, not normalized). Statistical analysis: one-way ANOVA followed by Tukey's post hoc test. n = 4-5 independent samples per group. P values indicated in the figure.


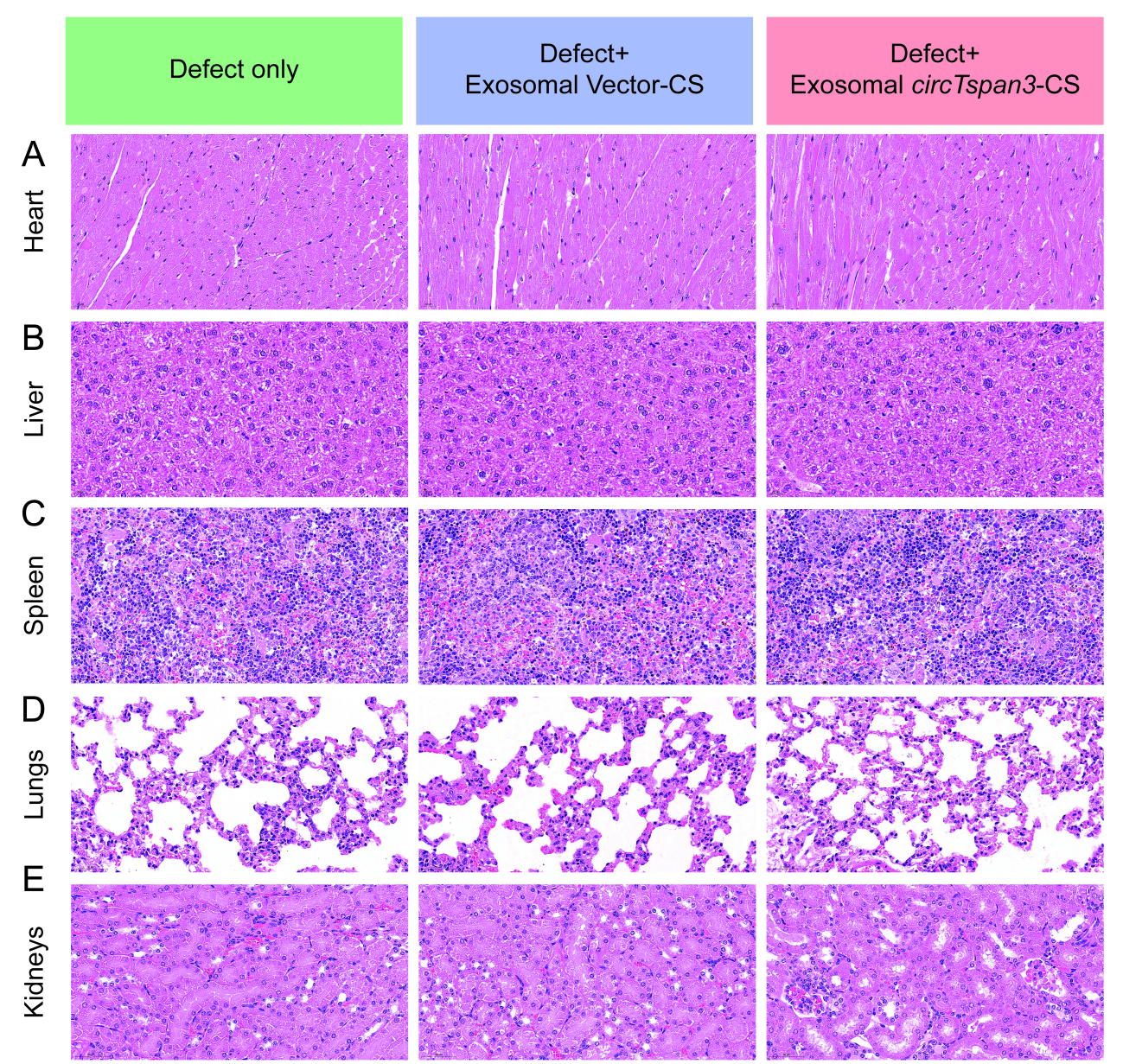


**Figure S18.** Systemic biocompatibility evaluation of Exo-*circTspan3*-chitosan hydrogel.

(A-E) Representative hematoxylin and eosin (H&E) staining of major organs harvested at 8 weeks post-injection from mice in three experimental groups: Defect only, Exo-Vector-CS, and Exo-*circTspan3*-CS. Organs (heart, liver, spleen, lung, kidney) were fixed in 4% paraformaldehyde for 48 hours, embedded in paraffin, sectioned at 0.25 μm thickness, and stained with H&E according to standard protocols. Sections were evaluated by a board-certified veterinary pathologist blinded to treatment groups for signs of toxicity, inflammation, fibrosis, necrosis, or other pathological changes. Representative images from n = 3-5 mice per group per organ.


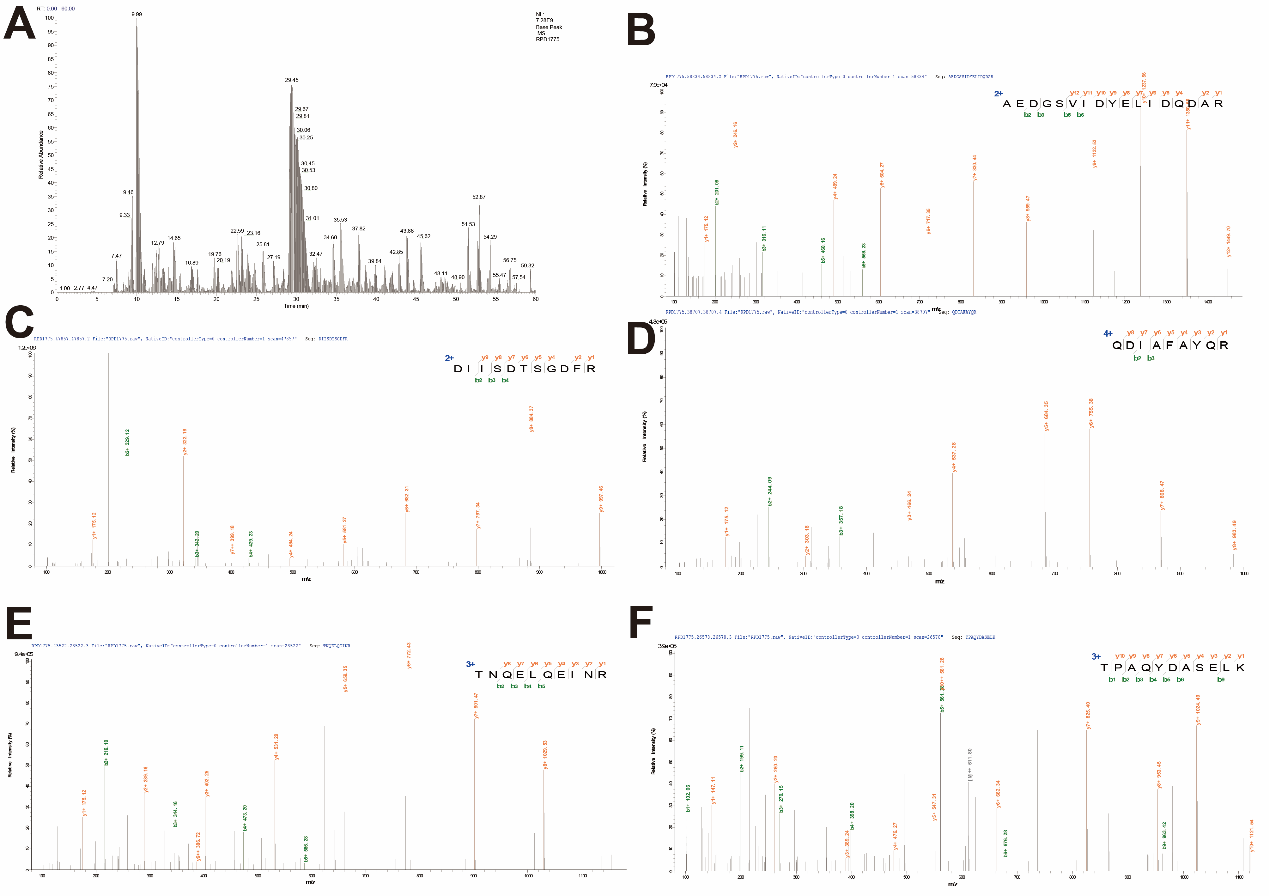


**Figure S19.** Mass spectrometry results of ATDC5 infected with *circTspan3* overexpression lentivirus to screen the target proteins of *circTspan3*.

(A) Mass spectrometry Basepeak plot of overexpression *circTspan3* group.

(B-F) MS/MS spectrum of Annexin A2.


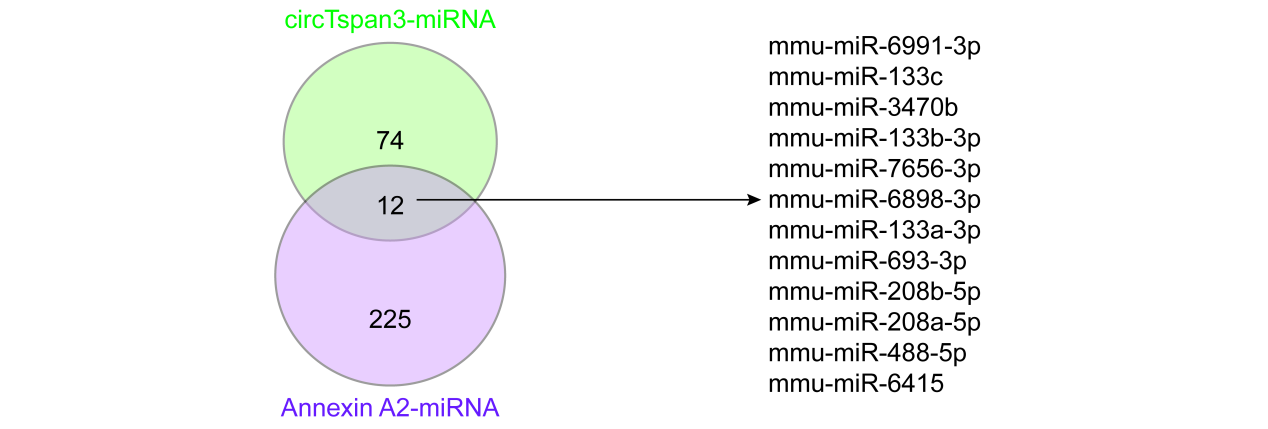


**Figure S20.** Bioinformatic prediction of miRNA-mediated regulatory network involving *circTspan3* and ANXA2.

Venn diagram and table showing 12 miRNAs predicted by miRanda software (sequence match threshold 140, minimum free energy ≤ -10 kcal/mol, strict seed matching) to potentially bind both *circTspan3* and the 3′UTR of *Anxa2* mRNA. Listed miRNAs: mmu-miR-6991-3p, mmu-miR-133c, mmu-miR-3470b, mmu-miR-133b-3p, mmu-miR-7656-3p, mmu-miR-6898-3p, mmu-miR-133a-3p, mmu-miR-693-3p, mmu-miR-208b-5p, mmu-miR-208a-5p, mmu-miR-488-5p, and mmu-miR-6415. Note: these predictions were not experimentally validated in the current study.


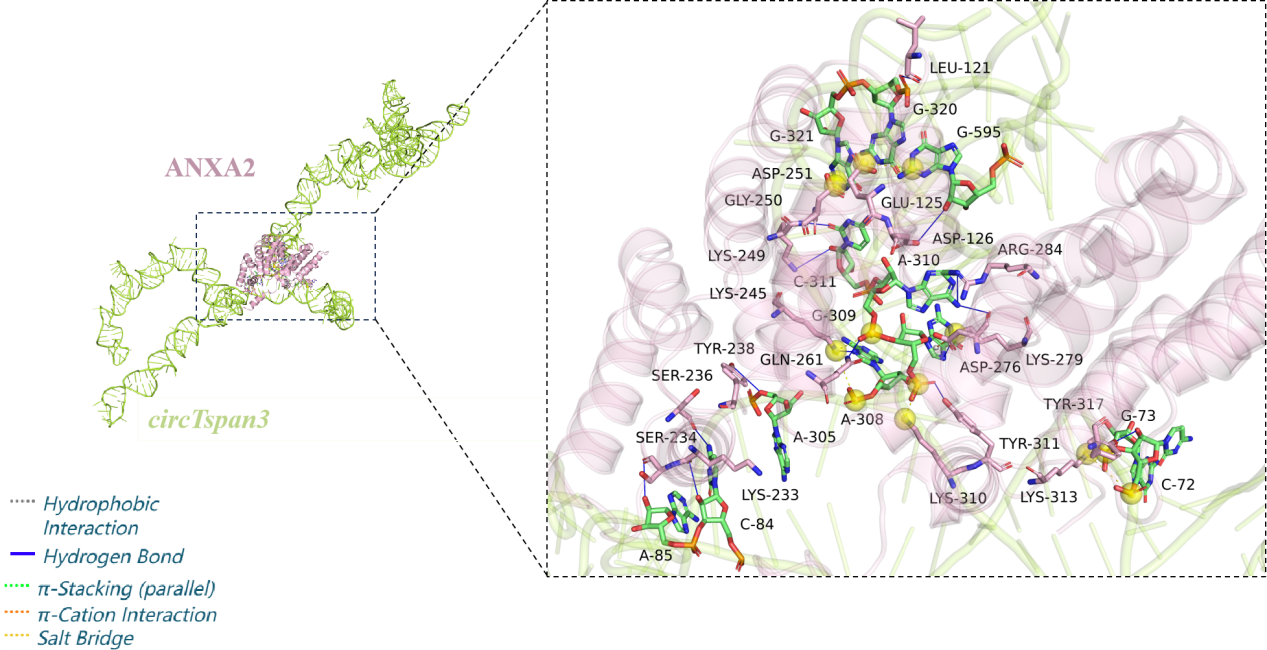


**Figure S21.** Molecular docking prediction of *circTspan3*-ANXA2 interaction.

Molecular docking model generated by HDOCK SERVER and refined with AutoDock Vina, demonstrates multiple hydrogen bonds (dashed lines) and electrostatic interactions between *circTspan3* phosphate groups and ANXA2.


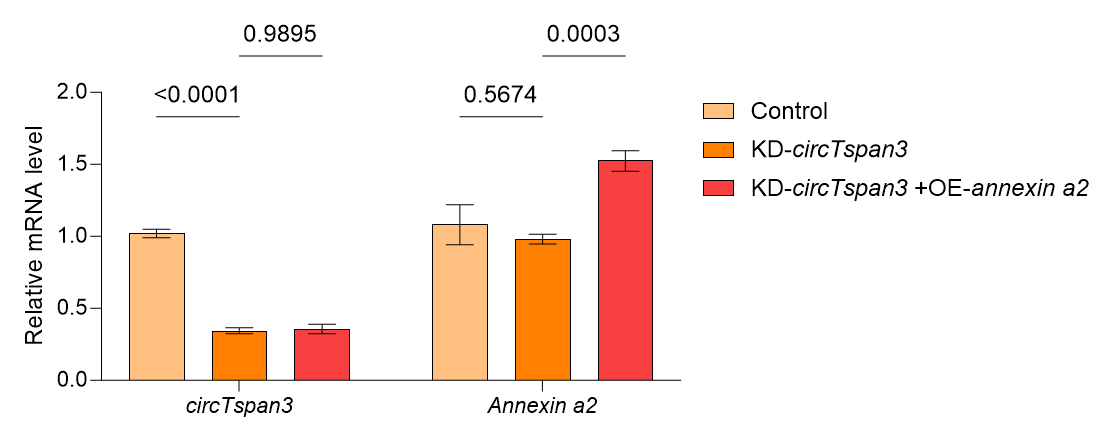


**Figure S22.** Verify the efficiency of KD-*circTspan3* and OE *annexin a2*.

RT-qPCR validation of *Anxa2* overexpression efficiency in ATDC5 cells (P5-7) with *circTspan3* knockdown. *Anxa2* mRNA was normalized to *Gapdh* and expressed as fold change relative to KD-Control group. Data are presented as mean ± SEM. Statistical analysis: one-way ANOVA followed by Tukey's post hoc test. n = 3 biological replicates. P values indicated in the figure.


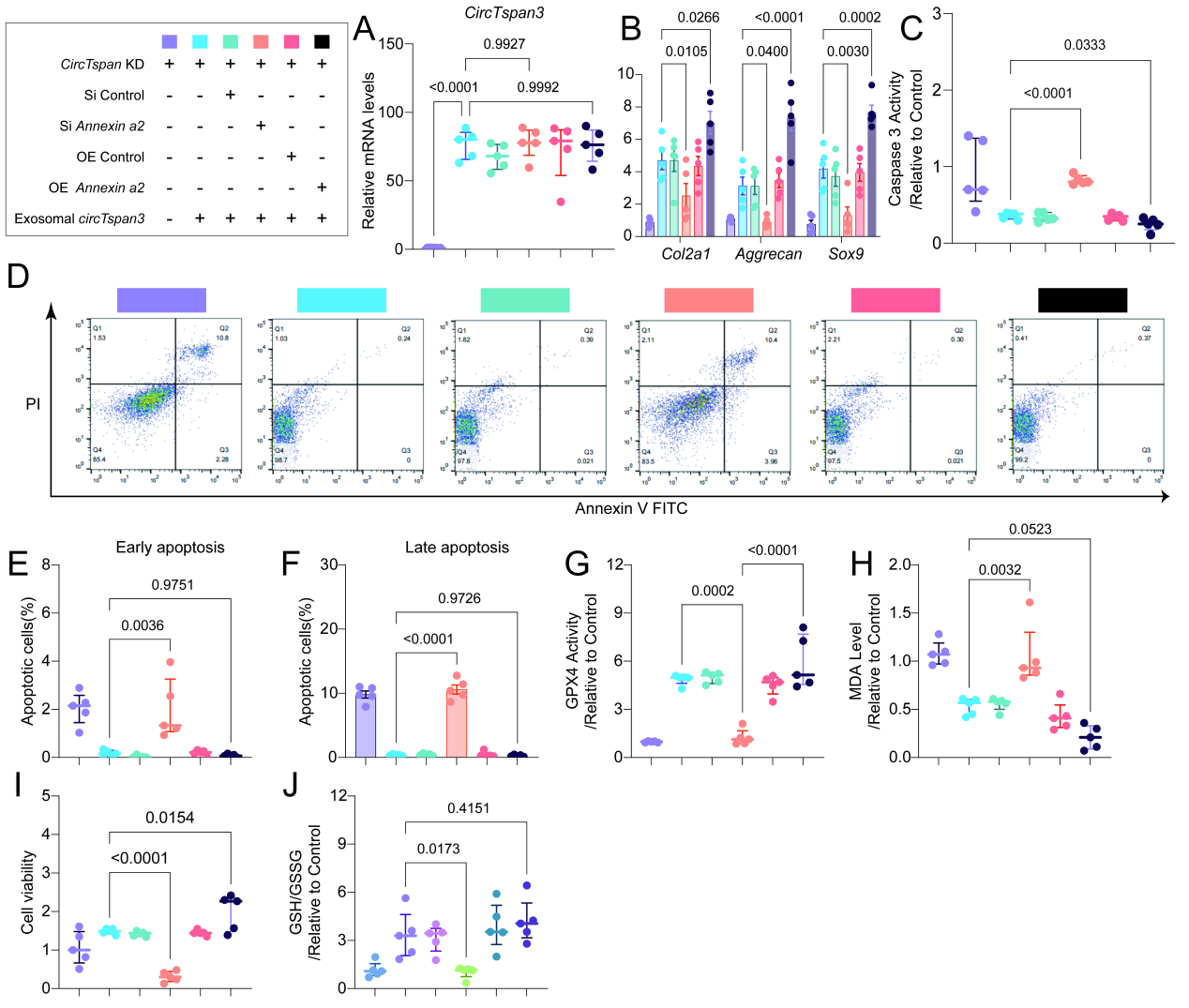


**Figure S23.** ANXA2 mediates the chondroprotective effects of exosomal *circTspan3* through modulation of apoptosis and ferroptosis pathways.

1. Delivery and stability of exosomal *circTspan3* assessed by RT-qPCR quantification of intracellular *circTspan3* levels 48 hours post-treatment.
2. Anabolic marker gene expression (*Col2a1*, *Sox9*, *Aggrecan*) quantified by RT-qPCR in *circTspan3*-knockdown ATDC5 cells treated with exosomal *circTspan3* under ANXA2 overexpression or knockdown conditions.
3. Caspase-3 enzymatic activity measured using cell lysates prepared 48 hours post-treatment.
4. Representative flow cytometry scatter plots showing Annexin V-FITC/propidium iodide (PI) double staining to discriminate apoptotic cell populations. Cells were harvested 48 hours post-treatment, stained according to manufacturer's protocol, and analyzed by flow cytometry (minimum 6,000 events per sample).
5. Quantification of early apoptotic cells (Q4 quadrant, Annexin V⁺/PI⁻) as percentage of total cells. ANXA2 modulation significantly affects the anti-apoptotic efficacy of exosomal *circTspan3*.
6. Quantification of late apoptotic cells (Q2 quadrant, Annexin V⁺/PI⁺) as percentage of total cells, showing similar ANXA2-dependent modulation of exosomal *circTspan3* cytoprotection.
7. Glutathione peroxidase 4 (GPX4) enzymatic activity measured by colorimetric assay (GPX Activity Assay Kit, Cayman Chemical) in cell lysates.
8. Malondialdehyde (MDA) levels quantified by thiobarbituric acid reactive substances (TBARS) assay in cell lysates.
9. Cell viability assessed by Cell Counting Kit-8 (CCK-8) colorimetric assay 48 hours post-treatment. Absorbance at 450 nm is proportional to the number of viable cells.

For panels A-C and G-J, data from each experimental group were first measured as absolute values in appropriate units (ΔΔCt-derived relative expression for RT-qPCR; U/mg protein for enzyme activities; nmol/mg protein for MDA; absorbance at 450 nm for CCK-8; ratio for GSH/GSSG). Statistical comparisons were performed on these original absolute values using one-way analysis of variance (ANOVA) followed by Tukey's honestly significant difference (HSD) post-hoc test. For data visualization in the figure, values were normalized to the *circTspan3* KD group (set to 1.0) by dividing each measurement by the mean of this baseline group, expressing results as fold change. Data are presented as mean ± standard error of the mean (SEM) from n=5 independent biological replicates selected from five total experimental runs. Each biological replicate represents an independent cell culture preparation, transfection, exosome treatment, and measurement performed on different days.For panels E-F (apoptosis quantification), data are presented directly as percentage of total cells (mean ± SEM, n=5) without normalization, and statistical analysis was performed on these percentage values using one-way ANOVA followed by Tukey's post-hoc test.

**Supplementary Table 1 *CircTspan3* full seq and junction seq**

**>mmu_*circTspan3*_full seq |chr 9:56145488-56147578|**

GGGGCAGCTGGCATCCTCTGCTACGTGGGAGCCTATGTCTTCATCACCTATGACGACTATGACCACTTCTTCGAGGATGTGTACACGCTCTTCCCTGCCGTGGTGATCATAGCTGTAGGAGCCTTGCTTTTCATCATTGGCCTGATCGGCTGCTGTGCTACAATCCGGGAGAGCCGCTGTGGACTTGCCACGTTTGTCTTCATCCTGCTCTTGGTTTTCGTCACAGAAGTGGTTGTTGTGGTTTTGGGATACGTGTACAGAGCAAAGGTGGAGAACGAGGTTGATCGCAGCATTCAGAAAGTGTATAAGACCTACAACGGGACCAACTCCGACGCTGCCAGCCGTGCTATTGACTATGTGCAGAGACAGTTGCACTGTTGTGGAATTCATAACTATTCAGACTGGGAAAATACAGATTGGTTCAAAGAAACAAAAAACCAGAGTGTCCCTCTTAGCTGCTGCAGAGAGACTGCCAAAAGCTGTAACGGCAGCCTGGCCAACCCCTCTGACCTCTACGCCGAGGGCTGTGAGGCTCTTGTTGTGAAGAAGCTACAAGAAATCTTGATGCATGTTATCTGGGCAGCGTTGGCCTTTGCAGCTATTCAG

**>mmu_*circTspan3*_junction_seq |chr 9:56145488-56147578|-**

CAGCGTTGGCCTTTGCAGCTATTCAGGGGGCAGCTGGCATCCTCTGCTACGT

**Supplementary Table 3.** **Primer pair for RT-qPCR**

| **Gene-Species** | **Forward**  **Sequence (5' -> 3')** | **Reverse**  **Sequence (5' -> 3')** |
| --- | --- | --- |
| C*ol2a1*-M | CAGGATGCCCGAAAATTAGGG | ACCACGATCACCTCTGGGT |
| A*ggrecan*-M | CCTGCTACTTCATCGACCCC | AGATGCTGTTGACTCGAACCT |
| M*mp*13-M | CTTCTTCTTGTTGAGCTGGACTC | CTGTGGAGGTCACTGTAGACT |
| *Adamts5*-M | GGAGCGAGGCCATTTACAAC | CGTAGACAAGGTAGCCCACTTT |
| S*ox9*-M | GAGCCGGATCTGAAGAGGGA | GCTTGACGTGTGGCTTGTTC |
| *Xbp1s*-M | GCTGAGTCCGCAGCAGGTG | GGTCCAACTTGTCCAGAATGCC |
| *Adamts4*-M | ATGGCCTCAATCCATCCCAG | AAGCAGGGTTGGAATCTTTGC |
| *Gapdh*-M | AGGTCGGTGTGAACGGATTTG | TGTAGACCATGTAGTTGAGGTCA |
| T*span3*-M | GTTGGCCTTTGCAGCTATTC | GTAGGTTCCGCCAGTGATGA |
| *CircTspan3*-M | GGGGCAGCTGGCATCC | CTGAATAGCTGCAAAGGCC |
| *U6*-M | GTGCTCGCTTCGGCAGCACAT | ATATGGAACGCTTCACGAAT |
| *Annexin a2*-M | ATGTCTACTGTCCACGAAATCCT | CGAAGTTGGTGTAGGGTTTGACT |
| *Gpx4*-M | GATGGAGCCCATTCCTGAACC | CCCTGTACTTATCCAGGCAGA |
| *Acsl4*-M | CTCACCATTATATTGCTGCCTGT | TCTCTTTGCCATAGCGTTTTTCT |
| *Ptgs2*-M | TTCAACACACTCTATCACTGGC | AGAAGCGTTTGCGGTACTCAT |
| *Fth1*-M | CAAGTGCGCCAGAACTACCA | GCCACATCATCTCGGTCAAAA |
| *Bax*-M | CCGGCGAATTGGAGATGAACT | CCAGCCCATGATGGTTCTGAT |
| *Bcl2*-M | GAGAGCGTCAACAGGGAGATG | CCAGCCTCCGTTATCCTGGA |
| *Caspase3*-M | TGGTGATGAAGGGGTCATTTATG | TTCGGCTTTCCAGTCAGACTC |
| *Slc7a11*-M | GGCACCGTCATCGGATCAG | CTCCACAGGCAGACCAGAAAA |
| *linear Tspan3* | GACCTGCTGGTGGTGATTTG | CCAGGTAGAGGTTGAGGTTGA |

**Supplementary Table 4. Sequences of probes used in this study**

| **Method** | **Sequences (5′→3′, antisense)** |
| --- | --- |
| FISH | CCAGCTGCCCCCTGAATAGCTG |
| ISH | ATGCCAGCTGCCCCCTGAATAGCTGCAAAG |
| Pull down | ATGCCAGCTGCCCCCTGAATAGCTGCAA |

**Supplementary Table 5. ChIP-qPCR primer sequences (mouse)**

| **Promoter location** | **Forward**  **Sequence (5' -> 3')** | **Reverse**  **Sequence (5' -> 3')** |
| --- | --- | --- |
| Chip-TSPAN3-1 | GACAGGTAAAGAGGTCCCGA | ATGCCACTACCCCTCCC |
| Chip-TSPAN3-2 | GGGGTAGTGGCATGAATGAA | CAACTTTGGGAGTCAGTTCTTT |

**Table 6. Antibody information and dilution ratios used in this study**

| Antibody | Product No./Company | Dilution ratio |
| --- | --- | --- |
| Collagen Type II Polyclonal antibody | 28459-1-AP; Proteintech | IF(1:100), IHC(1:100),WB(1:1500),FC(10 ul per 10^6^ cells in 100 μl suspension) |
| XBP1S-specific Polyclonal antibody | 24868-1-AP; Proteintech | IF(1:50), IHC(1:100), WB(1:1000) |
| GAPDH Polyclonal antibody | 60004-1-Ig; Proteintech | WB(1:10000) |
| Beta Tubulin Polyclonal antibody | 13036-1-AP; Proteintech | WB(1:10000) |
| TSPAN3 Polyclonal antibody | 13036-1-AP; Proteintech | WB(1:2000) |
| SOX9 Monoclonal antibody | 67439-1-Ig; Proteintech | WB(1:1000),FC(5 ul per 10^6^cells in 100 μl suspension) |
| Aggrecan Polyclonal antibody | 13880-1-AP; Proteintech | IHC(1:100),WB(1:1000),FC(5 ul per 10^6^ cells in 100 μl suspension) |
| Annexin A2 Polyclonal antibody | 11256-1-AP; Proteintech | IF(1:50), WB(1:1200) |
| Sox9 Antibody (E-9) | sc-166505; Santa cruz | IHC(1:50), FC(15 ul per 10^6^ cells in 100 μl suspension) |
| Beta Actin Monoclonal antibody | 66009-1-Ig; Proteintech | WB(1:10000) |
| Phospho-Annexin A2 (Ser26) Antibody | AF5440; Affinity | IF(1:100), WB(1:1500) |
| HRP-conjugated Affinipure Goat Anti-Rabbit IgG(H+L) | SA00001-2; Proteintech | WB(1:20000) |
| HRP-conjugated Affinipure Goat Anti-Mouse IgG(H+L) | SA00001-1; Proteintech | WB(1:20000) |
| CD9 Polyclonal antibody | 20597-1-AP; Proteintech | WB(1:2000) |
| TSG101 Polyclonal antibody | 28283-1-AP; Proteintech | WB(1:1000) |
| GRP94 Polyclonal antibody | 14700-1-AP; Proteintech | WB(1:1000) |
| Collagen Type X Polyclonal antibody | 26984-1-AP; Proteintech | IHC(1:50), FC(10 ul per 10^6^ cells in 100 μl suspension) |
| Multi-rAb® CoraLite® Plus 488-Goat Anti-Rabbit Recombinant Secondary Antibody (H+L) | RGAR002; Proteintech | IF(1:100) |
| Multi-rAb® CoraLite® Plus 488-Goat Anti-Mouse Recombinant Secondary Antibody (H+L) | RGAM002; Proteintech | IF(1:100) |
| CoraLite594-conjugated Goat Anti-Mouse IgG(H+L) | SA00013-3; Proteintech | IF(1:100) |
| CoraLite594–conjugated Goat Anti-Rabbit IgG(H+L) | SA00013-4; Proteintech | IF(1:100) |
| Anti-mouse/rabbit universal immunohistochemistry detection kit | PK10006;Proteintech | N/A |

**References**

[1] G. Zhao, Y. Fu, Z. Cai, et al., “Unspliced XBP1 Confers VSMC Homeostasis and Prevents Aortic Aneurysm Formation via FoxO4 Interaction,” *Circ Res* 121, no. 12 (2017): 1331-1345. <https://doi.org/10.1161/circresaha.117.311450>

[2] X. Wen, X. Li, Y. Tang, et al., “Chondrocyte FGFR3 Regulates Bone Mass by Inhibiting Osteogenesis,” *J Biol Chem* 291, no. 48 (2016): 24912-24921. <https://doi.org/10.1074/jbc.M116.730093>

[3] L. Zeng, Y. Li, J. Yang, et al., “XBP 1-Deficiency Abrogates Neointimal Lesion of Injured Vessels Via Cross Talk With the PDGF Signaling,” *Arteriosclerosis, thrombosis, and vascular biology* 35, no. 10 (2015): 2134-2144. <https://doi.org/10.1161/atvbaha.115.305420>

[4] T. Atsumi, Y. Miwa, K. Kimata, and Y. Ikawa, “A chondrogenic cell line derived from a differentiating culture of AT805 teratocarcinoma cells,” *Cell Differ Dev* 30, no. 2 (1990): 109-116. <https://doi.org/10.1016/0922-3371(90)90079-c>

[5] C. Gardiner, Y. J. Ferreira, R. A. Dragovic, C. W. Redman, and I. L. Sargent, “Extracellular vesicle sizing and enumeration by nanoparticle tracking analysis,” *J Extracell Vesicles* 2, no. (2013). <https://doi.org/10.3402/jev.v2i0.19671>

[6] A. Chenite, C. Chaput, D. Wang, et al., “Novel injectable neutral solutions of chitosan form biodegradable gels in situ,” *Biomaterials* 21, no. 21 (2000): 2155-2161. <https://doi.org/10.1016/s0142-9612(00)00116-2>

[7] V. C. Mow, S. C. Kuei, W. M. Lai, and C. G. Armstrong, “Biphasic creep and stress relaxation of articular cartilage in compression? Theory and experiments,” *J Biomech Eng* 102, no. 1 (1980): 73-84. <https://doi.org/10.1115/1.3138202>

[8] J. Radhakrishnan, A. Subramanian, U. M. Krishnan, and S. Sethuraman, “Injectable and 3D Bioprinted Polysaccharide Hydrogels: From Cartilage to Osteochondral Tissue Engineering,” *Biomacromolecules* 18, no. 1 (2017): 1-26. <https://doi.org/10.1021/acs.biomac.6b01619>

[9] M. A. Wesdorp, S. Capar, Y. M. Bastiaansen-Jenniskens, et al., “Intra-articular Administration of Triamcinolone Acetonide in a Murine Cartilage Defect Model Reduces Inflammation but Inhibits Endogenous Cartilage Repair,” *Am J Sports Med* 50, no. 6 (2022): 1668-1678. <https://doi.org/10.1177/03635465221083693>
